# Coupling Nitrogenous Organic Wastewater Treatment and Biorefinery via N‐Cycling Bacterium

**DOI:** 10.1002/advs.202513035

**Published:** 2025-11-14

**Authors:** Ziqian Wang, Chunyu Du, Guanyu Zheng, Dahu Ding

**Affiliations:** ^1^ College of Resources and Environmental Sciences Nanjing Agricultural University Nanjing 210095 China

**Keywords:** life‐cycle assessment, non‐sterile fermentation, PHB biorefinery, techno‐economic analysis, wastewater valorization

## Abstract

The pathway toward circular economy fundamentally overthrows the perspective on wastewater, from a mere waste to a stream encompassing retrievable resources. Nevertheless, reliable and cost‐effective technologies for resource recovery from refractory nitrogenous organic wastewaters (NOWs) are very scarce. Therefore, this study proposes an innovative strategy that couples the bioremediation of NOWs with sustainable production of polyhydroxybutyrate (PHB). Specifically, this study employs the newly discovered *Paracoccus* sp. ZQW‐1 as a “sustainable cell factory”, which exhibits outstanding PHB production capabilities using N‐methylpyrrolidone (NMP) as the specific nitrogen source. Ingeniously, the selective pressure of NMP eliminates the sterilization requirement in PHB biorefinery processes. Capitalizing on this advantage, the conceptual demonstration of simultaneous non‐sterile PHB production (7.71 g L^−1^) and steady‐state wastewater depuration is successfully conducted in a 3‐L bioreactor. More encouragingly, this paradigm has been successfully applied to the valorization of diverse NOWs (e.g., pyridine and N, N‐dimethylformamide), demonstrating its general applicability. Therefore, this study provides a valuable reference for upcycling other NOWs (e.g., aziridine, acridine, pyrrole, and indole). Additionally, life‐cycle assessment and techno‐economic analysis validate the environmental and economic sustainability of this biorefinery process. Overall, this study proposes an innovative biorefinery strategy and offers a forward‐looking trajectory for sustainable wastewater valorization.

## Graphical abstract

The vast reservoir of nitrogenous organic pollutants in industrial wastewaters can serve as untapped carbon‐nitrogen resources. This study employs the newly discovered *Paracoccus* sp. ZQW‐1 as a versatile chassis to simultaneously achieve N‐methylpyrrolidone (NMP) wastewater depuration and polyhydroxybutyrate (PHB) synthesis. Ingeniously, the selective pressure of NMP can suppress microbial competitors, thereby enabling efficient PHB production under non‐sterile conditions.

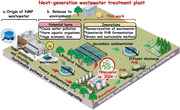

## 1 Introduction

For over a century, wastewater treatment plants (WWTPs) have primarily focused on pollutant removal, with organic matter oxidized to CO_2_, reactive nitrogen converted to dinitrogen gas, and non‐biodegradable substances condensed into solid streams for waste disposal.^[^ ^1^, ^2^ ^]^ Although these strategies serve well to protect ecological security and human health, they fail to realize wastewater valorization.^[^ ^3^, ^4^ ^]^ Recently, WWTPs have been recognized as trinity production facilities, coined as “Nutrient + Energy + Water factories”, and are undergoing a paradigm shift from pollutant removal to resource recovery.^[^ ^5^, ^6^, ^7^ ^]^ Up until now, tremendous efforts have been made to transform WTTPs into energy producers rather than energy consumers to drive the paradigm shift.

Microbial cell factories can upcycle various waste substrates into commodity chemicals (e.g., bioplastics and biopesticides).^[^ ^8^, ^9^, ^10^ ^]^ While chemical recovery from agro‐industrial wastewaters (e.g., sugar factories) has been well‐documented, most studies focus on idealized synthetic wastewaters rather than actual refractory wastewaters, especially nitrogenous organic wastewaters (NOWs).^[^ ^11^, ^12^ ^]^ Notably, the vast reservoir of N‐heterocyclic compounds (NHCs) in NOWs may serve as untapped carbon‐nitrogen resources. However, NHCs can suppress microbial metabolism and restrict resource recovery due to their low bioavailability and inherent cytotoxicity.^[^ ^13^, ^14^ ^]^ More seriously, the inefficient elimination and continual accumulation of NHCs in WWTPs can pose escalating threats to human health and environmental safety.^[^ ^15^ ^]^ Consequently, it is essential to develop innovative strategies to overcome the fundamental trade‐off between NHCs treatment and resource recovery, simultaneously achieving wastewater depuration and value extraction.

Benefiting from unique nitrogen metabolic pathways and potential biosynthetic capacities, N‐cycling microorganisms may serve as “sustainable cell factories” to capture heterocyclic nitrogen fluxes from wastewater and funnel metabolic intermediates into bioproducts.^[^ ^7^ ^]^ Among others, polyhydroxyalkanoates (PHA) have emerged as sustainable alternatives to petroleum‐derived plastics, whose fermentation processes are highly dependent on nitrogen substrates and sterile conditions.^[^ ^16^, ^17^ ^]^ Notably, NHCs may provide essential nitrogen for PHA synthesis while their inherent cytotoxicity and low bioavailability may effectively suppress microbial competitors, thereby overturning the mandatory sterilization requirement in conventional PHA fermentation.^[^ ^18^, ^19^ ^]^ Therefore, re‐imagining the conversion pathways of NHCs in wastewater by developing multifunctional N‐cycling microorganisms may achieve a win‐win situation for environmental protection and economy.^[^ ^20^ ^]^ Nevertheless, transitioning NOWs management to this forward‐looking trajectory remains challenging due to the lack of ideal chassis cells.

Here, we applied the newly discovered *Paracoccus* sp. ZQW‐1 as a versatile chassis to redirect heterocyclic N fluxes of N‐methylpyrrolidone (NMP) wastewater toward PHA synthesis. Innovatively, we achieved deep mineralization and efficient detoxification of NMP wastewater (90.7% reduction in total organic carbon (TOC)) and synchronously recovered 7.71 g L^−1^ polyhydroxybutyrate (PHB). More importantly, the selective pressure imposed by NMP confers a competitive advantage to *Paracoccus* sp. ZQW‐1, thereby eliminating the sterilization requirements in conventional PHB fermentation. Capitalizing on this advantage, a 3‐L scale continuous fermentation was successfully conducted under non‐sterile conditions to show the feasibility of this paradigm. More encouragingly, this paradigm has been successfully applied to the remediation and valorization of other NOWs (e.g., pyridine (PYD), N, N‐dimethylformamide (DMF), and N, N‐dimethylacetamide (DMAC)), indicating its general applicability. In addition, developing an innovative wastewater valorization strategy necessitates a comprehensive assessment encompassing technical feasibility, environmental sustainability, and economic viability. Therefore, life‐cycle assessment (LCA) and techno‐economic analysis (TEA) were conducted, which revealed that this wastewater biorefinery strategy exhibited lower greenhouse gas (GHG) emissions and compelling economic benefits. By developing a cost‐competitive and environmentally beneficial approach for sustainable PHB production from NOWs, we provide a potential bridge between refractory wastewater remediation and circular bioeconomy.

## 2 Results

### 2.1 Bioremediation for NMP Wastewater

**Figure 1:**
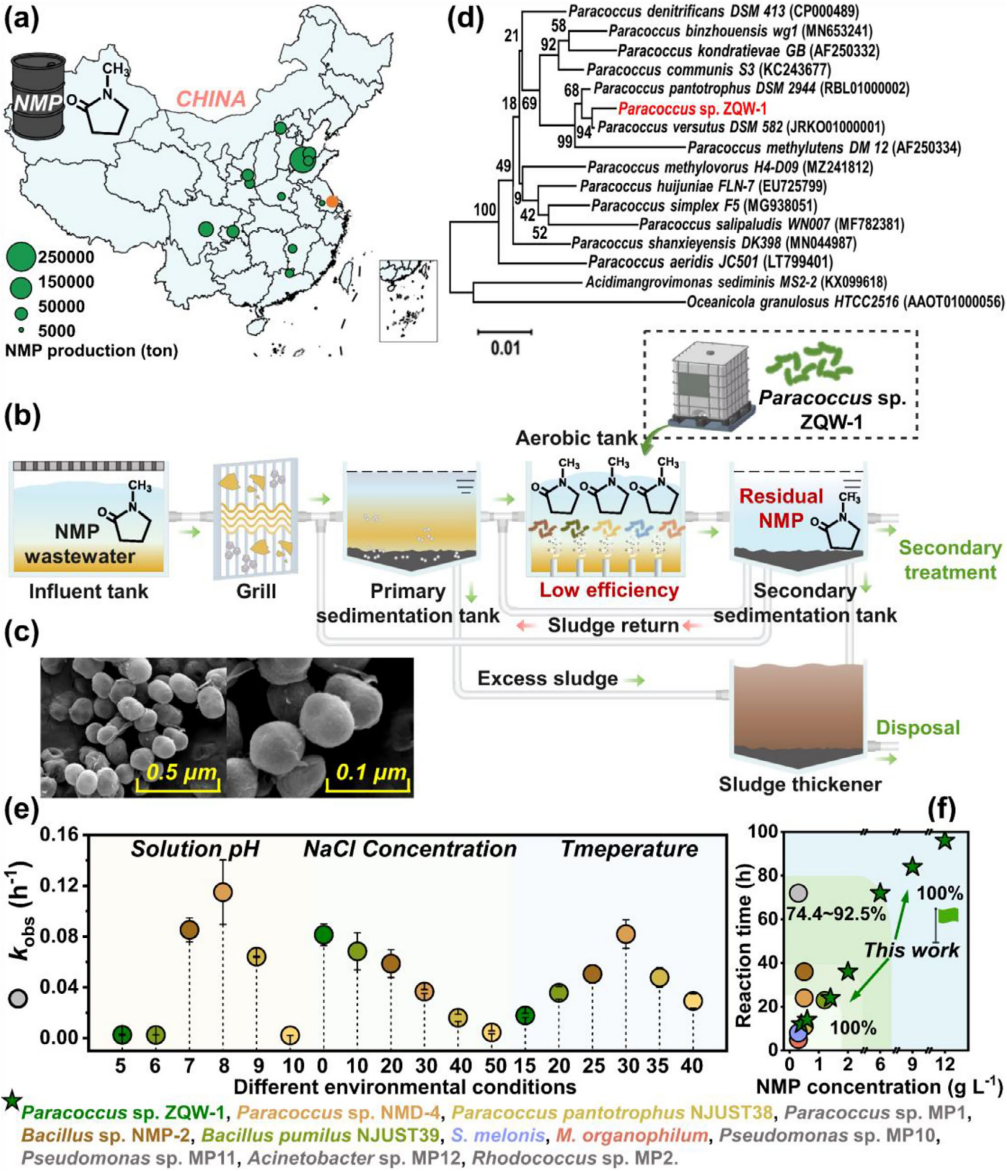
Bioremediation performance of *Paracoccus* sp. ZQW‐1 for NMP wastewater treatment. a) The representative NMP manufacturers and productivity distribution in China. b) Oriented bioaugmentation for NMP wastewater depuration. c) SEM images of *Paracoccus* sp. ZQW‐1. d) Phylogenetic tree of *Paracoccus* sp. ZQW‐1. e) NMP degradation under different environmental conditions. f) Comparison of NMP degradation by *Paracoccus* sp. ZQW‐1 with other documented NMP degrading strains. Experimental conditions: [NMP] = 1 g L^−1^, [inoculum concentration] = 4%, [pH] = 5−10, [NaCl] = 0−50 g L^−1^, [temperature] = 15−40 °C. All flask data were performed in triplicates.

N‐Methylpyrrolidone, as a five‐membered NHCs, has been applied in diverse industries (e.g., electronics, agrochemicals, and pharmaceuticals). As depicted in **Figure** **1**a, the NMP production capacity of thirteen representative NMP production enterprises in China mainly ranges from 5000 to 250 000 tons per year. Given the excellent water solubility of NMP (> 1000 g L^−1^), high‐concentration NMP wastewater is often generated during manufacturing processes (> 1000 mg L^−1^).^[^ ^15^ ^]^ For instance, ≈30 g L^−1^ NMP wastewater is produced during NMP recycling processes in a New Materials Technology Co., Ltd. in ZhenJiang (the red dot in map), causing a huge burden on the sewage treatment system (Table S1, Supporting Information). However, traditional biotechnologies could not efficiently remediate the high‐concentration NMP wastewater due to its inherent biotoxicity and chemical stability.^[^ ^21^ ^]^ Consequently, additional pretreatment methods (e.g., catalytic ozone oxidation) are necessary, significantly increasing the operating cost. Substitutably, bioaugmentation is deemed a more feasible and cost‐effective option (Figure 1b).^[^ ^22^ ^]^ To develop cost‐effective wastewater remediation strategies, we first acquired a versatile chassis that could efficiently metabolize NMP and accumulate PHA (Figure 1c; Figure S1, Supporting Information, strain ZQW‐1). The phylogenetic tree revealed that it was close to *Paracoccus* strains (e.g., *Paracoccus pantotrophus* DSM 2944 and *Paracoccus versutus* DSM 582) (Figure 1d). Likewise, it shared high similarity with *Paracoccus versutus* DSM 17099 (98.4%) and *Paracoccus pantotrophus* DSM 2944 (91.2%) based on the average nucleotide identity (ANI), confirming that strain ZQW‐1 belonged to *Paracoccus* species (Figure S2, Supporting Information). After that, the optimum culture condition for *Paracoccus* sp. ZQW‐1 was determined (i.e., 200 rpm, pH 8, and 5 g L^−1^ NaCl) according to growth curves and growth rate constant (Figure S3, Supporting Information, *k* = 0.409 h^−1^).

Notably, *Paracoccus* sp. ZQW‐1 rapidly degraded 1 g L^−1^ NMP within 24 h while exhibiting robust growth kinetics (Figure S4, Supporting Information). Moreover, we investigated the adaptive capacity of *Paracoccus* sp. ZQW‐1 to wastewater parameter variations. Satisfactorily, it exhibited stable removal efficiency across a wide NMP concentration (Figure S5, Supporting Information, 0.2 to 2.0 g L^−1^) and well adapted to the variations in pH (7−9, *k*_obs_ = 0.064−0.115 h^−1^), NaCl concentration (0−30 g L^−1^, *k*_obs_ = 0.037−0.082 h^−1^) and temperatures (25 to 35 °C, *k*_obs_ = 0.048−0.082 h^−1^) (Figure 1e; Figure S6, Supporting Information). More encouragingly, *Paracoccus* sp. ZQW‐1 maintained excellent degradability toward actual NMP wastewater and rapidly eliminated 0.5−2 g L^−1^ NMP within 36 h, revealing its superior capability to resist the potential inhibitors in actual NMP wastewater (Figure S7, Supporting Information). In particular, we compared the NMP degradation by *Paracoccus* sp. ZQW‐1 and other documented NMP degrading strains (Table S2, Supporting Information). As depicted in Figure 1f, NMP degrading strains such as *Paracoccus* sp. MP1, *Pseudomonas* sp. MP10, and *Rhodococcus* sp. MP2 exhibited limited efficiency in low‐concentration NMP wastewater treatment (0.3 g L^−1^), with a removal rate of 74.4−92.5% over 72h.^[^ ^23^ ^]^ In contrast, *Paracoccus* sp. ZQW‐1 completely degraded low‐concentration NMP wastewater (0−0.6 g L^−1^) within 14 h, comparable to *Paracoccus pantotrophus* NJUST38 (0.5 g L^−1^ within 11 h), *Sphingomonas melonis* (0.3 g L^−1^ within 8 h), and *Methylobacterium organophilum* (0.3 g L^−1^ within 5 h).^[^ ^24^, ^25^ ^]^ Meanwhile, *Paracoccus* sp. ZQW‐1 was able to treat moderate‐concentration NMP wastewater (1.4 g L^−1^) within 24 h, rivaling the state‐of‐the‐art *Bacillus pumilus* NJUST39 (1.2 g L^−1^ within 23 h).^[^ ^26^ ^]^ More remarkably, *Paracoccus* sp. ZQW‐1 maintained exceptional degradation performance toward high‐concentration (2−6 g L^−1^ within 72 h) and even ultra‐high‐concentration actual NMP wastewater (6−15 g L^−1^ within 108 h), underscoring its unparalleled robustness (Figure S8, Supporting Information). Consequently, *Paracoccus* sp. ZQW‐1 emerges as a promising candidate for industrial wastewater remediation due to the excellent adaptability to complex environmental conditions.

### 2.2 Detoxification of NMP wastewater

**Figure 2:**
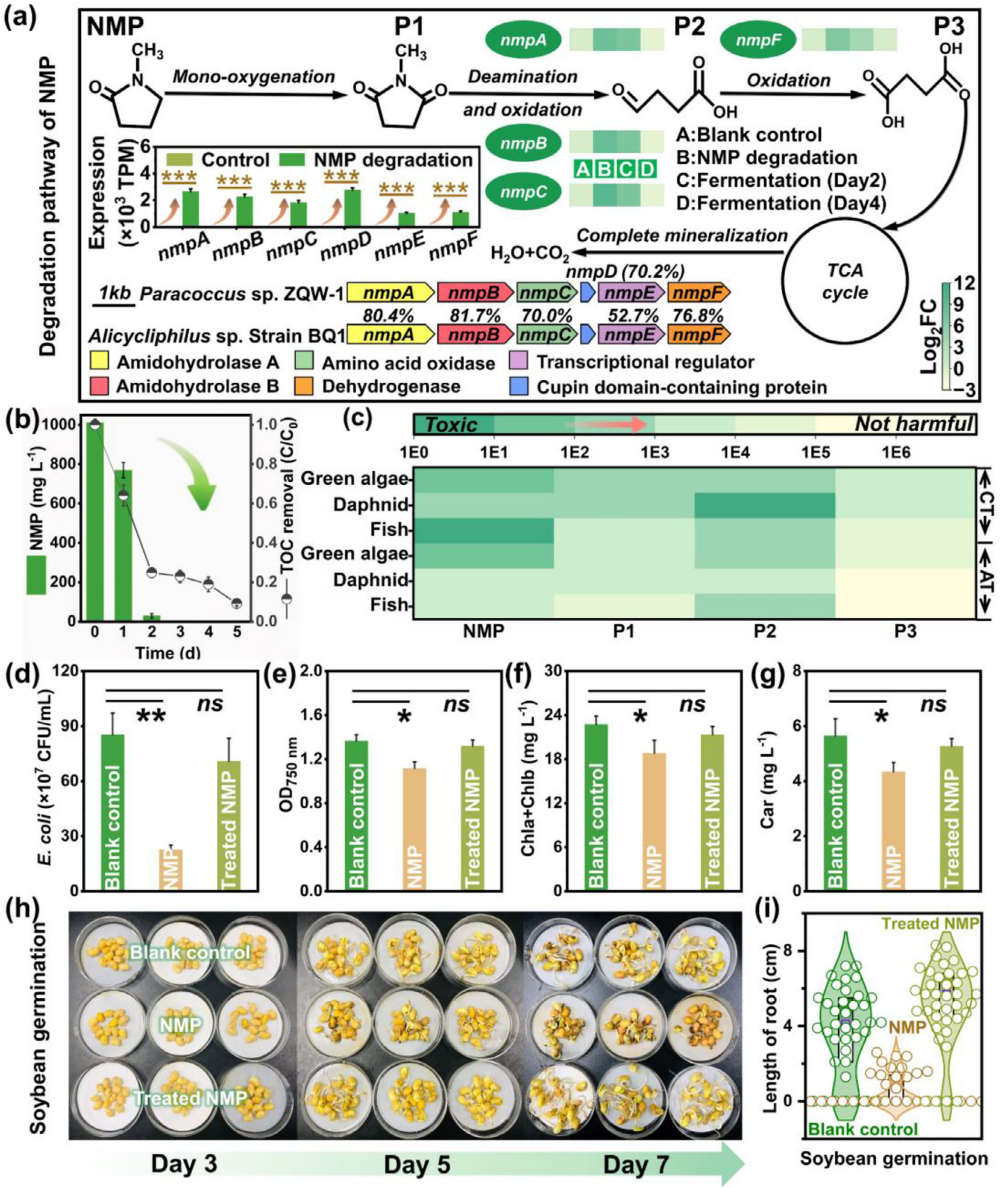
Mechanism elucidation and environmental compatibility assessment of NMP biodegradation. a) The biodegradation pathway of NMP. Control, strain cultivation stage. b) TOC removal during NMP degradation. c) Ecological environmental risk assessment via Ecological Structure‐Activity Relationships with acute toxicity (AT) and chronic toxicity (CT) as evaluation metrics. d,e) Culture of *E. coli* XL1‐Blue and *Chlorella vulgaris* in different water matrixes. f,g) Chlorophyll (Chla and Chlb) and carotenoid (Car) accumulation. h,i) Soybean seed germination and root length distribution. The water matrixes included pure water (Blank control), NMP wastewater, and treated NMP wastewater. All data were performed in triplicates. ns represents *p* > 0.05. * represents 0.01 < *p* < 0.05. ** represents *p* < 0.01.

Notably, deciphering the toxicity evolution of NMP degradation intermediates was essential to ensure effective bioremediation. The biodegradation pathway of NMP was proposed based on intermediate recognition, genome and transcriptome analysis (Figures S9−S11, Supporting Information). As shown in **Figure** **2**a, a six‐gene cluster (i.e., *nmpABCDEF*) was identified, which could be transcribed as a polycistronic mRNA and encoded enzymes for NMP biodegradation (Table S3, Supporting Information).^[^ ^27^ ^]^ The encoding products shared relatively high amino acid identities with those of *Alicycliphilu*s sp. strain BQ1 (52.7–81.7%), indicating the similar NMP biodegradation pathway. As expected, the expression levels (TPM) of *nmpABCDEF* were significantly up‐regulated by 102.5−690.8 times during NMP degradation stage (Figure 2a, inset). Therefore, NMP was first oxidized to 1‐methyl‐2,5‐pyrrolidinedione (P1), and then hydrolyzed and deaminated to form succinic semialdehyde (P2) by n‐methylhydantoin amidohydrolase (*nmpA* and *nmpB*) and amino acid oxidase (*nmpC*).^[^ ^21^, ^28^ ^]^ The succinic semialdehyde was further transformed to succinic acid (P3) via succinate semialdehyde dehydrogenase (*nmpF*), subsequently entering the tricarboxylic acid (TCA) cycle for mineralization into H_2_O and CO_2_.

Noticeably, some toxic intermediates may originate from incomplete NMP mineralization. Encouragingly, more than 90% of TOC was eliminated even with ultra‐low inoculation (Figure 2b, 0.1%), superior to *Paracoccus pantotrophus* NJUST38 (78.6%) and UV‐assisted Fenton process (only 26.9%).^[^ ^25^, ^29^ ^]^ Moreover, the eco‐toxicity of NMP‐derived intermediates was predicted through Ecological Structure Activity Relationships Program (ECOSAR). Although P2 exhibited relatively high toxicity levels, it was converted to P3 as the reaction progressed, thereby mitigating the eco‐toxicity (Figure 2c). Furthermore, systematic toxicity assessments on bacterial growth and plant germination were conducted to fully capture the real‐world implications of such treatment. As evidenced in Figure 2d, NMP inhibited the growth of *E. coli* XL1‐Blue, whereas the inhibition was significantly alleviated after treatment. Likewise, NMP had negative effects on *Chlorella vulgaris* growth as well as chlorophyll and carotenoid accumulation, which were recovered to normal levels after detoxification treatment (Figure 2e−g; Figures S12 and S13, Supporting Information). In addition, the germination rate of soybean seeds exposed to NMP wastewater was much lower than that of blank group (Figure 2h, 36.0% vs 80.0 %). In contrast, the seeds cultured with treated NMP wastewater achieved impressive germination rates of 89.0 % (Figure S14, Supporting Information). Similar trends were observed in root length measurements (Figure 2i). The average root length of seeds cultivated in treated NMP wastewater was 5.1 cm, significantly surpassing those treated with pure water (3.7 cm) and NMP wastewater (0.6 cm). The enhanced root growth might be attributed to the breakdown of NMP into some smaller molecules, serving as additional nutrients for seed development.^[^ ^30^ ^]^ Therefore, *Paracoccus* sp. ZQW‐1 achieved efficient NMP mineralization and toxicity elimination, validating its bioremediation capacity.

### 2.3 Development of Sustainable Wastewater Valorization Strategies

As mentioned above, *Paracoccus* sp. ZQW‐1 sustained high degradation efficiency even at high NMP concentrations, which may be attributed to PHA‐mediated stress response mechanisms.^[^ ^31^ ^]^ In response to adverse conditions, *Paracoccus* sp. ZQW‐1 may synthesize PHA to enhance its resilience.^[^ ^32^ ^]^ The complete central carbon metabolism (e.g., glycolysis and pyruvate metabolism) may provide essential precursors (Acetyl‐CoA) and ATP for PHA synthesis (Figures S15 and S16, Supporting Information).^[^ ^33^, ^34^ ^]^ Consistent with genomic predictions, we detected PHB accumulation during NMP degradation. As shown in Figure S17, Supporting Information, 126.8 mg L^−1^ PHB was produced during NMP (2 g L^−1^) degradation, which demonstrated that *Paracoccus* sp. ZQW‐1 could directly bioconvert NMP into PHB. Considering the low C/N ratio of NMP, appropriate organics were supplemented to redirect Acetyl‐CoA from energy generation to PHB synthesis (Figure S18, Supporting Information).^[^ ^35^ ^]^ Notably, these organics may acquire from other industrial wastewater such as molasses wastewater (containing glucose and sucrose) and biodiesel wastewater (containing glycerol) in the future, which may further improve the economic viability of wastewater valorization technologies.^[^ ^13^ ^]^

**Figure 3:**
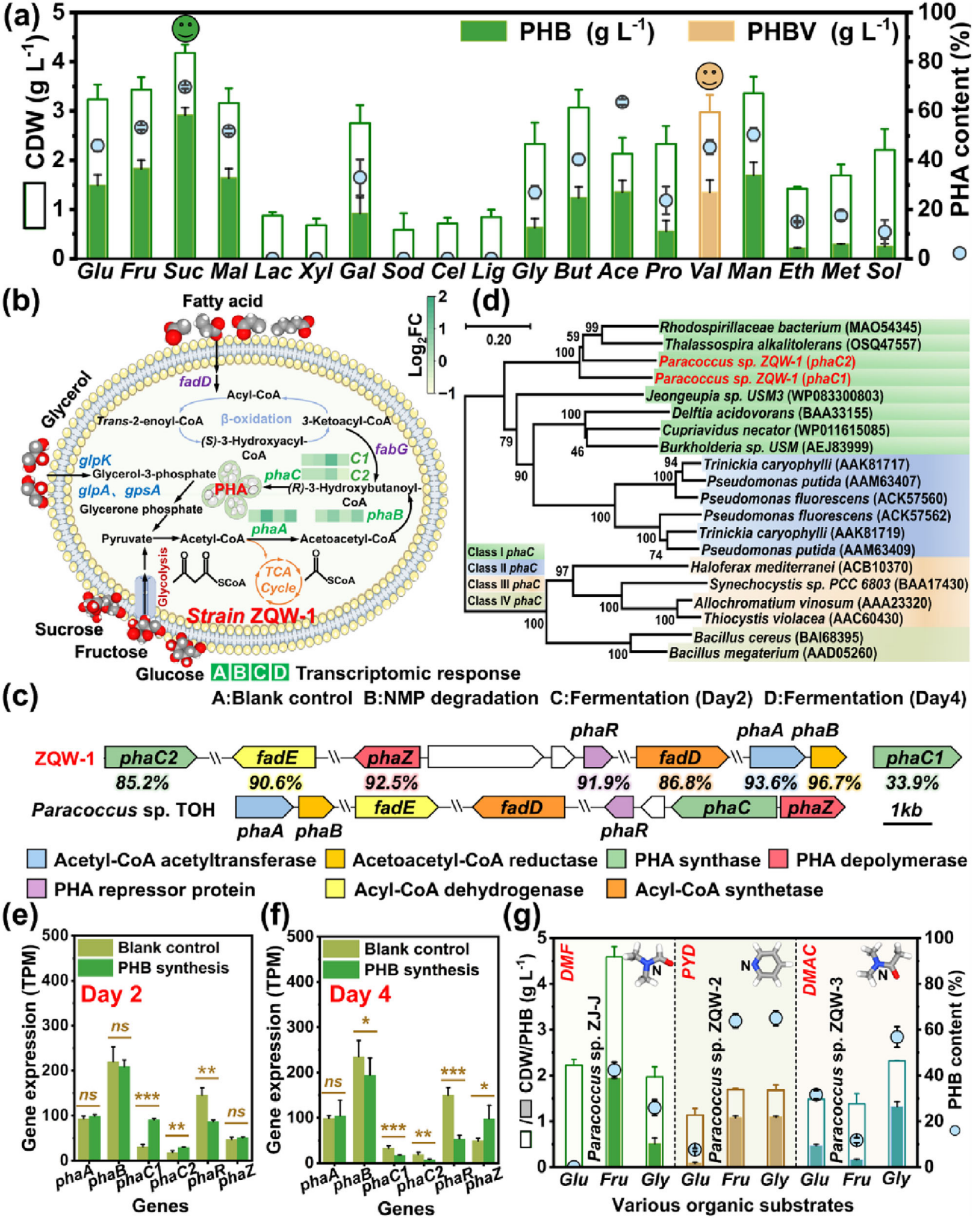
Process optimization and mechanistic elucidation of PHA synthesis from nitrogenous organic wastewaters. a) Recovery of PHA from NMP wastewater supplemented with various organics. Glu, glucose; Fru, fructose; Suc, sucrose; Mal, maltose; Lac, lactose; Xyl, xylose; Gal, galactose; Sod, sodium citrate; Cel, cellulose; Lig, lignin; Gly, glycerol; But, butyric acid; Ace, acetic acid; Pro, propionic acid; Val, valeric acid; Man, mannitol; Eth, ethanol; Met, methanol; Sol, soluble starch. Experimental conditions: [organics] = 10 g L^−1^, [C/N] = 40, [pH] = 8, [fermentation time] = 2d, [inoculation dosage] = 4%. b) The metabolic pathways of PHA synthesis. c) The gene cluster responsible for PHA synthesis. d) The phylogenetic tree of PHA synthase (*phaC*). e,f) The gene expression levels at strain cultivation stage (Blank control), PHB accumulation stage (The second day of fermentation), and PHB consumption stage (The fourth day of fermentation). g) The PHB production from diverse nitrogenous organic wastewaters. The characteristic pollutants included PYD, DMF, and DMAC. All data were performed in triplicates. ns represents *p* > 0.05. * represents 0.01 < *p* < 0.05. ** represents 0.001 < *p* < 0.01. *** represents *p* < 0.001.

As depicted in **Figure** **3**a, nineteen organics were used to improve PHB production, among which the maximum PHB titer (2.92 g L^−1^) was obtained using sucrose as the substrate (Table S4, Supporting Information). Notably, *Paracoccus* sp. ZQW‐1 could also produce poly(3‐hydroxybutyrate‐co‐3‐hydroxyvalerate) (PHBV) by using valeric acid as the substrate. After process optimization, *Paracoccus* sp. ZQW‐1 attained unprecedented PHB productivity (4.19 g L^−1^, Figures S19−S24 and Tables S5−S11, Supporting Information), outperforming previously documented *Paracoccus* strains (0.12−3.77 g L^−1^, Figure S25 and Table S12, Supporting Information). Meanwhile, *Paracoccus* sp. ZQW‐1 achieved a comparable PHB titer (4.17 g L^−1^) by using actual NMP wastewater as the nitrogen source, thereby demonstrating its remarkable stability and making it a promising chassis cell for wastewater valorization (Figure S26, Supporting Information). In particular, to evaluate the metabolic burden of NMP on *Paracoccus* sp. ZQW‐1, we compared the PHB production by using NMP and other non‐toxic nitrogen sources (e.g., (NH_4_)_2_SO_4_, NH_4_Cl, NaNO_3_, NaNO_2_, and CH_4_N_2_O). As depicted in Figure S23 (Supporting Information), superior PHB production (4.19 g L^−1^) was achieved by using NMP as the selective nitrogen source compared to non‐toxic nitrogen sources (2.28−3.95 g L^−1^). This outstanding fermentation performance can be attributed to the inherent robustness and high NMP affinity of *Paracoccus* sp. ZQW‐1, which effectively mitigates the metabolic burden associated with NMP utilization. Notably, serial subculturing experiments were conducted to evaluate the long‐term genetic and productive stability of *Paracoccus* sp. ZQW‐1 under the continuous selective pressure, which was essential for industrial applications. As depicted in Figure S27 (Supporting Information), the key performance metrics, including PHB titer and NMP removal efficiency, were monitored during each subculture process. Encouragingly, *Paracoccus* sp. ZQW‐1 exhibited robust NMP degradation performance in 10 continuous flask‐fermentation cycles, sustaining a high removal efficiency (98.4−99.7%). Meanwhile, *Paracoccus* sp. ZQW‐1 maintained robust PHB production (4.01−4.20 g L^−1^) in 5 continuous flask‐fermentation cycles, indicating its good productive stability. The gradual decline of PHB titer (from 3.97 to 3.61 g L^−1^) in subsequent fermentation cycles may be ascribed to the strain degeneration from repeated subculturing, which may be improved by genetic and process engineering in the future. Beyond productive stability, we also verified the genetic stability of *Paracoccus* sp. ZQW‐1. As expected, the key genes responsible for PHB synthesis (e.g., *phaC*) and NMP degradation (e.g., *nmpB*) were successfully amplified, validating the genetic stability of *Paracoccus* sp. ZQW‐1 (Figure S27, Supporting Information). Therefore, the fermentation process can eliminate the need for repeated seed culture preparation, thereby saving time and operational costs.

Moreover, the PHB synthesis mechanisms and representative PHB biosynthetic pathways were elucidated (Figure 3b; Table S13, Supporting Information). The first pathway began with glycolysis (e.g., glucose and sucrose), while the formed pyruvate was transformed to essential metabolites (e.g., *(R)*‐3‐hydroxyacyl‐CoA) for PHB synthesis. In another metabolic pathway, 3‐ketoacyl‐CoA was derived from fatty acids via the fatty acid β‐oxidation pathway, serving as precursors for *(R)*‐3‐hydroxyacyl‐CoA production.^[^ ^36^ ^]^ Then, *(R)*‐3‐hydroxyacyl‐CoA was converted to PHB by *phaC*. Additionally, *Paracoccus* sp. ZQW‐1 could metabolize glycerol into glycerol 3‐phosphate by glycerol kinase (*glpK*), which was further converted into pyruvate through glycerol‐3‐phosphate dehydrogenase (e.g., *glpA* and *gpsA*) for PHB production. Correspondingly, we detected a complete PHB synthetic gene cluster and the encoding products shared ultra‐high amino acid identities with those in short‐chain‐length PHB (scl‐PHB)‐producing strain *Paracoccus* sp. TOH (85.2−96.7%), indicating the potential to produce scl‐PHB with similar properties (Figure 3c). Meanwhile, another plasmid‐borne *phaC1* was also identified as Class I PHA synthase responsible for scl‐PHB synthesis and may improve PHB polymerization efficiency (Figure 3d).

Furthermore, we specifically deciphered the dynamic interplay between NMP degradation and PHB synthesis based on transcriptional analysis (Figures S28−S32, Supporting Information). Despite significant upregulation of Acetyl‐CoA acetyltransferase (*phaA*) and Acetoacetyl‐CoA reductase (*phaB*), the non‐significant *phaC* expression resulted in low PHB yield using NMP as sole carbon and nitrogen sources (Figure S33, Supporting Information). Notably, the presence of sucrose suppressed the expression of PHB synthesis repressor (*phaR*) and elevated *phaC1* and *phaC2* expression by 2.93 and 1.62 times during PHB accumulation stage (Day 2), thereby boosting PHB yield (Figure 3e). To validate the transcriptomic findings, the expression levels of key PHB biosynthetic genes (i.e., *phaA*, *phaB*, and *phaC*) were quantified by RT‐qPCR analysis. As shown in Figure S34 (Supporting Information), a strong correlation was observed between the RT‐qPCR and transcriptome results, thereby confirming the critical role of *phaC* in PHB synthesis and the reliability of transcriptomic findings. In contrast, PHB synthesis genes were down‐regulated while PHB depolymerase (*phaZ*) was significantly induced at PHB consumption stage (Day 4), matching well with the experimental phenomenon (Figure 3f; Figure S23, Supporting Information). Special attention was paid to the expression of *nmpABCDEF* during PHB production. Apparently, the expressions of *nmpABCDEF* were remarkably up‐regulated by 21.8–169.9 times at PHB accumulation stage (Day 2) and thus *Paracoccus* sp. ZQW‐1 could efficiently utilize NMP as the specific nitrogen source for PHB synthesis, even better than conventional nitrogen sources (Figures S23 and S35, Supporting Information). Notably, the expressions of *nmpABCDEF* were down‐regulated at PHB consumption stage (Day 4) due to the complete removal of NMP, and the resultant nitrogen deficiency constrained PHB accumulation (Figure S36, Supporting Information). These findings demonstrated that NMP could function as both nitrogen donor and metabolic regulator, where its catabolism directly influenced PHB synthesis by modulating nitrogen flux and transcriptional activity. To further validate the role of NMP degradation genes in PHB biosynthesis, a *nmpB* knockout mutant of *Paracoccus* sp. ZQW‐1 was successfully constructed and named *Paracoccus* sp. ZQW‐1‐Δ*nmpB*. As shown in Figure S37 (Supporting Information), *Paracoccus* sp. ZQW‐1‐Δ*nmpB* could still efficiently synthesize PHB using sucrose and conventional nitrogen sources as substrates, confirming that the biosynthetic pathways of PHB were intact. However, *Paracoccus* sp. ZQW‐1‐Δ*nmpB* lost the ability to degrade NMP (Figure S38, Supporting Information). Meanwhile, *Paracoccus* sp. ZQW‐1‐Δ*nmpB* was no longer able to synthesize PHB by using NMP as the specific nitrogen source, which substantiated the critical role of NMP degradation genes in directing metabolic flow from NMP to PHB (Figures S37 and S38, Supporting Information). In addition, to validate the general applicability of this fermentation strategy, we developed more chassis cells capable of utilizing nitrogenous organic pollutants (e.g., PYD, DMAC, and DMF) for PHB synthesis. As depicted in Figure 3g, *Paracoccus* sp. ZJ‐J can efficiently utilize DMF as the specific nitrogen source and rapidly accumulate 1.94 g L^−1^ PHB within 3 days. Likewise, PYD and DMAC were successfully applied for PHB synthesis by using *Paracoccus* sp. ZQW‐2 and *Paracoccus* sp. ZQW‐3 as chassis cells, confirming the general applicability of this fermentation strategy.

### 2.4 Conceptual Demonstration

Considering the inherent cytotoxicity and limited bioavailability of NMP, the proliferation of microbial competitors might be significantly suppressed by NMP‐induced selective pressure, thereby ensuring efficient PHB production by *Paracoccus* sp. ZQW‐1 under non‐sterile conditions.

**Figure 4:**
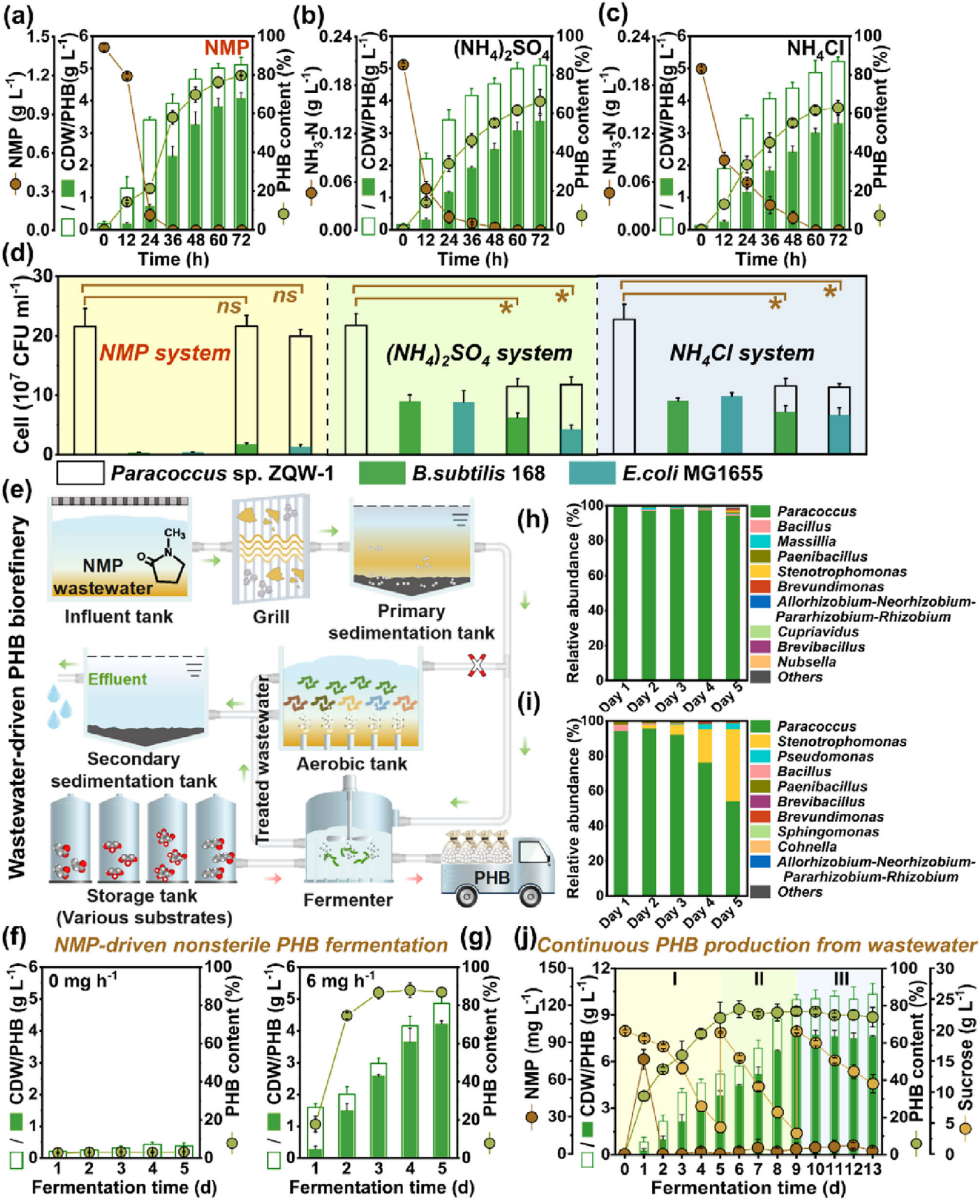
PHB recovery from NMP wastewater under non‐sterile conditions. a−c) Non‐sterile PHB production in conical flasks (50 mL). d) Co‐culture competition among three microbes. e) Conceptual diagram of wastewater valorization in WWTPs. f,g) Semi‐continuous PHB production using synthetic NMP wastewater as the specific nitrogen source in bioreactors (1 L, without sterilization). h,i) Microbial community evolutions of NMP and (NH_4_)_2_SO_4_‐driven PHB fermentation systems. j) Continuous PHB recovery from actual NMP wastewater in bioreactors (3 L, without sterilization). All fermentation data were performed in triplicates. ns represents *p* > 0.05. * represents 0.01 < *p* < 0.05.

To confirm this speculation, PHB fermentation was conducted in non‐sterile medium. Encouragingly, 4.07 g L^−1^ PHB was obtained using NMP as the specific nitrogen source, comparable to that produced under sterilized conditions (**Figure** **4**a). In contrast, the PHB titers were significantly reduced by 14.7% and 16.3% using (NH_4_)_2_SO_4_ and NH_4_Cl as nitrogen sources, respectively (Figure 4b,c). Moreover, two microbial competitors (*B. subtilis* 168 and *E. coli* MG1655) were introduced for co‐culture experiments. Both *B. subtilis* 168 and *E. coli* MG1655 rapidly propagated in (NH_4_)_2_SO_4_ (8.9 × 10^7^ and 8.8 × 10^7^ CFU mL^−1^) and NH_4_Cl (9.0 × 10^7^ and 9.8 × 10^7^ CFU mL^−1^) based fermentation systems, competitively suppressing *Paracoccus* sp. ZQW‐1 and resulting in at least a 43% reduction in PHB titer (Figure 4d; Figure S39, Supporting Information). Advantageously, NMP served well as the bacteriostatic barrier, selectively promoting the dominance of *Paracoccus* sp. ZQW‐1 while maintaining efficient PHB synthesis (Figure 4d). To further validate the ecological competitiveness of *Paracoccus* sp. ZQW‐1, a more rigorous challenge experiment was conducted by introducing a small percentage of activated sludge into the fermentation system. As shown in Figure S40 (Supporting Information), despite the inoculation of 0.4% activated sludge, the abundance of *Paracoccus* sp. ZQW‐1 rapidly increased from 79.4% to 93.3% on the first day of fermentation. Concurrently, a rapid increase in the abundance of NMP‐degrading genes (*nmpA* and *nmpB*) was observed, which enabled *Paracoccus* sp. ZQW‐1 to efficiently utilize NMP and outcompete the microbial competitors. More strikingly, even when the inoculum concentration of activated sludge was increased to 8%, *Paracoccus* sp. ZQW‐1 still exhibited a distinct competitive advantage. As depicted in Figure S40 (Supporting Information), the synchronous increase in the abundance of *Paracoccus* sp. ZQW‐1 and NMP‐degrading genes ensured the ecological competitiveness and functional dominance of *Paracoccus* sp. ZQW‐1 under the selective pressure imposed by NMP. This competitive advantage was attributed to the innate NMP‐degrading capacity of *Paracoccus* sp. ZQW‐1. As depicted in Figure S41 (Supporting Information), *Paracoccus* sp. ZQW‐1‐Δ*nmpB* exhibited normal growth in LB medium despite the deletion of *nmpB* gene. Nevertheless, the growth of *Paracoccus* sp. ZQW‐1‐Δ*nmpB* was significantly inhibited upon NMP addition, whereas *Paracoccus* sp. ZQW‐1 was unaffected. These results demonstrated that the NMP degradation genes were essential for *Paracoccus* sp. ZQW‐1 to resist the selective pressure imposed by NMP, thereby ensuring its competitive advantage and robustness.

To achieve convenient industrial fermentation, we provided a forward‐looking trajectory for wastewater upgrading. The physically‐pretreated NMP wastewater was diverted into the fermenter for PHB biorefinery (Figure 4e). Subsequently, the readily biodegradable fermentation effluent was well‐suited for biological treatment, thereby achieving simultaneous organic removal, resource recovery, and energy saving. To validate this paradigm, a semi‐continuous PHB production process was established in a 1‐L fermenter by using simulated NMP wastewater as the nitrogen source (Figure S42, Supporting Information). Noticeably, appropriate NMP feed rates were required to maintain the balance between NMP metabolization and PHB synthesis. As depicted in Figure 4f,g, *Paracoccus* sp. ZQW‐1 could accumulate 4.22 g L^−1^ PHB at a steady state at the feed rate of 6 mg h^−1^ NMP. Meanwhile, *Paracoccus* sp. ZQW‐1 maintained ultra‐high abundance (>94.4%) throughout the fermentation process, thereby enabling efficient PHB production under non‐sterile conditions (Figure 4h). The bacterial metabolism might be disturbed under the lower feed rates, while the higher feed rates might lead to C/N ratio imbalance and toxicity enhancement, and thus inhibiting the PHB synthesis (Figure S43, Supporting Information). In contrast, some microbial competitors (e.g., *Stenotrophomonas* (41.2%) and *Pseudomonas* (3.3%)) rapidly propagated in the (NH_4_)_2_SO_4_‐driven PHB production process, suggesting the fragility of the fermentation system (Figure 4i). Therefore, the fermentation process was prone to collapse even though the feed rates of (NH_4_)_2_SO_4_ was meticulously regulated (Figures S44 and S45, Supporting Information). Similarly, we demonstrated the competitive advantage of *Paracoccus* sp. ZQW‐1 by tracking the dynamics of the microbial community through metagenomic analyses. As depicted in Figure S46 (Supporting Information), *Paracoccus* sp. ZQW‐1 maintained a high relative abundance (93.8−94.8%) in the NMP‐driven fermentation system. Meanwhile, the abundance of PHB biosynthetic gene (*phaC*) remained stable, thereby enabling efficient PHB accumulation. In contrast, the abundance of both *Paracoccus* sp. ZQW‐1 and the *phaC* gene dropped significantly in the (NH_4_)_2_SO_4_‐driven fermentation system due to the competitive advantage of *Stenotrophomonas* (90.4%). Furthermore, the semi‐continuous PHB production was successfully carried out with actual NMP wastewater, achieving a comparable PHB titer of 4.24 g L^−1^ at an NMP feed rate of 6 mg h^−1^ (Figures S47 and S48, Supporting Information). More importantly, we further realized continuous PHB recovery from actual NMP wastewater in a 3‐L bioreactor (Figure S49, Supporting Information). The maximum PHB titer reached 7.71 g L^−1^ and NMP was efficiently degraded throughout the process, which underscored the robustness of *Paracoccus* sp. ZQW‐1 and the scalability of the biorefinery process (Figure 4j). Based on these successful attempts, we further applied this non‐sterile fermentation strategy to valorize other nitrogenous organic wastewaters. As shown in Figure S50 (Supporting Information), *Paracoccus* sp. ZQW‐2 efficiently accumulated 1.51 g L^−1^ PHB at a PYD feed rate of 1 mg h^−1^, which was superior to that obtained with (NH_4_)_2_SO_4_ (1.07 g L^−1^), indicating that PYD can also effectively suppress the growth of microbial competitors. Likewise, we achieved continuous PHB production (4.47 g L^−1^) and efficient depuration of actual PYD wastewater under non‐sterile conditions (Figure S51, Supporting Information). Therefore, these findings demonstrated the feasibility of PHB production from nitrogenous organic wastewaters using N‐cycling microorganisms and underscored the general applicability of the non‐sterile fermentation strategy.

### 2.5 Life‐Cycle Assessment and Techno‐Economic Analysis

**Figure 5:**
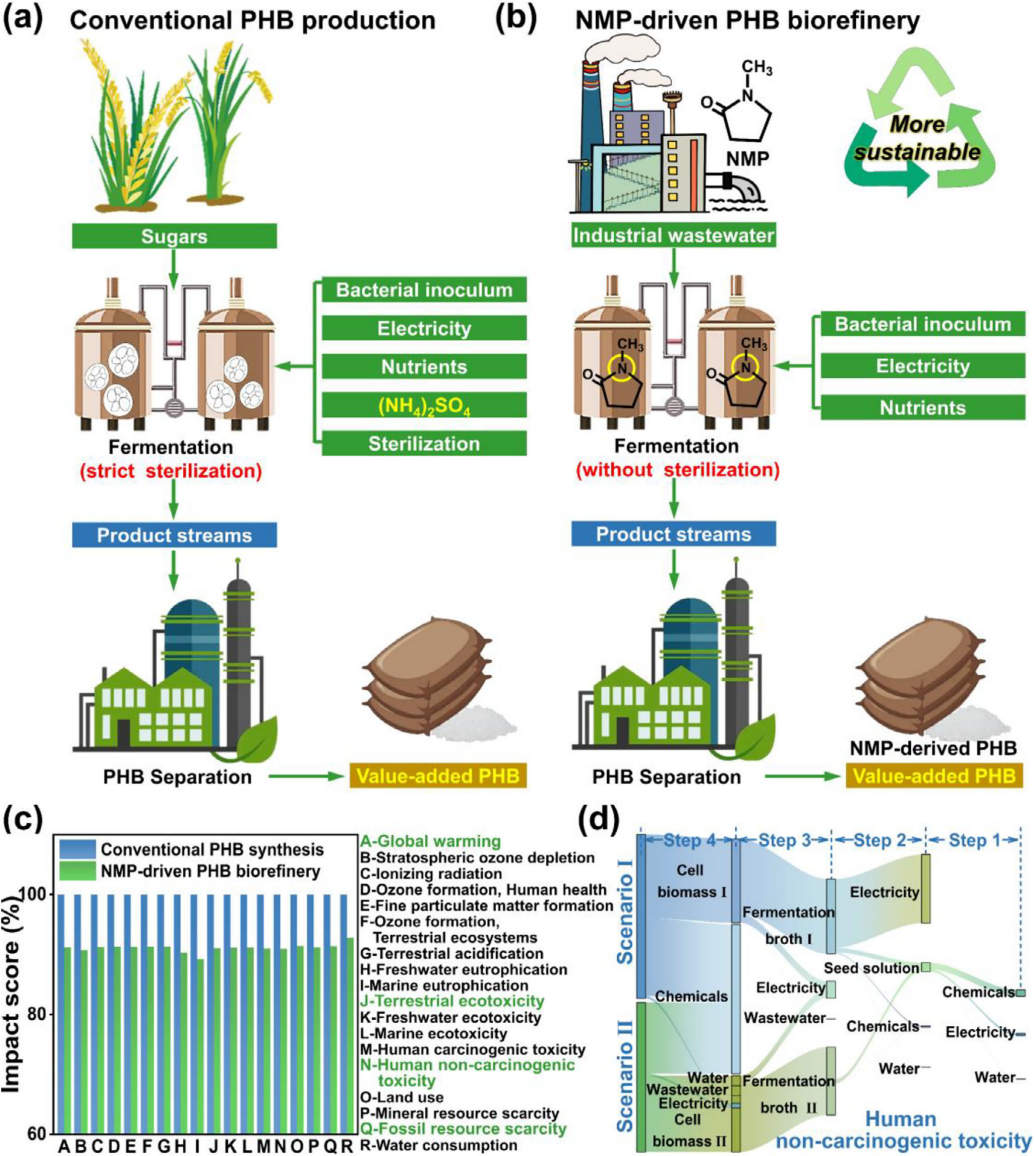
Environmental impacts of wastewater‐driven PHB biorefinery. a,b) Conceptual diagrams of conventional PHB production (Scenario I) and NMP wastewater‐driven PHB biorefinery (Scenario II). c) Environmental impact scores of two production processes. d) Specific contributions of each production step for human non‐carcinogenic toxicity.

To provide optimization strategies for future industrial expansion, LCA and TEA were employed to evaluate the environmental impacts and economic feasibility of the wastewater upgrading process (**Figure** **5**a,b).^[^ ^37^ ^]^ Prior to these analyses, the physicochemical characteristics of wastewater‐derived PHB were systematically investigated, which was crucial for realistically assessing its economic potential and market value (Figure S52, Supporting Information). As evidenced by ^1^H NMR, the wastewater‐derived PHB possessed similar chemical structure and monomer composition with the commercial PHB standard (sigma). A representative peak at 5.27 ppm was observed, which was attributed to the methine group (CH). Meanwhile, another two peaks (i.e., methylene (CH_2_) and methyl (CH_3_) groups) located at 2.6 and 1.3 ppm were also detected (Figure S53, Supporting Information). As shown in Figure S54 (Supporting Information), a distinct absorption band attributed to C = O groups (1724 cm^−1^) was successfully detected by FTIR, which may determine the biodegradability and mechanical properties of the PHB.^[^ ^38^ ^]^ Another two representative peaks assigning to the stretching vibration of CH_3_ and CH_2_ groups were observed at 2967 and 2933 cm^−1^, matching well with the commercial PHB product. Furthermore, the thermal properties, especially the melting temperature (*T*_m_) and crystallinity (*X*_c_), were determined according to the DSC analysis. As evidenced by Figure S54 (Supporting Information), the *T*_m_ of wastewater‐derived PHB was 173.6 °C, which was comparable to that of the commercial PHB product (176.6 °C). The *X*_c_ of wastewater‐derived PHB was determined to be 48.9%, which was lower than that of the commercial PHB (64.1%). This low crystallinity may endow it with superior processability and tunability for various applications.^[^ ^39^ ^]^ Meanwhile, the thermal decomposition behavior of PHB was determined by TGA, focusing mainly on the temperature of 5% weight loss (*T*_d5_). Likewise, the *T*_d5_ of wastewater‐derived PHB (269.6 °C) was comparable to that of the commercial PHB (270.6 °C). Nevertheless, the extracted PHB exhibited incomplete decomposition, leaving ≈3.5% residues. Therefore, the recovery yield and purity of the PHB were further calculated and determined to be 92.8% and 96.3%, respectively. While this purity level may be sufficient for conventional packaging, it may be inadequate for biomedical applications due to the potential impurities.^[^ ^40^ ^]^ These residual impurities (e.g., lipopolysaccharides, lipids, and peptidoglycan) may be derived from the bacterial biomasses and affect the properties of PHB products.^[^ ^41^ ^]^ Therefore, further downstream purification processes (e.g., solvent washing and ultrafiltration) may be necessary to eliminate the residual impurities. Notably, the wastewater‐derived PHB belonged to stiff materials, as evidenced by the tensile strength (11.6 MPa) and elongation at break (12.8 %), matching well with previously documented PHB materials (Table S14, Supporting Information).^[^ ^40^ ^]^ Therefore, it is necessary to incorporate plasticizers or blend with other materials (e.g., PLA) to further enhance its mechanical properties, thereby meeting the requirements for industrial applications. Moreover, the weight average molecular weight (M_w_, 295749 g mol^−1^) and polydispersity index (PDI, 3.18) of wastewater‐derived PHB were determined (Figure S55, Supporting Information). Obviously, the NMP wastewater‐derived PHB exhibited a relatively low M_w_ among various documented PHB materials (62 000−1 335 340 g mol^−1^), which may endow it with excellent degradability (Table S15, Supporting Information).^[^ ^42^ ^]^ As shown in Figure S56 (Supporting Information), the PHB film degraded slowly in soil within the first 120 days, while its degradation became obvious over the next 120 days, as confirmed by the increased weight loss (from 11.7% to 84.3%) and degradation kinetic rate (from 0.001 to 0.015 d^−1^). The slow degradation during initial 120 days was probably due to microbial adaptation, gradual depolymerases accumulation, and dense surface of PHB. In contrast, the polystyrene (PS) film was negligibly degraded over the same degradation period. Consequently, even if non‐recyclable PHB products were released to environments, they would ultimately be decomposed. Importantly, the CO_2_ released from PHB decomposition could re‐enter the biological carbon cycle, thereby avoiding the net carbon emissions to atmosphere.^[^ ^43^ ^]^ Therefore, these findings confirmed that the wastewater‐derived PHB exhibited comparable material properties to commercial PHB, thereby providing a basis for LCA and TEA analyses.^[^ ^44^ ^]^

The system boundaries were depicted in Figures S57 and S58 (Supporting Information), encompassing four main steps (i.e., chassis cell cultivation (step 1), PHB fermentation (step 2), cell separation (step 3), and PHB extraction (step 4)), with 18 midpoint indicators (Tables S16–S22, Supporting Information). Compared with conventional PHB production (Scenario I), the NMP wastewater‐driven PHB biorefinery (Scenario II) can operate stably under non‐sterile conditions, thereby eliminating the sterilization requirement in step 2. While the NMP wastewater required pretreatment by simple filtration, this step utilized a very small amount of filter paper, with no energy consumption or chemical inputs. Consequently, the environmental burden of the upstream pretreatment process was negligible as compared to the four main production stages mentioned above. Furthermore, as the data for filter paper were unavailable in the database, this pretreatment step was excluded from the LCA model.^[^ ^45^, ^46^ ^]^ As depicted in Figure 5c, NMP wastewater‐driven PHB biorefinery possessed much lower environmental impacts than those of Scenario I across all categories. Among others, global warming (GW) potential, human non‐carcinogenic toxicity (HNCT), terrestrial ecotoxicity (TE), and fossil resource scarcity (FRS) were four impactful indicators (Figure S59, Supporting Information). Notably, the values of GW (8.25 kg CO_2_ eq), TE (4.59 kg 1,4‐DCB), HNCT (4.95 kg 1,4‐DCB), and FRS (1.36 kg oil eq) were significantly reduced by 8.61–9.02% in Scenario II, confirming the enhanced environmental sustainability. Moreover, we analyzed the flow diagram of those contributors to reveal the future direction for sustainable PHB production (Figure 5d; Figures S60−S66, Supporting Information). Although the electricity for PHB fermentation was significantly reduced (especially HNCT), the organic solvents (e.g., chloroform) used for downstream PHB extraction substantially contributed to the impact scores (Figure 5d, 47.5%). Similarly, these chemicals were main contributors to CO_2_ emission (25.7%), and thus some cost‐effective extraction methods (e.g., detergents‐based cell lysis) were still urgently needed (Figure S60, Supporting Information).

In addition, the economic feasibility of wastewater upgrading processes was evaluated (Table S23, Supporting Information). The estimated treatment cost of NMP wastewater was 1.44 $ ton^−1^, comparable to the state‐of‐the‐art benchmark (<3.45 $ ton^−1^). Although the calculated breakeven point of PHB (7.28 $ kg^−1^) was not highly competitive in current bioplastic market (5−6 $ kg^−1^), it outperformed previously documented wastewater‐based PHB recovery processes. For instance, the minimum selling price of PHB recovered from secondary urban wastewater by *Synechocystis* sp. R2020 was 146 $ kg^−1^, 20‐times higher than this study due to its low PHB productivity (12.5 g PHB m^−3^ d^−1^).^[^ ^47^ ^]^ Likewise, the production cost of PHB from food processing wastewater using activated sludge was estimated to be 11.8 $ kg^−1^, well above our breakeven point.^[^ ^48^ ^]^ To enhance the economic competitiveness of PHB products, sensitivity analyses were conducted, which can evaluate the effects of key parameter variations on overall economic viability.^[^ ^49^ ^]^ Specifically, the cell dry weight (±20%), PHB content (±10%), and carbon source dosage (±20%) were analyzed. The cell dry weight and PHB content were found to be critical factors for improving process economics and reducing the breakeven point of PHB.^[^ ^50^, ^51^ ^]^ For instance, increasing the PHB content from 85.8% to 95.8% can reduce the breakeven point by ≈11.5%. Likewise, when the cell dry weight rose from 4.94 to 5.93 g L^−1^, the breakeven point decreased by 17.6%. Therefore, future studies should prioritize the development of novel high cell density culture techniques to simultaneously enhance the cell dry weight and PHB content.^[^ ^52^ ^]^ In addition, the sensitivity analysis revealed that minimizing carbon source usage or boosting the substrate conversion rate can also improve the process economics. When the sucrose concentration was decreased from 20 to 16 g L^−1^, the breakeven point of PHB decreased by 7.8%. Leveraging the metabolic plasticity of microorganisms, the current substrate conversion rate (0.21g PHB/g sucrose) may be further improved by genetic engineering. With the conversion rate increased to 40–50.3% (theoretical conversion rate), WWTPs could achieve an economic return of 9.48 ∼19.78 $ per ton of wastewater treatment. Using a New Materials Technology Co., Ltd. in ZhenJiang as a case study, it could gain $12586.33∼26570.74 annually by upgrading its high‐concentration NMP wastewater (≈30 g L^−1^, 63.36 tons/year), thereby transforming the WWTPs from an energy‐intensive consumer to an economically viable producer. Considering full‐load operation of fermentation equipment and optimal resource allocation for industrial‐scale PHB production, the environmental sustainability and economic benefit of wastewater upgrading processes may be further improved.

## 3 Discussion and Conclusion

The conventional linear “take‐make‐dispose” paradigm has become incompatible with contemporary imperatives for sustainable wastewater management.^[^ ^53^ ^]^ Herein, we leveraged nitrogen‐cycling microorganisms as “sustainable cell factories” for wastewater upgrading, simultaneously achieving the wastewater depuration and high‐value PHA recovery. Compared to conventional wastewater‐derived products (e.g., biogas and phosphorus), PHA demonstrates unparalleled advantages in economic value and environmental benefits (Table S24, Supporting Information).^[^ ^54^ ^]^ For instance, the market values of PHA are 10–20 times higher than that of biogas produced from an equivalent amount of organic carbon. Correspondingly, many countries offer subsidies or enforce mandatory policies for PHA materials (e.g., Resolution to End Plastic Pollution (UNEA‐5/14) and USDA BioPreferred Program), whereas phosphorus recovery may face stricter regulatory constraints.^[^ ^55^, ^56^ ^]^ More encouragingly, PHA market is expected to expand from $93 million in 2022 to $195 million by 2028.^[^ ^1^ ^]^

Nevertheless, the traditional PHA synthesis processes require sterile conditions, as the nitrogen sources such as ammonium sulfate, yeast extract and corn steep liquor are typically broad‐spectrum and susceptible to microbial contamination.^[^ ^57^ ^]^ Although next‐generation industrial biotechnology (NGIB) based on extremophiles enables non‐sterile PHA synthesis, it introduces additional challenges (e.g., high‐salinity byproduct streams).^[^ ^58^ ^]^ Likewise, while promising for non‐sterile PHA production, conventional mixed microbial cultures (MMCs) face critical challenges in high‐toxicity nitrogenous streams, including structural instability, disrupted interspecies interactions, and metabolic dysfunction.^[^ ^14^, ^59^ ^]^ For the first time, we develop an innovative wastewater valorization technology using *Paracoccus* sp. ZQW‐1 to simultaneously achieve NMP wastewater depuration and non‐sterile PHB synthesis. Notably, *Paracoccus* sp. ZQW‐1 exhibits unparalleled bioremediation performance even at ultra‐high NMP concentrations (eliminating 15 g L^−1^ NMP within 108 h), surpassing previously documented functional strains. On the one hand, *Paracoccus* sp. ZQW‐1 could utilize recalcitrant NMP as the specific nitrogen source to synthesize PHB, thereby enhancing its resilience to adverse conditions. On the other hand, the inherent cytotoxicity and low bioavailability of NMP can effectively inhibit the proliferation of competing microorganisms. Capitalizing on these advantages, we realize non‐sterile PHB production (7.71 g L^−1^) and steady‐state wastewater purification in a 3‐L bioreactor. More encouragingly, this biorefinery strategy has been successfully applied to valorize diverse nitrogenous organic wastewaters (e.g., DMF, DMAC and PYD wastewater) by developing new “sustainable cell factories”.

Additionally, NMP wastewater‐driven PHB biorefinery processes significantly reduce the environmental impacts, especially GHG emission (8.8%). Considering the metabolic plasticity of microorganisms, this wastewater valorization strategy could achieve an economic return of 9.48 ∼19.78 $ per ton of wastewater once the substrate conversion rate reaches 0.4–0.5 g PHB/g sucrose. Therefore, future research should prioritize the development of engineered nitrogen‐cycling microorganisms with better PHB synthesis capacity and toxicity tolerance. Concurrently, it is essential to develop innovative process integration strategies and explore the valorization potential of by‐products to maximize economic and environmental benefits. Additionally, systematic scale‐up studies are required to bridge the gap between laboratory‐scale outcomes and real‐world implementation. Overall, this study offers a forward‐looking trajectory for converting refractory nitrogenous organic wastewaters to high‐value PHB products.

## 4 Experimental Section

### Chemicals and Industrial Wastewaters

Diverse nitrogenous organic wastewaters were collected from factories in Zhenjiang (NMP (C_5_H_9_NO)), Taixing (N, N‐dimethylformamide (DMF, C_3_H_7_NO)), Changde (Pyridine, (PYD, C_5_H_5_N)), and Zhumadian (N, N‐dimethylacetamide (DMAC, C_4_H_9_NO)), China. The detailed chemical reagents are summarized in Text S1 (Supporting Information).

### Development of Microbial Cell Factories

*Paracoccus* sp. ZQW‐1 can serve as an efficient microbial cell factory for NMP degradation and PHA production. Moreover, *Paracoccus* sp. ZJ‐J, *Paracoccus* sp. ZQW‐2, and *Paracoccus* sp. ZQW‐3 were isolated from DMF wastewater, PYD wastewater, and DMAC wastewater, respectively. The detailed screening and identification methods are listed in Text S2 (Supporting Information).

### Decontamination of NMP Wastewater

The details of NMP wastewater treatment are summarized in Text S3 (Supporting Information).

### PHA Production Using NMP as the Nitrogen Substrate

The PHA production was conducted using NMP as the specific nitrogen source in mineral salt medium (MSM), and the details are summarized in Text S4 (Supporting Information).

### Scalable PHA Production from Actual NMP Wastewater

The scalable PHA production was conducted in a 3‐L fermenter under non‐sterile conditions. The details are listed in Text S5 (Supporting Information).

### Soil Microcosm Experiments

Soil microcosm experiments were used to test the degradability of PHA materials, and the details are summarized in Text S6 (Supporting Information).

### Ecological Environmental Risk Assessment

The NMP‐derived intermediates were determined by liquid chromatography–mass spectrometer (LC–MS). The biotoxicity of intermediates was analyzed by Ecological Structure‐Activity Relationships (ECOSAR) program and systematic toxicity assessment tests. The details are listed in Texts S7−S9 (Supporting Information).

### Construction of Gene Deletion Mutants of *Paracoccus* sp. ZQW‐1

To investigate the causal link underlying the selectivity mechanism, a *nmpB* knockout mutant of *Paracoccus* sp. ZQW‐1 was successfully constructed and named *Paracoccus* sp. ZQW‐1‐Δ*nmpB*. The specific construction method is listed in Text S10 (Supporting Information). In addition, the strains, plasmids, and primers used for *Paracoccus* sp. ZQW‐1‐Δ*nmpB* construction are listed in Tables S25 and S26 (Supporting Information).

### Real‐Time Quantitative PCR Analysis

Total RNA was extracted using a high purity total RNA extraction kit (Proteinssci Biotech Co., Ltd.), which was subsequently reverse‐transcribed to cDNA using the HyperMB rapid reverse transcription kit (Sangon Biotech Co., Ltd.). The real‐time quantitative polymerase chain reaction (RT‐qPCR) experiments were performed on Applied Biosystems QuantStudio 1 Plus. The primer sequences are summarized in Table S27 (Supporting Information). In addition, the relative expression of functional genes was determined by the 2^–ΔΔCt^ method, and the 16S rRNA gene was used as an internal standard.^[^ ^60^, ^61^ ^]^

### Analytical Methods

The detailed detection methods for NMP, total organic carbon (TOC) and PHA are summarized in Text S11 (Supporting Information). In addition, PHA was characterized by transmission electron microscope (TEM, Hitachi HT‐7800), fourier transform infrared spectroscopy (FTIR, Thermo Scientific Nicolet iS20), gel permeation chromatography (GPC, Agilent 1260), ^1^H NMR spectrometer (Bruker Ascend TM 600MHz), differential scanning calorimetry (DSC, Netzsch DSC 200 F3), and thermal gravimetric analysis (TGA, Netzsch STA 449 F5). The whole genome of *Paracoccus* sp. ZQW‐1 was sequenced using Illumina sequencing platforms at the Shanghai Majorbio Bio‐pharm Technology Co., Ltd., and deposited in the National Center for Biotechnology Information (NCBI) under the accession number **PRJNA1265858**. The transcriptome data was also deposited in NCBI under the accession number **PRJNA1266529**.

### Environmental Life Cycle Assessment

Life‐cycle assessment was conducted according to the general methodological framework in ISO 14040 and 14044 (2006E) standards.^[^ ^62^ ^]^ The LCA models were established utilizing Simapro 9.5 software and Ecoinvent 3.9.1 database, and analyzed by ReCiPe methodology. Since LCA was conducted according to the lab data of PHB production utilizing NMP wastewater, the system boundaries were defined with the scope of cradle‐to‐gate, which excluded the utilities construction, downstream processing (upgrading PHB into final products), and end‐of‐life disposal to minimize the associated uncertainties.^[^ ^13^, ^63^ ^]^ Therefore, this study primarily focused on the technical advances of the constructed biorefinery process in sustainable wastewater management and non‐sterile PHB production. In addition, as the system function was to produce PHB from complex NMP wastewater, the functional unit was defined as 1 g of PHB, which was consistent with the common approach adopted in previous LCA research.^[^ ^38^, ^64^, ^65^, ^66^ ^]^

### Statistical Analysis

All degradation and fermentation tests were performed in at least triplicates to ensure data reliability. Results were expressed as the mean value ± standard deviation.

## Conflict of Interest

The authors declare no conflict of interest.

## Supporting information



Supporting Information

## Data Availability

The data that support the findings of this study are available from the corresponding author upon reasonable request.
