# Supplementary material for: Coupling Nitrogenous Organic Wastewater Treatment and Biorefinery via N‐Cycling Bacterium
Source: Adv Sci (Weinh). 2025 Nov 14;13(7):e13035. doi: 10.1002/advs.202513035 (PMC12866759; doi:10.1002/advs.202513035)
Supplement: Supplementary file 1 — Supporting Information [file ADVS-13-e13035-s001.docx]

**Coupling Nitrogenous Organic Wastewater Treatment and Biorefinery via N-cycling bacterium**

*Ziqian Wang, Chunyu Du, Guanyu Zheng*, Dahu Ding**

College of Resources and Environmental Sciences, Nanjing Agricultural University, Nanjing 210095, China

**** Corresponding authors (G. Zheng & D. Ding)***

***E-mail: gyzheng@njau.edu.cn & ddh@njau.edu.cn***

***The Supplementary Material includes 11 Texts, 27 Tables, and 66 Figures.***

**Table of Contents**

**Supplementary Methods**

**Text S1.** The details of chemicals and reagents **9**

**Text S2.** The details of *Paracoccus* sp. ZQW-1 isolation and identification **11**

**Text S3.** The details of NMP degradation experiments **12**

**Text S4.** The production of PHA by using NMP as the specific nitrogen substrate **13**

**Text S5.** Semi-continuous and continuous PHA production from actual NMP wastewater **15**

**Text S6.** The details of soil microcosm experiments **16**

**Text S7.** The details of *E. coli* growth assessment experiments **17**

**Text S8.** The details of *Chlorella vulgaris* growth assessment experiments **18**

**Text S9.** The details of soybean seed germination experiments **19**

**Text S10.** Construction of gene deletion mutants of *Paracoccus* sp. ZQW-1 **20**

**Text S11.** The details of analytical methods **21**

**Supplementary Figures**

**Fig. S1.** The SEM image of *Paracoccus* sp. ZQW-1 **22**

**Fig. S2.** The average nucleotide identity (ANI) between *Paracoccus* sp. ZQW-1 and other *Paracoccus* strains **23**

**Fig. S3.** The cultivation of *Paracoccus* sp. ZQW-1 in LB medium under different conditions **24**

**Fig. S4.** Biodegradation of NMP by *Paracoccus* sp. ZQW-1 **25**

**Fig. S5.** The effects of initial concentration on NMP degradation **26**

**Fig. S6.** The NMP degradation under different environmental conditions **27**

**Fig. S7.** The degradation of actual NMP wastewater by *Paracoccus* sp. ZQW-1 **28**

**Fig. S8.** The degradation of actual NMP wastewater with ultra-high concentrations by *Paracoccus* sp. ZQW-1 **29**

**Fig. S9.** HPLC-MS spectra of NMP and its intermediates **30**

**Fig. S10.** The genome and plasmid circular atlas of *Paracoccus* sp. ZQW-1 **31**

**Fig. S11.** The volcano plot of differential genes under different groups (i.e., *Paracoccus* sp. ZQW-1 cultivation and NMP degradation process) **32**

**Fig. S12.** The toxic evolution of NMP wastewater evaluated by *Chlorella vulgaris* **33**

**Fig. S13.** Microscopic observation of *Chlorella vulgaris* growth under various culture conditions **34**

**Fig. S14.** The phenotype of soybean seeds cultured by pure water, NMP solution, and the treated NMP solution **35**

**Fig. S15.** The functional annotations of *Paracoccus* sp. ZQW-1 genome against the Gene Ontology categories (GO) database **36**

**Fig. S16.** The Kyoto Encyclopedia of Genes and Genomes database (KEGG) categories of microbial metabolism of *Paracoccus* sp. ZQW-1 **37**

**Fig. S17.** The accumulation of PHB during NMP degradation process **38**

**Fig. S18.** Identification of PHA synthesis function of *Paracoccus* sp. ZQW-1 **39**

**Fig. S19.** The effects of sucrose concentration (5−25 g L^−1^) and C/N ratio (5−100) on PHB synthesis **40**

**Fig. S20.** The effects of glucose concentration (5−25 g L^−1^) and C/N ratio (5−100) on PHB synthesis **41**

**Fig. S21.** The effects of fructose concentration (5−25 g L^−1^) and C/N ratio (5−100) on PHB synthesis **42**

**Fig. S22.** The effects of glycerin concentration (5−25 g L^−1^) and C/N ratio (5−100) on PHB synthesis **43**

**Fig. S23.** The effects of fermentation conditions on PHB synthesis **44**

**Fig. S24.** The characterizations of intracellular PHB granules by TEM **45**

**Fig. S25.** The comparison of PHA synthesis by *Paracoccus* sp. ZQW-1 and other documented *Paracoccus* strains **46**

**Fig. S26.** The comparison of PHB production by *Paracoccus* sp. ZQW-1 using synthetic and actual NMP wastewater as nitrogen sources under optimal fermentation conditions **47**

**Fig. S27.** Continuous production of PHB and degradation of NMP under selective pressure **48**

**Fig. S28.** The principal component analysis (PCA) of *Paracoccus* sp. ZQW-1 under different groups (i.e., *Paracoccus* sp. ZQW-1 cultivation, NMP degradation stage, and PHB synthesis stage) **49**

**Fig. S29.** The Venn diagram of transcriptome regulation of *Paracoccus* sp. ZQW-1 under different groups (i.e., *Paracoccus* sp. ZQW-1 cultivation, NMP degradation, and PHB synthesis process (PHB accumulation stage, Day 2)) to illustrate the overlap of functional genes **50**

**Fig. S30.** The Venn diagram of transcriptome regulation of *Paracoccus* sp. ZQW-1 under different groups (i.e., *Paracoccus* sp. ZQW-1 cultivation, NMP degradation, and PHB synthesis process (PHB consumption stage, Day 4)) to illustrate the overlap of functional genes **51**

**Fig. S31.** The volcano plot of differential genes under different groups (i.e., *Paracoccus* sp. ZQW-1 cultivation and PHB synthesis process (PHB accumulation stage, Day 2)) **52**

**Fig. S32.** The volcano plot of differential genes under different groups (i.e., *Paracoccus* sp. ZQW-1 cultivation and PHB synthesis process (PHB consumption stage, Day 4)) **53**

**Fig. S33.** The expression levels of genes responsible for PHA synthesis under different groups (i.e., *Paracoccus* sp. ZQW-1 cultivation and NMP degradation stage) by transcript per million (TPM) method **54**

**Fig. S34.** The expression levels of *phaA*, *phaB*, and *phaC* under different groups (i.e., NMP degradation stage and PHB synthesis stage (Day 2)) obtained by transcriptome analysis and RT-qPCR **55**

**Fig. S35.** The expression levels of NMP-degrading genes under different groups (i.e., *Paracoccus* sp. ZQW-1 cultivation and PHB synthesis process (PHB accumulation stage, Day 2)) by transcript per million (TPM) method **56**

**Fig. S36.** The expression levels of NMP-degrading genes under different groups (i.e., *Paracoccus* sp. ZQW-1 cultivation and PHB synthesis process (PHB consumption stage, Day 4)) by transcript per million (TPM) method **57**

**Fig. S37.** The comparison of PHB production by *Paracoccus* sp. ZQW-1 and *Paracoccus* sp. ZQW-1-*ΔnmpB* under different nitrogen sources **58**

**Fig. S38.** The NMP degradation and PHB synthesis capabilities of *Paracoccus* sp. ZQW-1-*ΔnmpB* **59**

**Fig. S39.** The PHB production by co-culture of *Paracoccus* sp. ZQW-1 and representative microbial competitors (i.e., *B. subtilis* 168 and *E. coli* MG1655) using different nitrogen sources **60**

**Fig. S40.** Elucidating the microbial community dynamics in NMP-driven PHB fermentation systems by metagenomic analyses **61**

**Fig. S41.** The growth of *Paracoccus* sp. ZQW-1 and *Paracoccus* sp. ZQW-1-*ΔnmpB* in LB medium **62**

**Fig. S42.** The picture of semi-continuous PHB production (1-L scale) using simulated NMP wastewater as the nitrogen source under non-sterile conditions **63**

**Fig. S43.** The non-sterile PHB production in a 1-L bioreactor by controlling the feeding amount of NMP **64**

**Fig. S44.** The non-sterile PHB production in a 1-L bioreactor by controlling the feeding amount of (NH_4_)_2_SO_4_ **65**

**Fig. S45.** The non-sterile PHB production in a 1-L bioreactor by controlling the feeding amount of (NH_4_)_2_SO_4_ **66**

**Fig. S46.** The microbial community dynamics of NMP and (NH_4_)_2_SO_4_-driven PHB fermentation systems under non-sterile conditions **67**

**Fig. S47.** The collected NMP wastewater and the filtered water sample **68**

**Fig. S48.** The semi-continuous PHB production from actual NMP wastewater in a 1-L bioreactor by controlling the feeding amount of NMP **69**

**Fig. S49.** The continuous PHB production from filtered industrial NMP wastewater using *Paracoccus* sp. ZQW-1 as a “sustainable cell factory” under non-sterile conditions **70**

**Fig. S50.** The semi-continuous PHB production by *Paracoccus* sp. ZQW-2 using simulated PYD wastewater and (NH_4_)_2_SO_4_ as nitrogen sources under non-sterile conditions **71**

**Fig. S51.** The continuous PHB production from actual PYD wastewater by *Paracoccus* sp. ZQW-2 under non-sterile conditions **72**

**Fig. S52.** The extracted PHB from *Paracoccus* sp. ZQW-1 **73**

**Fig. S53.** The comparison between standard PHB and wastewater-derived PHB by ^1^H-NMR spectra **74**

**Fig. S54.** The comparison between standard PHB and wastewater-derived PHB by FT-IR spectrum, DSC analysis, and TGA analysis **75**

**Fig. S55.** The molecular weight distribution of wastewater-derived PHB and the comparison with other documented PHB polymers **76**

**Fig. S56.** The degradation of PHB film and polystyrene (PS) film in soil and the morphological changes 7**7**

**Fig. S57.** The system boundary of conventional PHB production under sterile conditions **78**

**Fig. S58.** The system boundary of wastewater-driven PHB production under non-sterile conditions **79**

**Fig. S59.** The sankey flow diagram of 18 midpoint indicators to show their contribution for PHB production **80**

**Fig. S60.** The sankey flow diagram of the contribution of each production step for global warming **81**

**Fig. S61.** The sankey flow diagram of the contribution of each production step for terrestrial ecotoxicity **82**

**Fig. S62.** The sankey flow diagram of the contribution of each production step for fossil resource scarcity **83**

**Fig. S63.** The sankey flow diagram of the contribution of each production step for fine particulate matter formation, freshwater ecotoxicity, freshwater eutrophication, and human carcinogenic toxicity **84**

**Fig. S64.** The sankey flow diagram of the contribution of each production step for ionizing radiation, land use, marine ecotoxicity, and marine eutrophication **85**

**Fig. S65.** The sankey flow diagram of the contribution of each production step for mineral resource scarcity, ozone formation, human health, ozone formation, terrestrial ecosystems, stratospheric ozone depletion **86**

**Fig. S66.** The sankey flow diagram of the contribution of each production step for terrestrial acidification and water consumption **87**

**Supplementary Tables**

**Table S1.** The basic information of NMP wastewater collected from a New Materials Technology Co., Ltd. in ZhenJiang, China **88**

**Table S2.** The biodegradation of NMP by *Paracoccus* sp. ZQW-1 and other documented bacterial strains **89**

**Table S3.** The functional genes related to NMP biodegradation **90**

**Table S4.** The production of PHA by different carbon sources **91**

**Table S5.** The optimized PHB production by using sucrose as the carbon source **92**

**Table S6.** The effects of fermentation time on PHB production **94**

**Table S7.** The effects of inoculation dosage on PHB production **95**

**Table S8.** The effects of solution pH on PHB production **96**

**Table S9.** The effects of NaCl concentration on PHB production **97**

**Table S10.** The effects of fermentation temperature on PHB production **98**

**Table S11.** The effects of nitrogen sources on PHB production **99**

**Table S12.** The comparison of PHA production by *Paracoccus* sp. ZQW-1 with other documented *Paracoccus* sp. strains **100**

**Table S13.** The genes in *Paracoccus* sp. ZQW-1 related to PHB metabolism **101**

**Table S14.** The mechanical properties of PHB produced by *Paracoccus* sp. ZQW-1 **102**

**Table S15.** The molecular weight of PHB produced by *Paracoccus* sp. ZQW-1 and other documented strains **103**

**Table S16.** The environmental impacts resulting from the enrichment and culture of *Paracoccus* sp. ZQW-1 (1 cm^3^) **105**

**Table S17.** The environmental impacts resulting from the production of 1 cm^3^ PHB fermentation liquid using (NH_4_)_2_SO_4_ as the nitrogen source **106**

**Table S18.** The environmental impacts resulting from the production of 1 cm^3^ PHB fermentation liquid using NMP as specific nitrogen source **107**

**Table S19.** The environmental impacts resulting from the enrichment of 1 g dried cells using (NH_4_)_2_SO_4_ as the nitrogen source **108**

**Table S20.** The environmental impacts resulting from the enrichment of 1 g dried cells using NMP as specific nitrogen source **109**

**Table S21.** The environmental impacts of PHB production using (NH_4_)_2_SO_4_ as the nitrogen source **110**

**Table S22.** The environmental impacts of PHB production using NMP as specific nitrogen source **111**

**Table S23.** The price of chemicals used for techno-economic assessment. **112**

**Table S24.** Representative and marketable high-value commodities recovered from WWTPs. **118**

**Table S25.** The strains and plasmids used for *nmpB* gene knockout **120**

**Table S26.** The primers used for *nmpB* gene knockout. **121**

**Table S27.** The primers used in RT-qPCR experiments. **122**

**Supplementary Methods.**

**Text S1. The details of chemicals and reagents.**

***The chemical reagents for strain screening:***

Nile Red (≥95%) and Sudan Black reagents were purchased from Aladdin, China.

***The chemical reagents for strain culture:***

Yeast extract (OXOID) and Tryptone (OXOID) were purchased from Nanjing Huiya Biotechnology Co., Ltd (Nanjing, China). NaCl (≥99.5%) was obtained from Shanghai Hushi Chemical Co., Ltd (Shanghai, China). Ultrapure water was obtained from the water purification system (EPED-10TF).

***The chemical reagents for PHA production:***

Butyric acid (>99.5%), Propionic acid (>99%), and Lactose (BR) were purchased from Aladdin, China. Valeric acid (>99%) was purchased from Nanjing wanqing chemical glass ware & instrument Co., Ltd (Nanjing, China). NMP (>99%), Acetic acid (>99.7%), Fructose (>99%), Maltose (>95%), Xylose (>99%), Galactose (>99%), Mannitol (>99%), Cellulose, Lignin, (NH_4_)_2_SO_4_ (>99%), NH_4_Cl (>99.8%), NaNO_2_ (>99%), and CH_4_N_2_O (>99%) were purchased from Macklin, China. Glucose (>99%) and Sucrose (>99.5%) were purchased from Sinopharm Chemical Reagent Co., Ltd (Shanghai, China). Glycerin (>99%) and Sodium citrate (BR) were obtained from Shanghai Hushi Chemical Co., Ltd (Shanghai, China). Ethanol (≥99.8%), Methanol (≥99.9%), and Soluble starch were purchased from Nanjing chemical reagent Co., Ltd (Nanjing, China).

***The chemical reagents for PHA extraction:***

Trichloromethane (≥99.8%) was purchased from Yonghua Chemical Co., Ltd (Changzhou, China). Sulfuric acid (GR) was purchased from Nanjing chemical reagent Co., Ltd (Nanjing, China). Methyl benzoate (≥98%) was purchased from Macklin, China.

**Text S2. The details of *Paracoccus* sp. ZQW-1 isolation and identification.**

***The collection of actual NMP wastewater:***

The actual NMP wastewater was collected from a New Materials Technology Co., Ltd. in ZhenJiang, Jiangsu Province.

***The isolation of Paracoccus sp. ZQW-1:***

One milliliter of wastewater sample was added to the mineral salt medium (MSM) containing 1 g L^−1^ NMP to enrich the NMP degrading strains. After that, the isolated NMP degrading strains were plated on MSM solid medium containing 1.0 mg L^−1^ Nile Red dye and 10 g L^−1^ glucose, and incubated upside down for 72 h. The single colony that displayed red fluorescence under 365 nm UV light was transferred to 40 % glycerol tubes and stored at −80 °C. In addition, the pure colony was stained with Sudan black dye and observed under a microscope (Nikon, MQD42055).

***The identification of Paracoccus sp. ZQW-1:***

For the screened PHA-producing strain, genus identification was conducted using 16S rRNA gene sequencing, whole-genome sequencing, and phylogenetic tree analysis. The 16S rRNA sequencing was provided by Sangon Biotech (Shanghai) Co., Ltd. and the whole-genome sequencing was carried out and analyzed by Shanghai Majorbio Bio-pharm Technology Co., Ltd. In addition, the phylogenetic tree was constructed using MEGA 11.0 software.

**Text S3. The details of NMP degradation experiments.**

The NMP degradation was carried out in the mineral salt medium (MSM). The effects of various parameters (e.g., pH value, temperature, NaCl concentration, and initial NMP concentration) on NMP degradation were explored. Moreover, the treatment of actual NMP wastewater by *Paracoccus* sp. ZQW-1 was investigated.

***The preparation of MSM medium:***

The MSM medium consisted of 9 g L^−1^ Na_2_HPO_4_•12H_2_O, 1.5 g L^−1^ KH_2_PO_4_, 0.2 g L^−1^ MgSO_4_•7H_2_O, 0.3 g L^−1^ H_3_BO_3_, 0.2 g L^−1^ CoCl_2_•6H_2_O, 0.1 g L^−1^ ZnSO_4_•7H_2_O, 0.03 g L^−1^ MnSO_4_•5H_2_O, 0.03 g L^−1^ (NH_4_)_6_Mo_7_O_24_•4H_2_O, 0.02 g L^−1^ NiCl_2_•6H_2_O, and 0.01 g L^−1^ CuSO_4_•5H_2_O.

***The preparation of Luria-Bertani (LB) medium and LB agar plate:***

The LB medium consisted of 5 g L^−1^ Yeast extract, 10 g L^−1^ Tryptone, and 5 g L^−1^ NaCl. Moreover, 1.5% agar powder was added into the LB medium to prepare the LB agar plate.

***The NMP degradation:***

First, *Paracoccus* sp. ZQW-1 was inoculated from the freshly streaked LB agar plates into 100 mL of LB medium. After that, the LB medium was shaken at 200 rpm for 18~24 h at 30 °C. Then, 100 μL of the cultured solution was added into 100 mL of LB medium, and shaken for 18~24 h. Moreover, the bacterial solution was centrifuged at 8000 rpm for 5 minutes to remove the LB medium and resuspended in MSM medium. The MSM medium with NMP were used to determine the degradation efficiency and the inoculation concentration was 4%.

**Text S4. The production of PHA by using NMP as the specific nitrogen substrate.**

The PHA production was conducted in 100 mL conical flasks. Different fermentation parameters such as carbon source, NaCl concentration, fermentation time, and inoculation dosage were investigated to optimize PHA production.

***Preparation of seed solution:***

Likewise, *Paracoccus* sp. ZQW-1 was inoculated from the freshly streaked LB agar plates into 100 mL of LB medium. After that, the obtained bacterial solution was centrifuged at 8000 rpm for 5 minutes to remove the LB broth and resuspended in MSM medium to obtain the seed solution.

***The production of PHA:***

The production of PHA was first conducted in the MSM medium with 10 g L^−1^ carbon sources (i.e., glucose, fructose, sucrose, maltose, lactose, xylose, galactose, sodium citrate, cellulose, lignin, glycerin, butyric acid, acetic acid, propionic acid, valeric acid, mannitol, ethanol, methanol, soluble starch) and the C/N (provided from NMP) ratio was 40:1. Furthermore, different cultural conditions, such as substrate concentration (from 5 to 25 g L^−1^), nitrogen sources (e.g., NMP, urea, ammonium sulfate, ammonium chloride, sodium nitrite, and sodium nitrite), solution pH (5−11), NaCl concentration (0−70 g L^−1^), inoculation dosage (2%−14 %), and C/N ratios (5−100), were explored to optimize the fermentation process.

***Co-culture competition analysis:***

*Bacillus subtilis* 168 and *Escherichia coli* MG1655 were used as the microbial competitors for co-culture experiments. First, these microbial competitors were cultured in LB medium to obtain the seed solution. After that, the cell pellets were washed with MSM medium to remove the nutrient residues. Then, *Paracoccus* sp. ZQW-1 and the corresponding competitors were inoculated into the fermentation medium at 4 % and 0.4 % inoculation concentrations, respectively. After fermentation, the abundances of *Paracoccus* sp. ZQW-1 and corresponding microbial competitors were determined by plate counting method. In addition, a more rigorous challenge experiment was conducted by introducing a small percentage (0.4% and 8%) of activated sludge into the fermentation system. The basic characteristics of the sludge were as follows: total solids = 6.72±0.3 g L^−1^, pH = 7.2±0.04, COD = 59.2±5.6 mg L^−1^, NH_4_^+^-N = 1.9±0.3 mg L^−1^, and total phosphorus = 0.17±0.03 mg L^−1^.

***The PHA production by using other nitrogenous organic compounds as specific nitrogen sources:***

*Paracoccus* sp. ZJ-J could use DMF as the specific nitrogen source to accumulate PHB ([Organics] = 10 g L^−1^, [C/N ratio] = 40: 1, [pH] = 7, [temperature] = 30 °C, [fermentation time] = 3 d).

*Paracoccus* sp. ZQW-2 could use PYD as the specific nitrogen source to accumulate PHB ([Organics] = 5 g L^−1^, [C/N ratio] = 60: 1, [pH] = 7, [temperature] = 30 °C, [fermentation time] = 3 d).

*Paracoccus* sp. ZQW-3 could use DMAC as the specific nitrogen source to accumulate PHB ([Organics] = 5 g L^−1^, [C/N ratio] = 60: 1, [pH] = 7, [temperature] = 30 °C, [fermentation time] = 3 d).

**Text S5. Semi-continuous and continuous PHA production from actual NMP wastewater.**

***The semi-continuous PHA production in a 1-L fermenter:***

The fermentation temperature was maintained at a constant temperature (30 ± 1 °C) through a water bath, and the dissolved oxygen was provided by the oxygen pump. The NMP stock solutions with different concentration (i.e., 0, 1, 3, 6, 9, and 12 g L^−1^) were prepared for nitrogen feeding, and the flow rate was controlled at 2 mL h^−1^ through a peristaltic pump. In addition, 20 g L^−1^ sucrose was added as the carbon source.

***The continuous PHA production in a 3-L fermenter:***

The initial fermentation broth consisted of 2-L MSM medium and 20 g L^−1^ sucrose. The actual wastewater containing 5 g L^−1^ NMP was used to provide the nitrogen source for PHA production, and the daily intake was 120 mL. Replenished the sucrose once its concentration fell below 5 g L^−1^. At predetermined times, samples were taken to monitor PHA production and NMP removal.

**Text S6. The details of soil microcosm experiments.**

The soil was collected from the garden of Nanjing agriculture university (32°03′ N, 118°46′ E). The collected soil was poured into containers with dimensions of 8 cm × 8 cm × 8 cm (W × L × H). For the degradation experiment, 10 similar setups were created and the pre-weighed PHA films (3 cm×5 cm, W × L) were buried in the soil, which were further incubated at room temperature (25 °C) and under simulated sunlight to mimic the natural conditions. In particular, polystyrene was used as a control. At pre-determined intervals, PHA films were removed from the respective containers. The extracted PHA films were washed with 70% ethanol and then with distilled water to remove the soil particles, bacterial biofilms, and other impurities. Finally, the washed PHA films were dried and weighed to determine the degradation rate.

**Text S7. The details of *E. coli* growth assessment experiments.**

*E. coli* (as a typical prokaryote) was cultured in different systems to evaluate the ecological environmental risk of this integrated technology. First, *E. coli* was inoculated into LB medium and incubated at 37°C and 200 rpm for 24 hours to ensure adequate bacterial growth. Subsequently, 1 mL of the bacterial solution was inoculated into liquid medium prepared with pure water, NMP solution (1.44 g L^−1^) and treated NMP solution. After cultivation (24 hours), 100 μL of the bacterial solution was plated on the LB agar plate. After 24 hours of incubation at 37°C, the number of colonies was obtained by colony counting.

**Text S8. The details of** ***Chlorella vulgaris* growth assessment experiments.**

*Chlorella vulgaris* (as a typical eukaryote) was inoculated into BG11 culture medium prepared with pure water, NMP solution (1.44 g L^−1^) and treated NMP solution. Various indicators such as *Chlorella vulgaris* concentration (OD_750 nm_), chlorophyll a (Chla), chlorophyll b (Chlb), and carotenoids (Car) were measured to determine the toxicity of different systems. The contents of Chla, Chlb and Car were calculated by the following equation:

*Chla (mg L^−1^) = (15.65 * OD_666_ - 7.34 * OD_653_) (1)*

*Chlb (mg L^−1^) = (27.05 * OD_653_ - 11.21 * OD_666_) (2)*

*Car (mg L^−1^) = (1000 * OD_470_ - 2.86 * Chla - 12.92 * Chlb)/245 (3)*

**Text S9. The details of soybean seed germination experiments.**

For the experimental setup, nine culture dishes, each with a diameter of 9 cm, were prepared. One piece of filter paper was placed inside each dish to support the soybean seeds. Thirteen seeds were neatly arranged in each dish by using tweezers. Subsequently, 10 mL of NMP stock solution and the treated NMP solution were added to the respective dishes, respectively. For the control group, an equal amount of pure water was added instead of the reaction solution. Each treatment was replicated three times to ensure the reliability of the results. During the cultivation period, the culture dishes were placed in an incubator at a constant temperature of 25°C.

**Text S10. Construction of gene deletion mutants of *Paracoccus* sp. ZQW-1**

The deletion of the *nmpB* gene from *Paracoccus* sp. ZQW-1 was performed using a two-step homologous recombination method with the suicide plasmid pJQ200SK.^[^[^1-3^](#_ENREF_1)^]^ Two homologous recombination-directing sequences were amplified using primers *nmpB*-pJQ200SK-F and *nmpB*-pJQ200SK-R, respectively. The two PCR products were then connected together and ligated into SacI/PstI-digested pJQ200SK using the ClonExpress MultiS One Step cloning kit (Vazyme Biotech, Nanjing, China) to construct the knockout plasmid *nmpB*-pJQ200SK. After that, the *nmpB*-pJQ200SK was introduced into *Paracoccus* sp. ZQW-1 cells by triparental conjugative transfer with pRK600 as the helper. Subsequently, the single crossover mutants were screened by dilution coating cells on an LB plate supplemented with streptomycin (50 mg L^–1^) and gentamicin (50 mg L^–1^). The correct single-crossover mutants were then subjected to repeated cultivation in LB medium supplemented with 10% sucrose. Finally, the deletion of the *nmpB* gene was definitively confirmed by PCR.

**Text S11. The details of analytical methods.**

***The details of NMP determination:***

The concentration of NMP was determined by high-performance liquid chromatography (HPLC, FL 5090). More specifically, the mobile phase consisted of methanol and water (20: 80, V: V). The flow rate was 1.0 ml min^−1^, and the detection wavelength was 214 nm.

***The details of TOC determination:***

The TOC elimination was measured by TOC analyzer (vario TOC select).

***The details of PHA extraction and determination:***

First, the dried cell samples (10 mg) were added into the mixture of methanol (1 mL, containing 15% sulfuric acid) and chloroform (1 mL). After that, the mixture was transformed to an oven and reacted at 100 ℃ for 4 h to extract the intracellular PHA. Subsequently, 1 mL of deionized water and 1 mL of chloroform (containing 0.05 % methyl benzoate) were added into the mixture to achieve the organic phase stratification. Finally, the subnatant was extracted, filtered through the organic filter membrane (0.22 μm), and analyzed by gas chromatography (GC9790 Plus).

**Supplementary Figures**

**
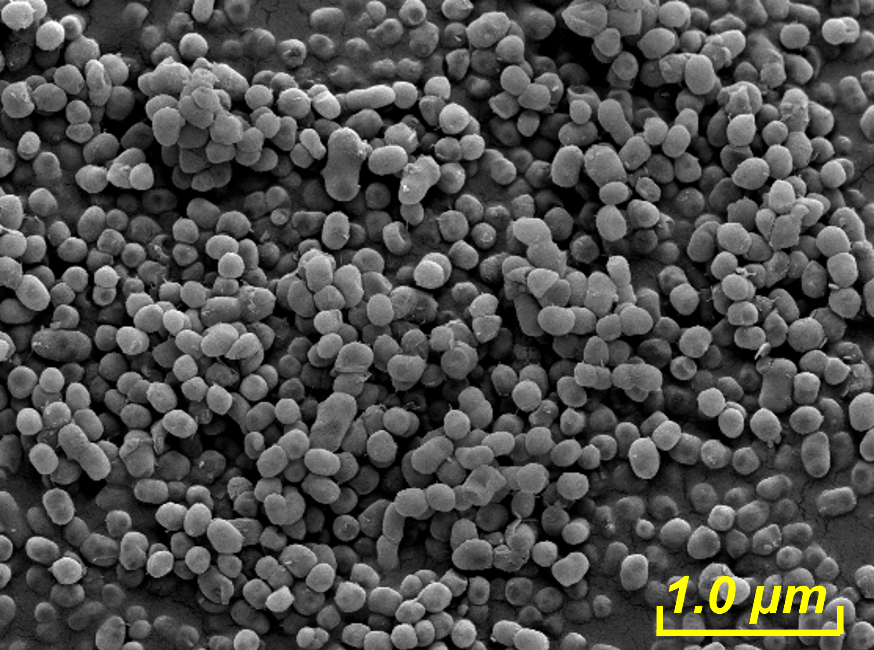
**

**Supplementary Fig. S1.** The SEM image of *Paracoccus* sp. ZQW-1.


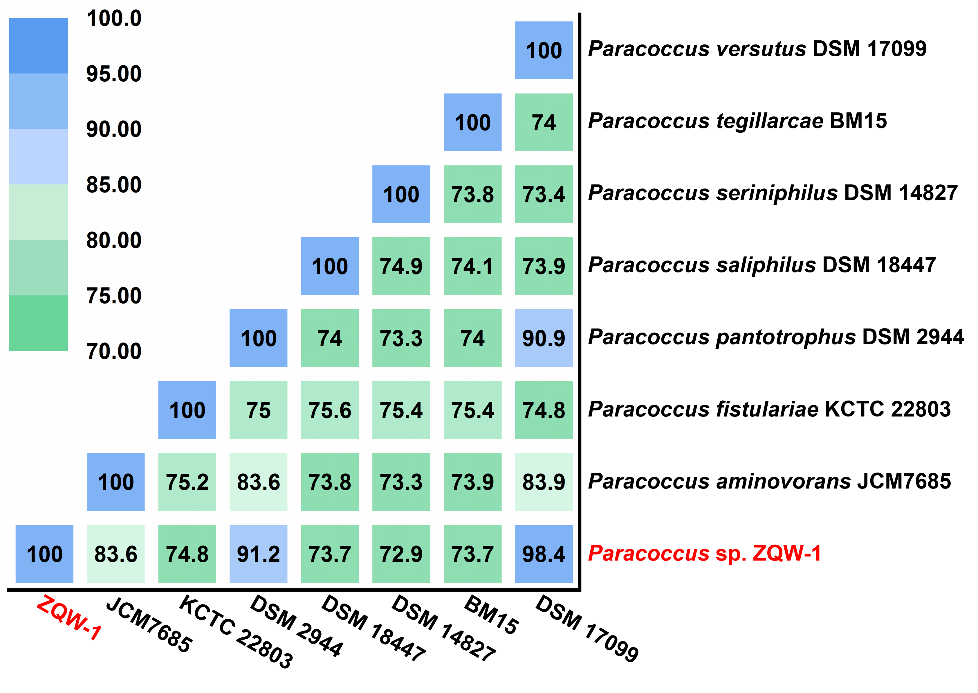


**Supplementary Fig. S2.** The average nucleotide identity (ANI) between *Paracoccus* sp. ZQW-1 and other *Paracoccus* strains.


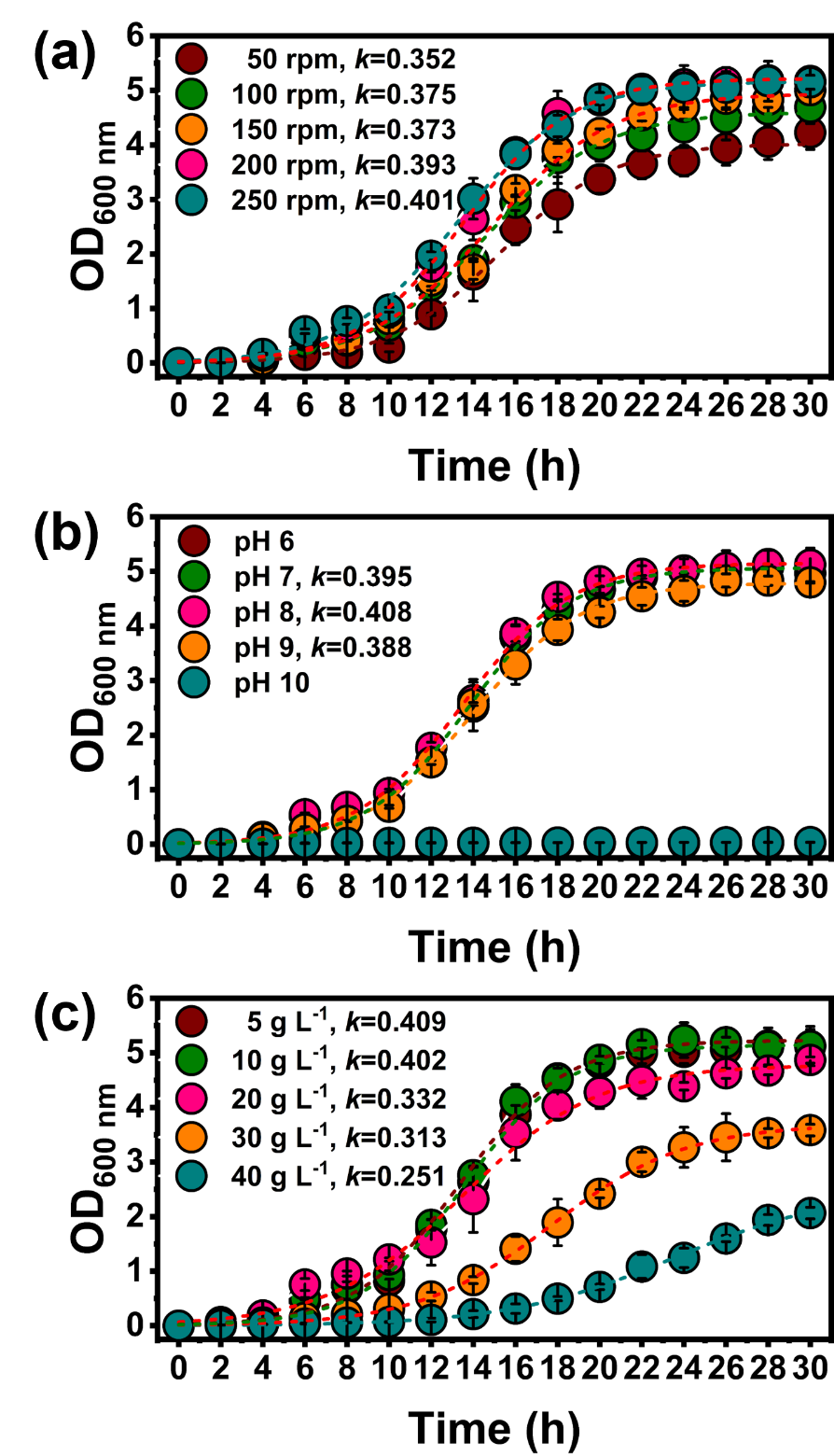


**Supplementary Fig. S3.** The cultivation of *Paracoccus* sp. ZQW-1 in LB medium under different conditions. **a**, 50−250 rpm. **b,** pH 6−10. **c,** 5−40 g L^−1^ NaCl.

**
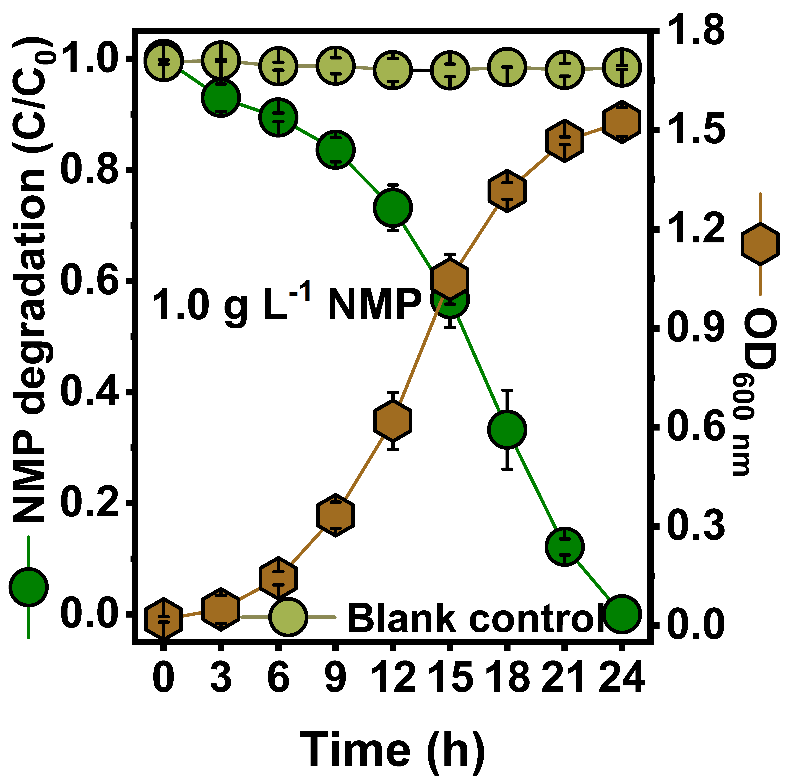
**

**Supplementary Fig. S4.** Biodegradation of NMP by *Paracoccus* sp. ZQW-1. ([NMP] = 1 g L^−1^, [inoculum concentration] = 4%, [pH] = 7, [temperature] = 30 °C).

**
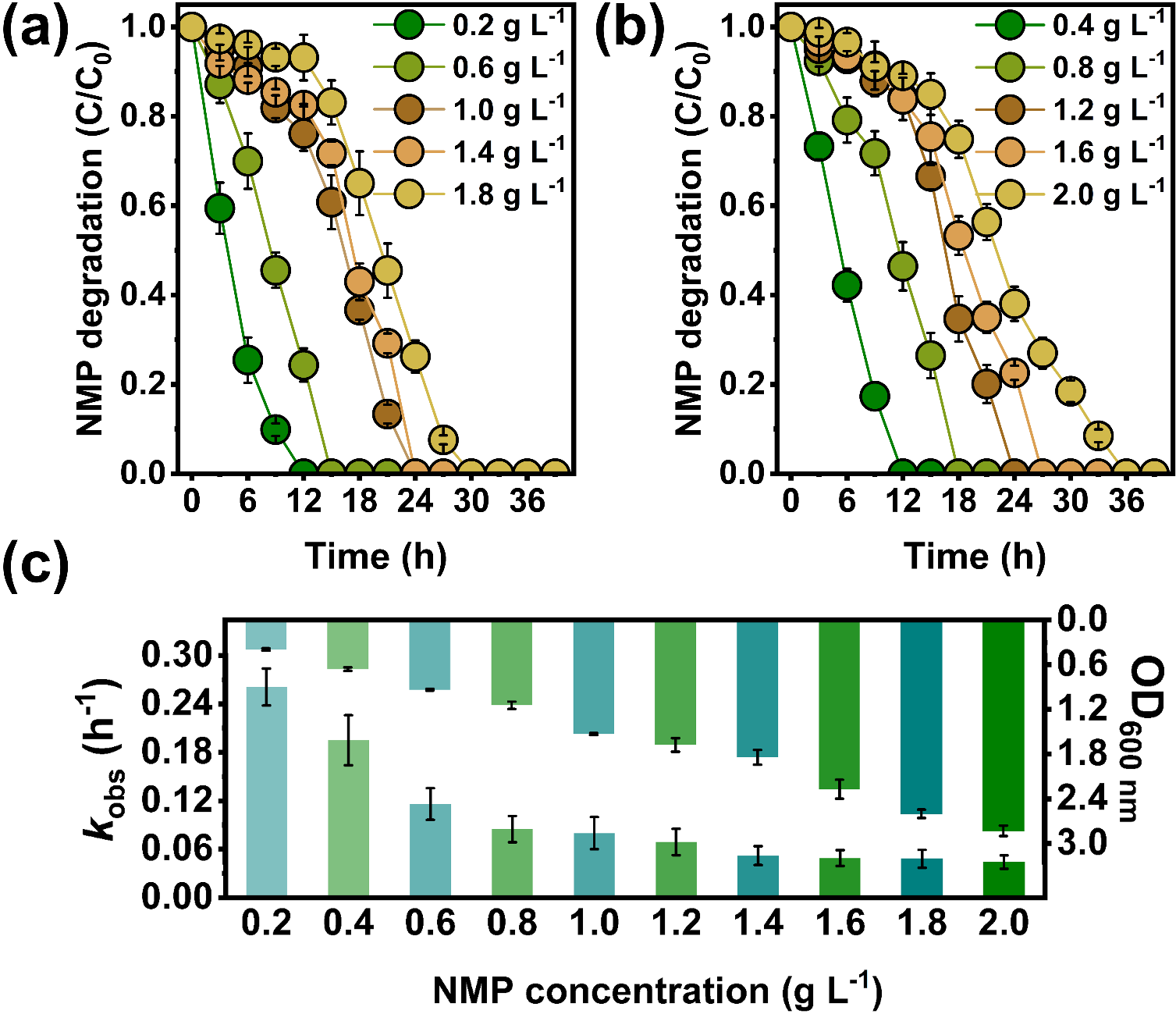
**

**Supplementary Fig. S5.** The effects of initial concentration on NMP degradation. **a, b,** The NMP degradation performance. **c,** The corresponding rate constant and OD_600 nm_. ([NMP] = 0.2−2 g L^−1^, [inoculum concentration] = 4%, [pH] = 7, [temperature] = 30 °C).


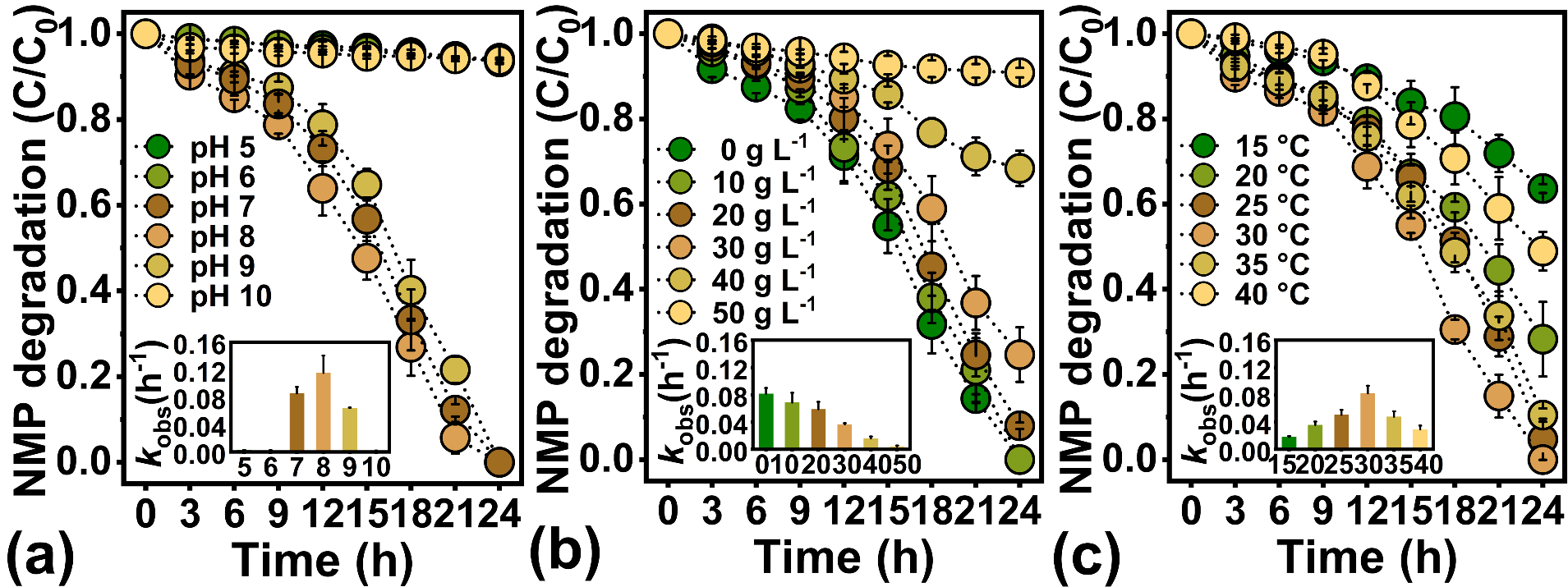


**Supplementary Fig. S6.** The NMP degradation under different environmental conditions. **a,** solution pH. **b,** NaCl concentrations. **c**, temperature. ([NMP] = 1 g L^−1^, [inoculum concentration] = 4%, [pH] = 5−10, [NaCl] = 0−50 g L^−1^, [temperature] = 15−40°C).


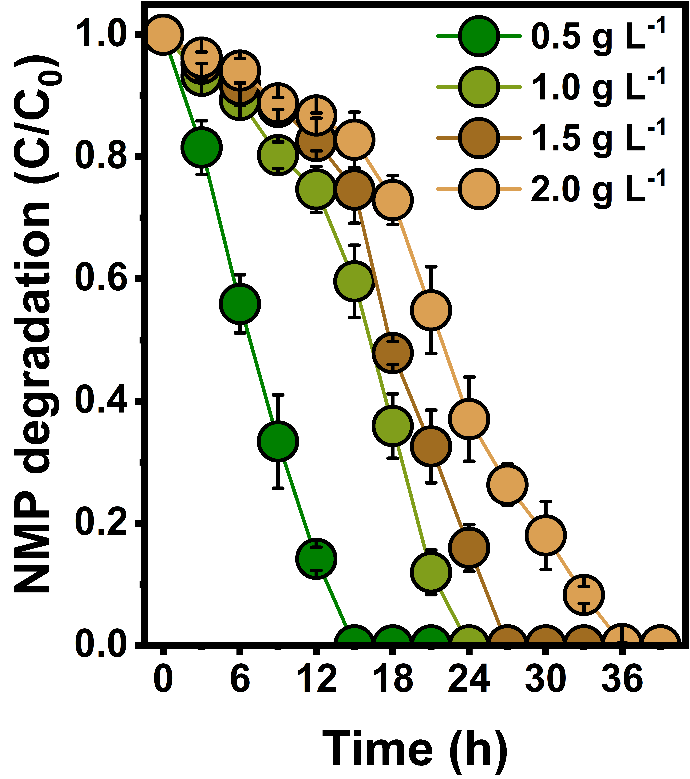


**Supplementary Fig. S7.** The degradation of actual NMP wastewater by *Paracoccus* sp. ZQW-1. ([NMP] = 0.5−2 g L^−1^, [inoculum concentration] = 4%, [pH] = 7, [temperature] = 30 °C).


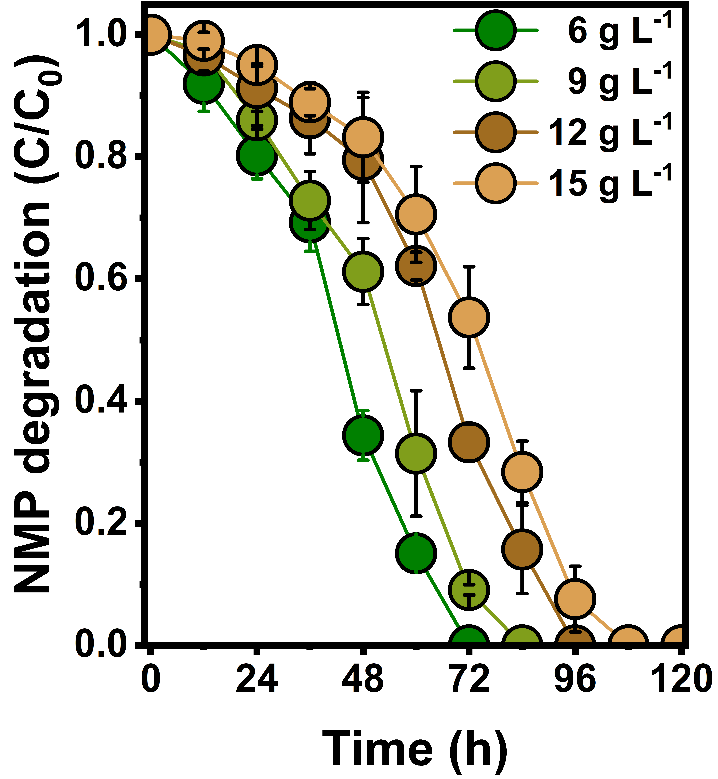


**Supplementary Fig. S8.** The degradation of actual NMP wastewater with ultra-high concentrations by *Paracoccus* sp. ZQW-1. ([NMP] = 6−15 g L^−1^, [inoculum concentration] = 4%, [pH] = 7, [temperature] = 30 °C).


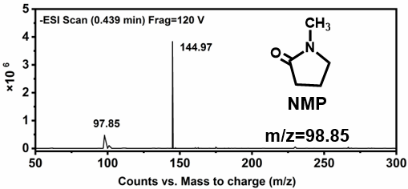


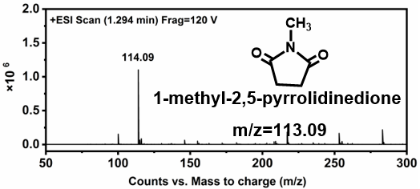

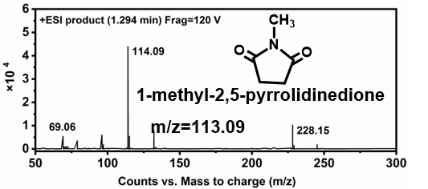


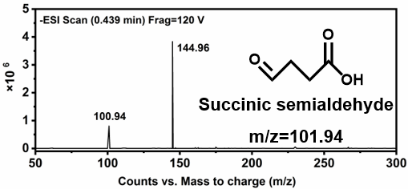

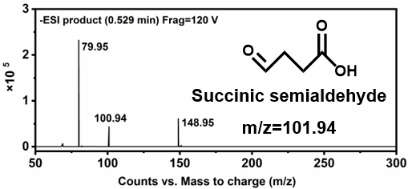


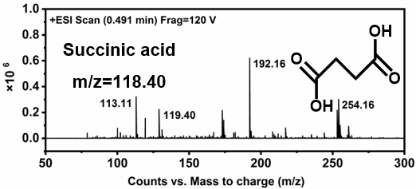

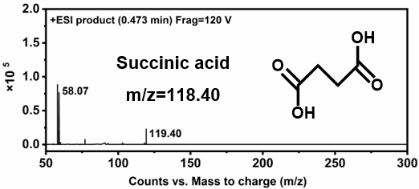


**Supplementary Fig. S9.** HPLC-MS spectra of NMP and its intermediates.

**
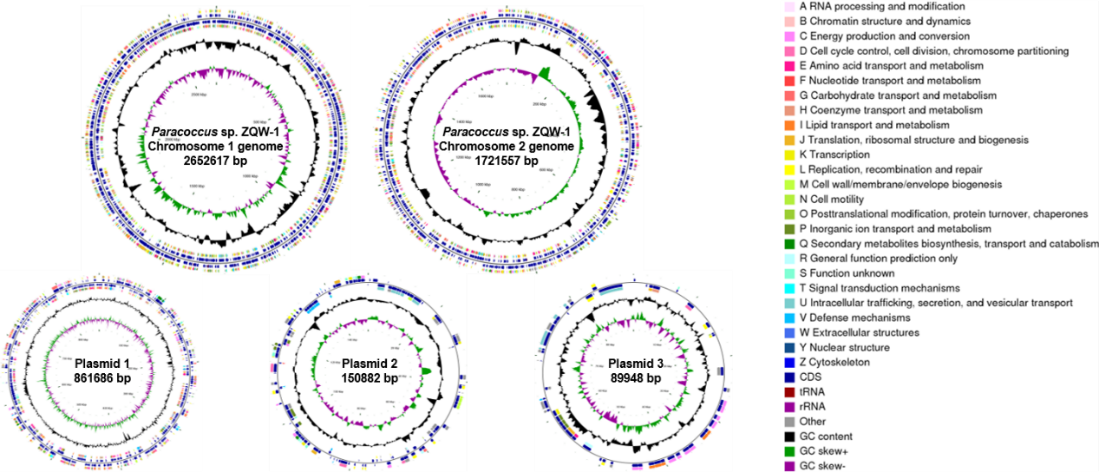
**

**Supplementary Fig. S10.** The genome and plasmid circular atlas of *Paracoccus* sp. ZQW-1.


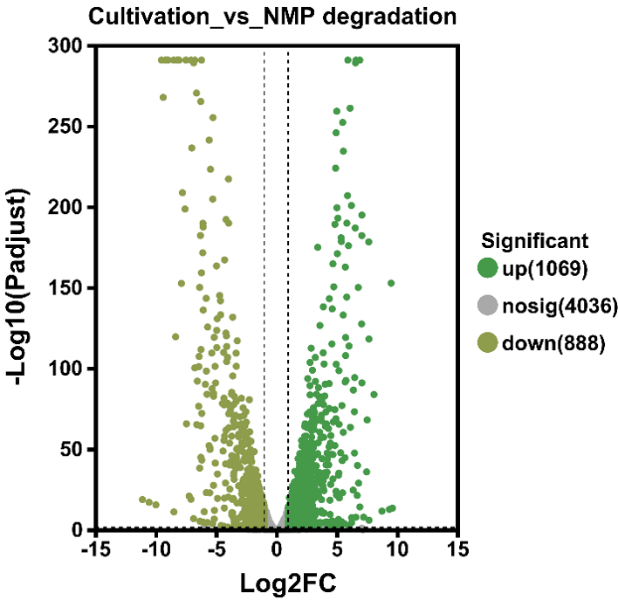


**Supplementary Fig. S11.** The volcano plot of differential genes under different groups (i.e., *Paracoccus* sp. ZQW-1 cultivation and NMP degradation process).


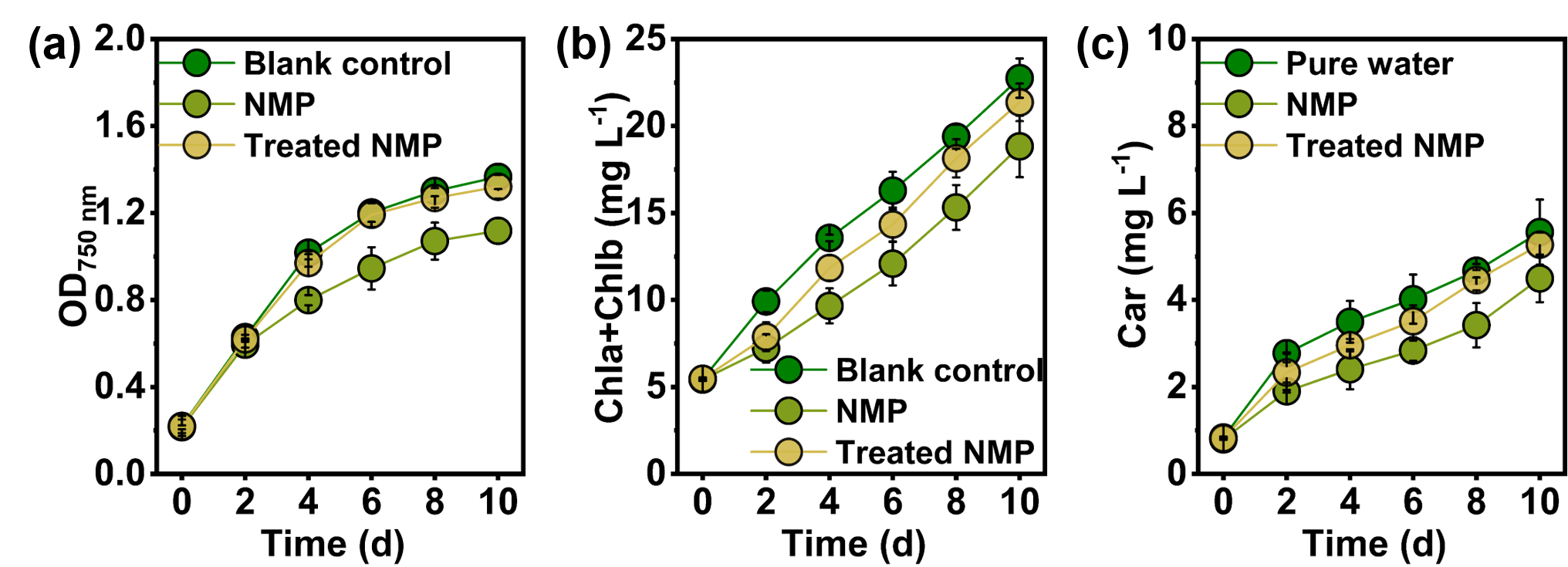


**Supplementary Fig. S12.** The toxic evolution of NMP wastewater evaluated by *Chlorella vulgaris*. **a,** The OD_750 nm_ of *Chlorella vulgaris* cultured in different water matrixes. **b, c,** The accumulation of chlorophyll a + chlorophyll b (Chla + Chlb) and carotenoid (Car) in different water matrixes.


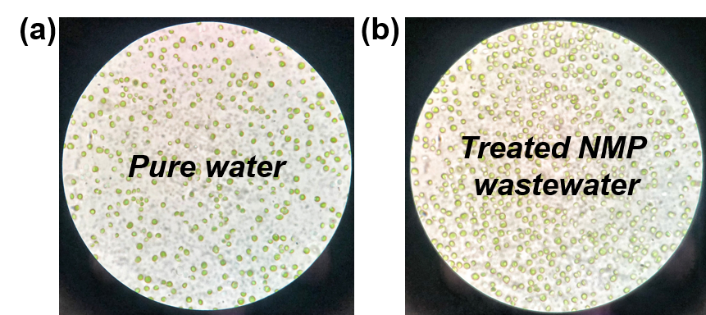


**Supplementary Fig. S13.** Microscopic observation of *Chlorella vulgaris* growth under various culture conditions. **a,** The BG11 medium was prepared with pure water. **b,** The BG11 medium was prepared with the treated NMP solution.


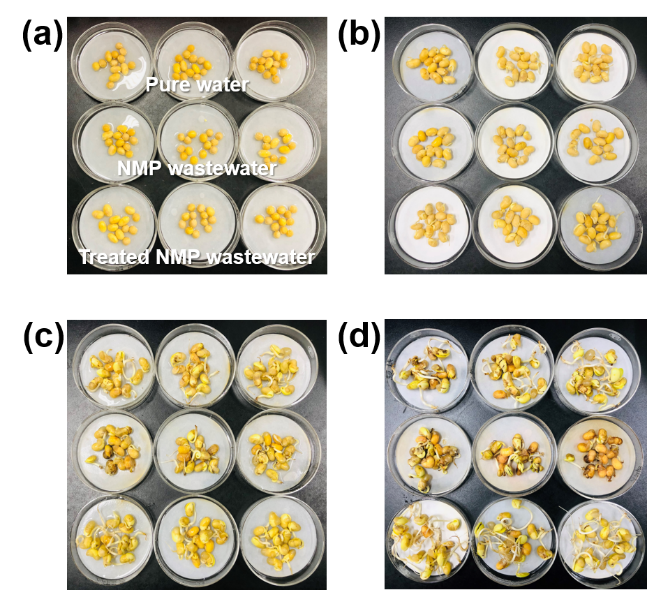


**Supplementary Fig. S14.** The phenotype of soybean seeds cultured by pure water, NMP solution, and the treated NMP solution. **a,** day 1. **b,** day 3. **c,** day 5. **d,** day 7.





**Supplementary Fig. S15.** The functional annotations of *Paracoccus* sp. ZQW-1 genome against the Gene Ontology categories (GO) database.

As shown in **Fig. S15**, the protein-coding genes of *Paracoccus* sp. ZQW-1 were annotated into three GO categories: molecular function (1928 genes), biological process (713 genes), and cellular component (1816 genes).





**Supplementary Fig. S16.** The Kyoto Encyclopedia of Genes and Genomes database (KEGG) categories of microbial metabolism of *Paracoccus* sp. ZQW-1.

As shown in **Fig. S16**, these gene sets could be mapped to the KEGG database to classify pathways, which suggested that *Paracoccus* sp. ZQW-1 could metabolize diverse substrates (e.g., sucrose, fructose, galactose, butanoate, propanoate, and starch) and cover the genes for pyruvate metabolism, TCA cycle, and glycolysis process (key steps involved in PHB production).


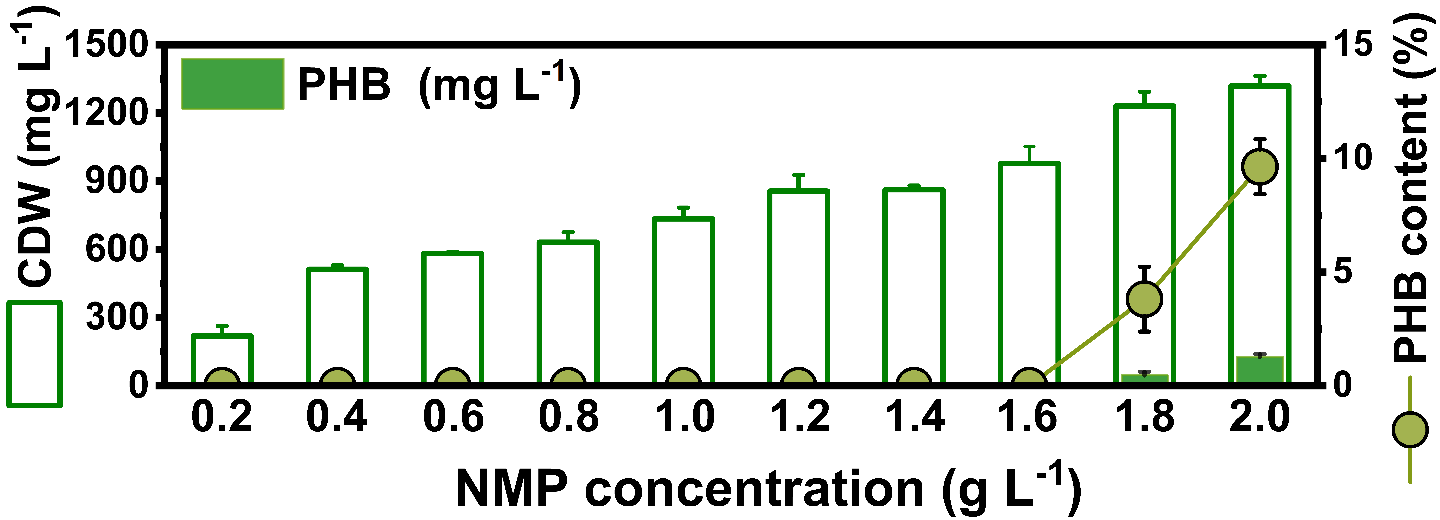


**Supplementary Fig. S17.** The accumulation of PHB during NMP degradation process. ([NMP] = 0.2−2.0 g L^−1^, [inoculum concentration] = 4%, [pH] = 7, [temperature] = 30°C).


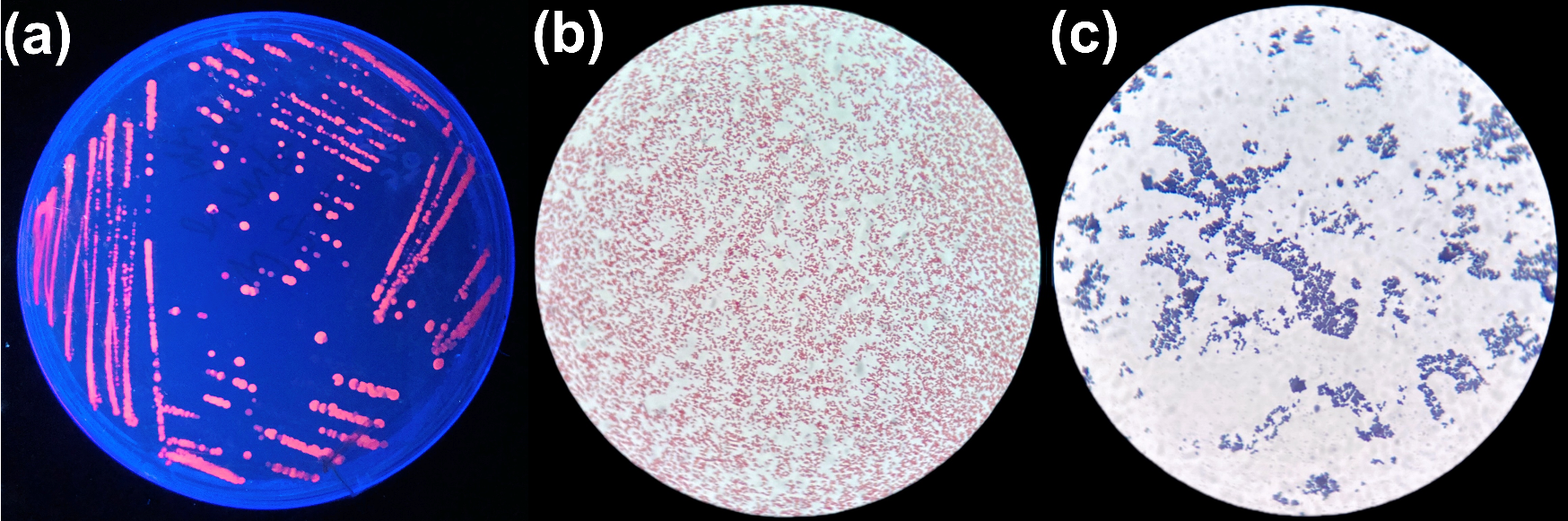


**Supplementary Fig. S18.** Identification of PHA synthesis function of *Paracoccus* sp. ZQW-1. **a,** Nile red staining methods (with 10 g L^−1^ glucose). **b,** Sudan black staining methods **(**without glucose**)**. **c,** Sudan black staining methods (with 10 g L^−1^ glucose).

Glucose was supplemented to improve the C/N ratio of culture solution. The Acetyl-CoA could be directed to PHA production rather than energy generation under the N-deficient conditions. As shown in **Fig. S18a**, the intracellular PHA was specifically bound with Nile red, emitting representative red fluorescence. Further staining by Sudan black stain confirmed the presence of PHA granules, which were against red cytoplasm counterstained with safranin (**Fig. S18b, c**).

**
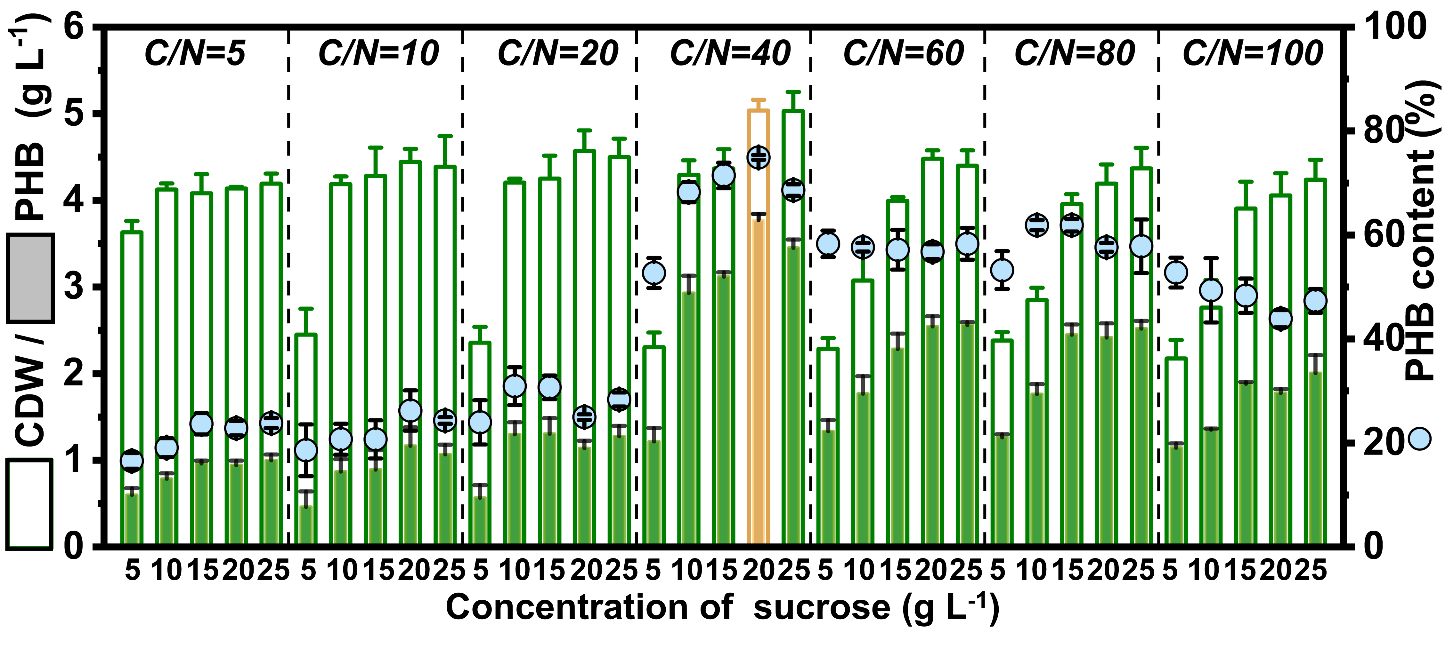
**

**Supplementary Fig. S19.** The effects of sucrose concentration (5−25 g L^−1^) and C/N ratio (5−100) on PHB synthesis. ([fermentation time] = 2 d, [temperature] = 30 ℃, [pH] = 8, and [inoculation dosage] = 4%).

The maximum PHB titer (3.77 g L^−1^) was obtained when the concentration of sucrose was 20 g L^−1^ and the C/N ratio was 40:1.


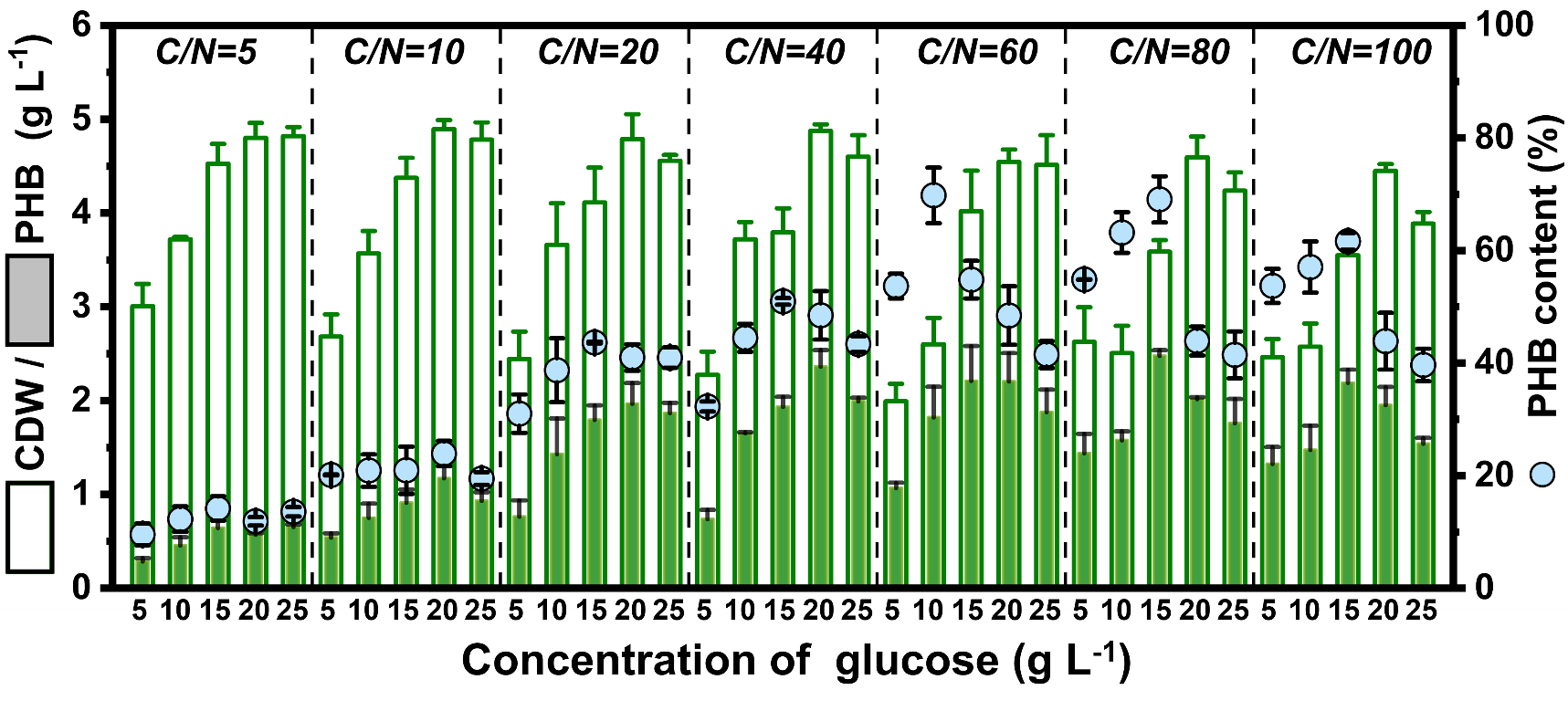


**Supplementary Fig. S20.** The effects of glucose concentration (5−25 g L^−1^) and C/N ratio (5−100) on PHB synthesis. ([fermentation time] = 2 d, [temperature] = 30 ℃, [pH] = 8, and [inoculation dosage] = 4%).

The maximum PHB titer (2.47 g L^−1^) was obtained when the concentration of glucose was 15 g L^−1^ and the C/N ratio was 80:1.


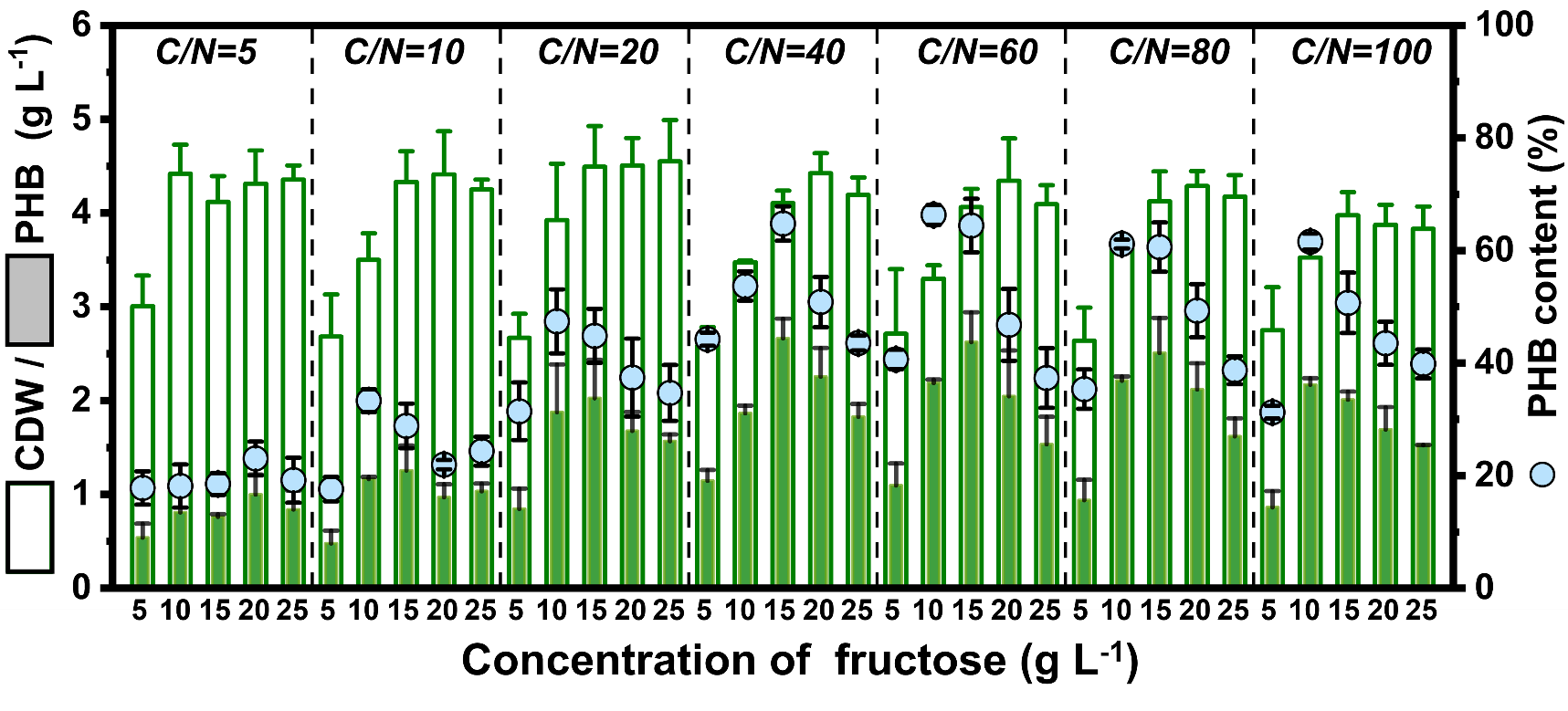


**Supplementary Fig. S21.** The effects of fructose concentration (5−25 g L^−1^) and C/N ratio (5−100) on PHB synthesis. ([fermentation time] = 2 d, [temperature] = 30 ℃, [pH] = 8, and [inoculation dosage] = 4%).

The maximum PHB titer (2.66 g L^−1^) was obtained when the concentration of fructose was 15 g L^−1^ and the C/N ratio was 40:1.


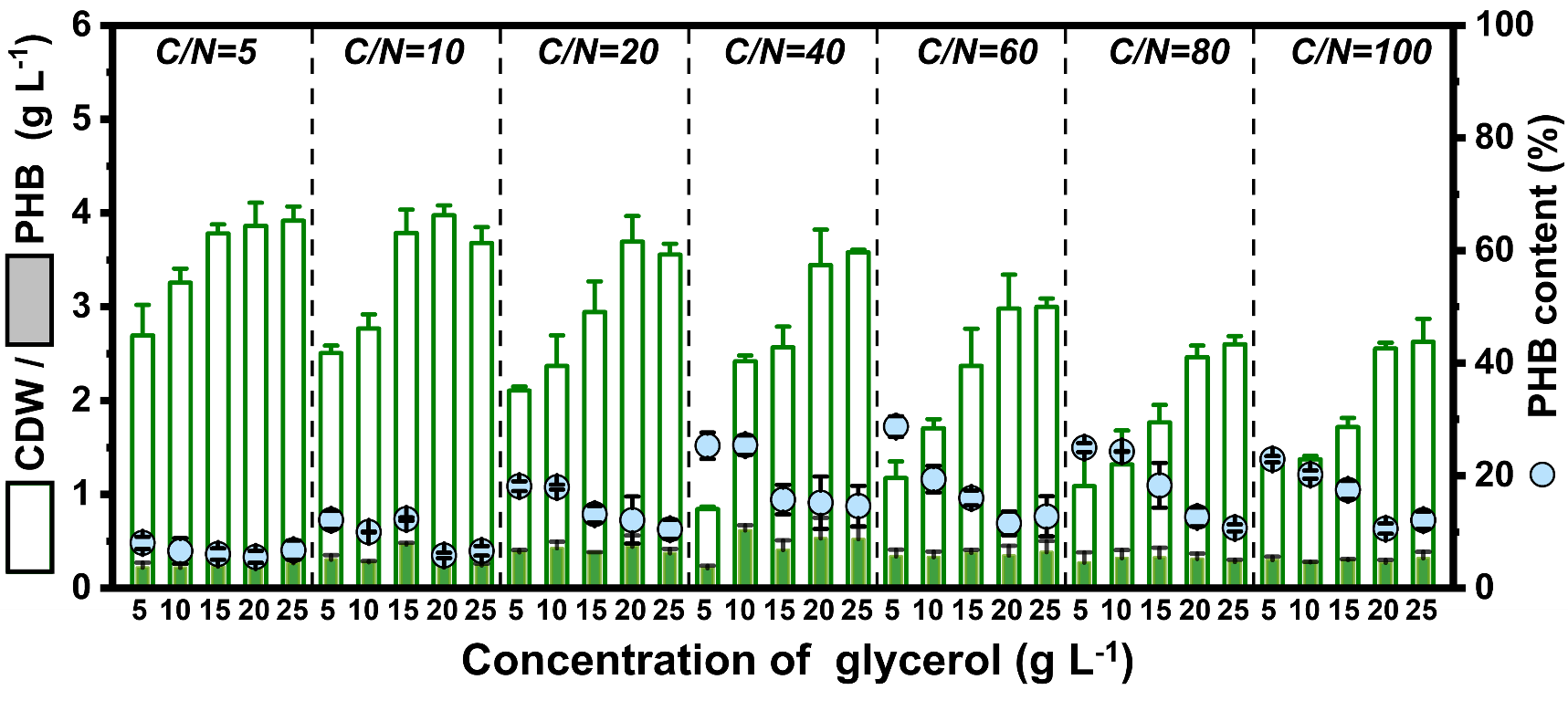


**Supplementary Fig. S22.** The effects of glycerin concentration (5−25 g L^−1^) and C/N ratio (5−100) on PHB synthesis. ([fermentation time] = 2 d, [temperature] = 30 ℃, [pH] = 8, and [inoculation dosage] = 4%).

The maximum PHB titer (0.62 g L^−1^) was obtained when the concentration of glycerol was 10 g L^−1^ and the C/N ratio was 40:1.


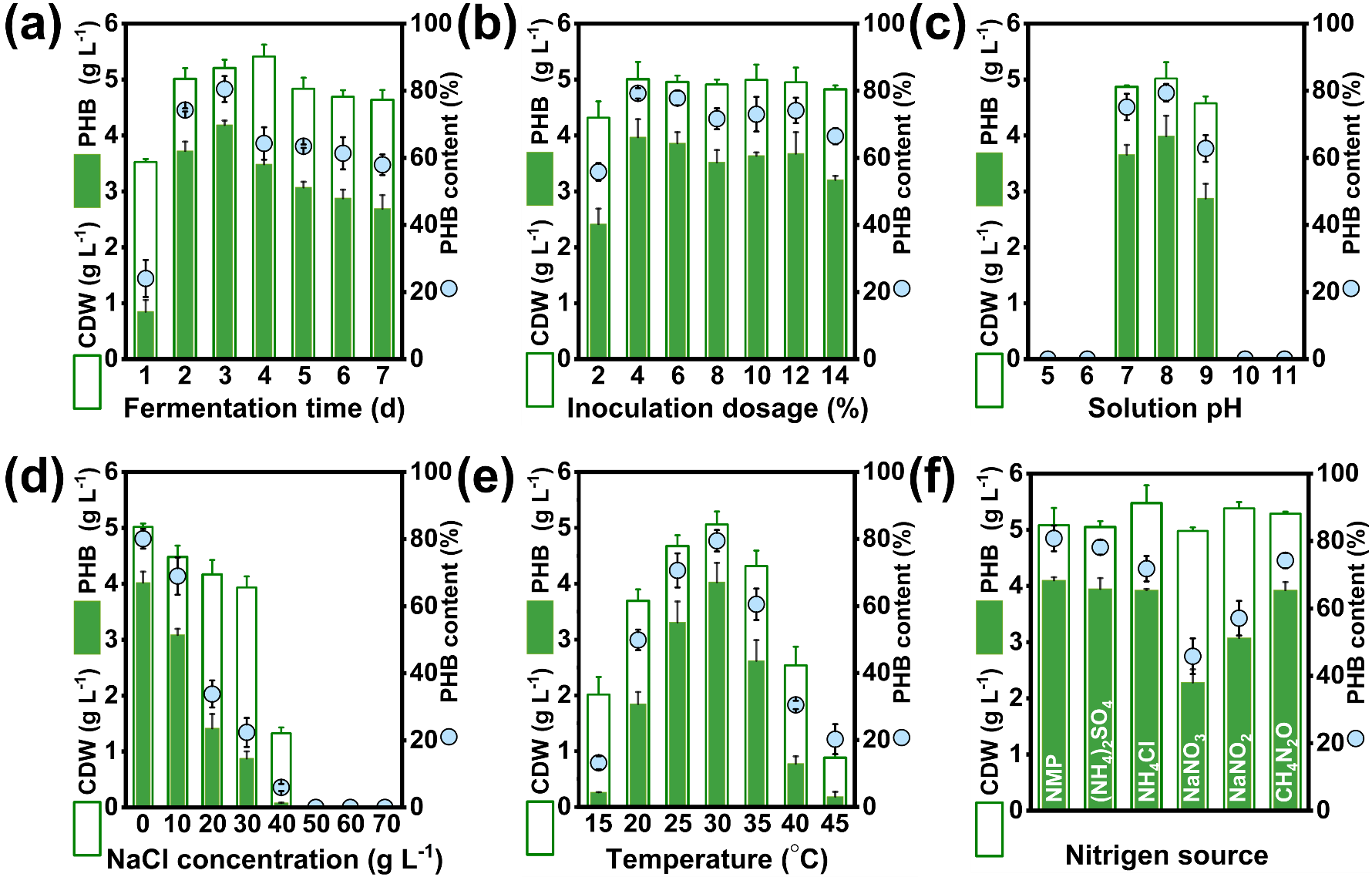


**Supplementary Fig. S23.** The effects of fermentation conditions on PHB synthesis. **a,** Fermentation time (1−7 d). Experimental conditions: [sucrose] = 20 g L^−1^, [C/N] = 40, and [inoculation dosage] = 4%. **b,** Inoculation dosage (2−14 %). **c,** pH value (5−11). **d,** NaCl concentration (0−70 g L^−1^). **e,** Temperature (15−45 ℃). **f,** Nitrogen source.

Fermentation parameters were optimized to improve PHB production. 4.19 g L^−1^ PHB was accumulated within 3 days, which was decreased to 2.69 g L^−1^ by prolonging fermentation time, probably owing to the self-consumption of PHB (**Fig. S23a**). Moreover, the optimal inoculation dosage was determined to be 4% (**Fig. S23b**). In addition, *Paracoccus* sp. ZQW-1 could more efficiently accumulate PHB at the optimal growth conditions (**Fig. S23c-e**, pH 8, 0 g L^−1^ NaCl, and 30 ℃). Notably, as compared to conventional nitrogen sources such as (NH_4_)_2_SO_4_, (NH_4_)Cl, NaNO_3_, CH_4_N_2_O, and NaNO_2_, superior PHB productivity was obtained using NMP as the specific nitrogen source (**Fig. S23f**).


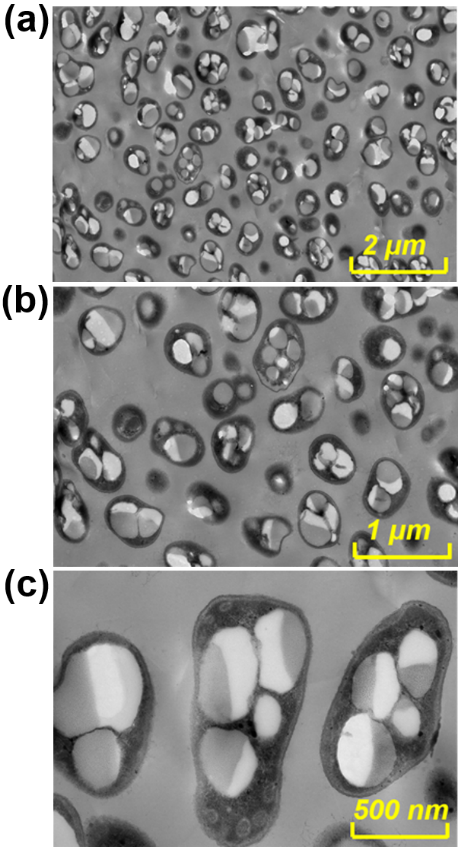


**Supplementary Fig. S24.** The characterizations of intracellular PHB granules by TEM.

The PHB granules occupied most space of the cells (~80%), which demonstrated that *Paracoccus* sp. ZQW-1 served as a promising chassis cell for PHB accumulation.


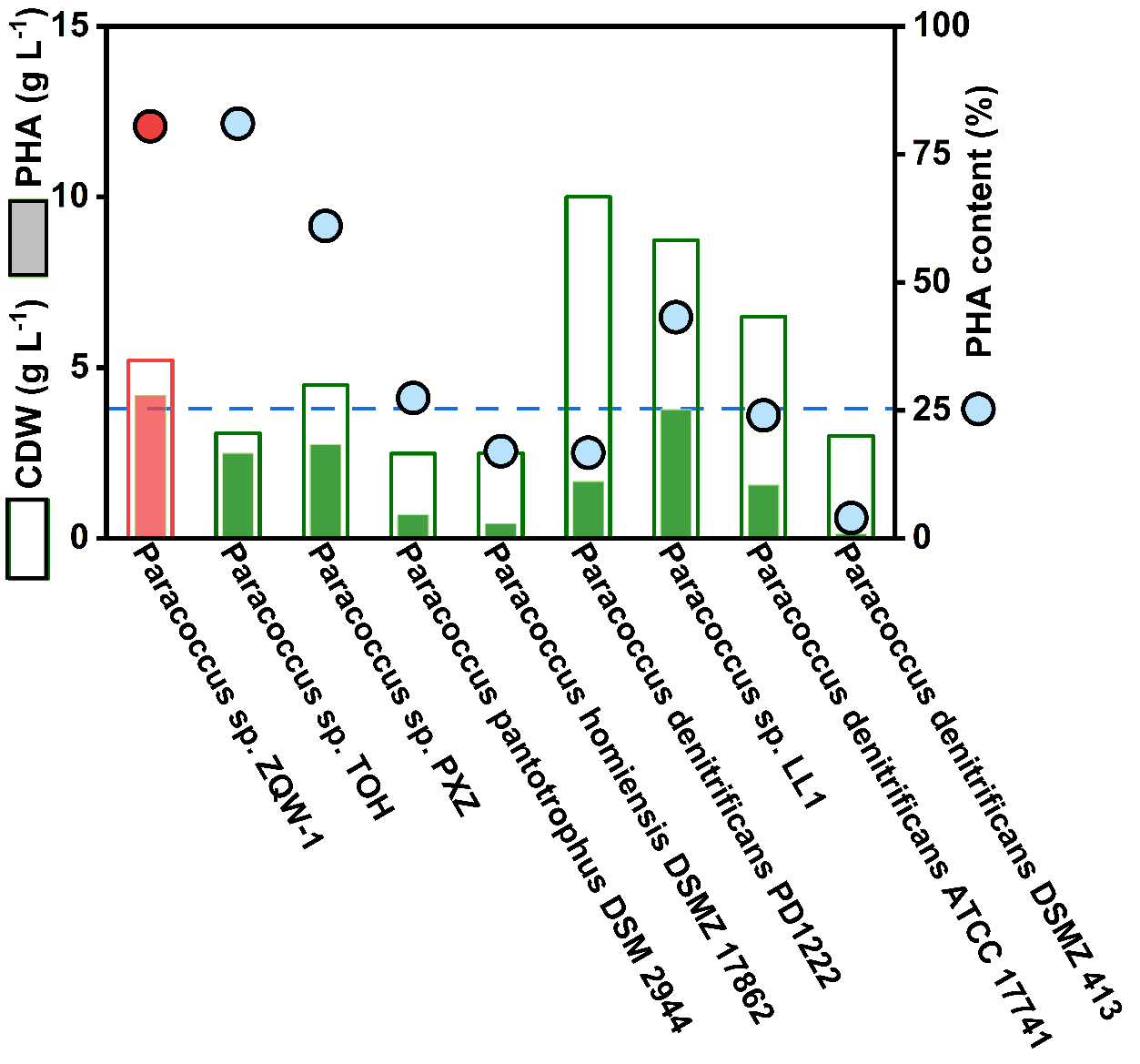


**Supplementary Fig. S25.** The comparison of PHA synthesis by *Paracoccus* sp. ZQW-1 and other documented *Paracoccus* strains.


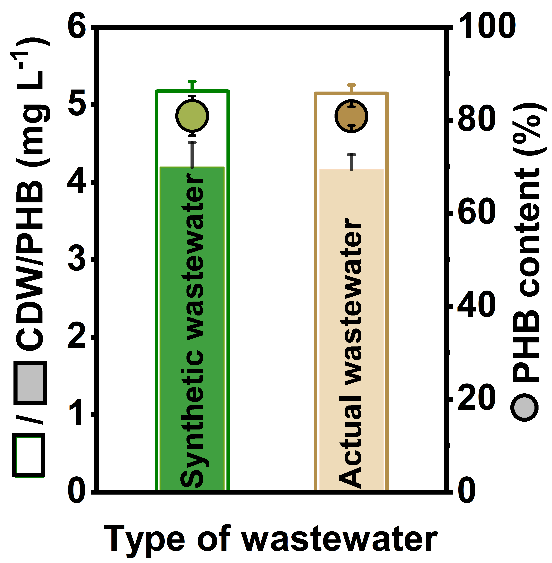


**Supplementary Fig. S26.** The comparison of PHB production by *Paracoccus* sp. ZQW-1 using synthetic and actual NMP wastewaters as nitrogen sources under optimal fermentation conditions. ([sucrose] = 20 g L^−1^, [C/N] = 40, [fermentation time] = 3 d, [temperature] = 30 ℃, [pH] = 8, and [inoculation dosage] = 4%).


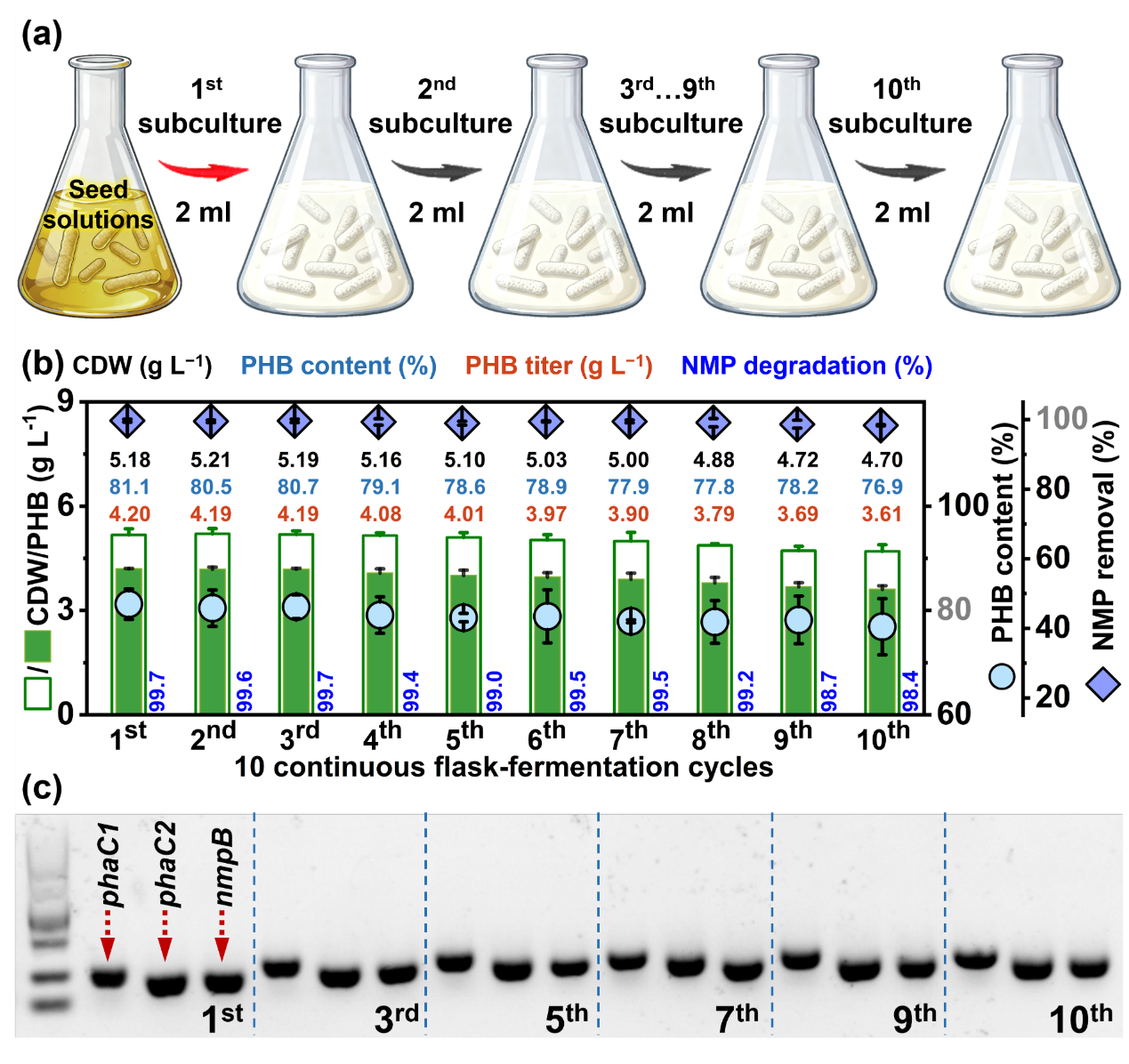


**Supplementary Fig. S27.** Continuous production of PHB and degradation of NMP under selective pressure. **a,** The schematic diagram of serial subculturing experiments. **b,** The PHB production and NMP removal during each subculture process. **c,** The PCR amplification of genes *phaC1*, *phaC2*, and *nmpB* during each subculture process.

**
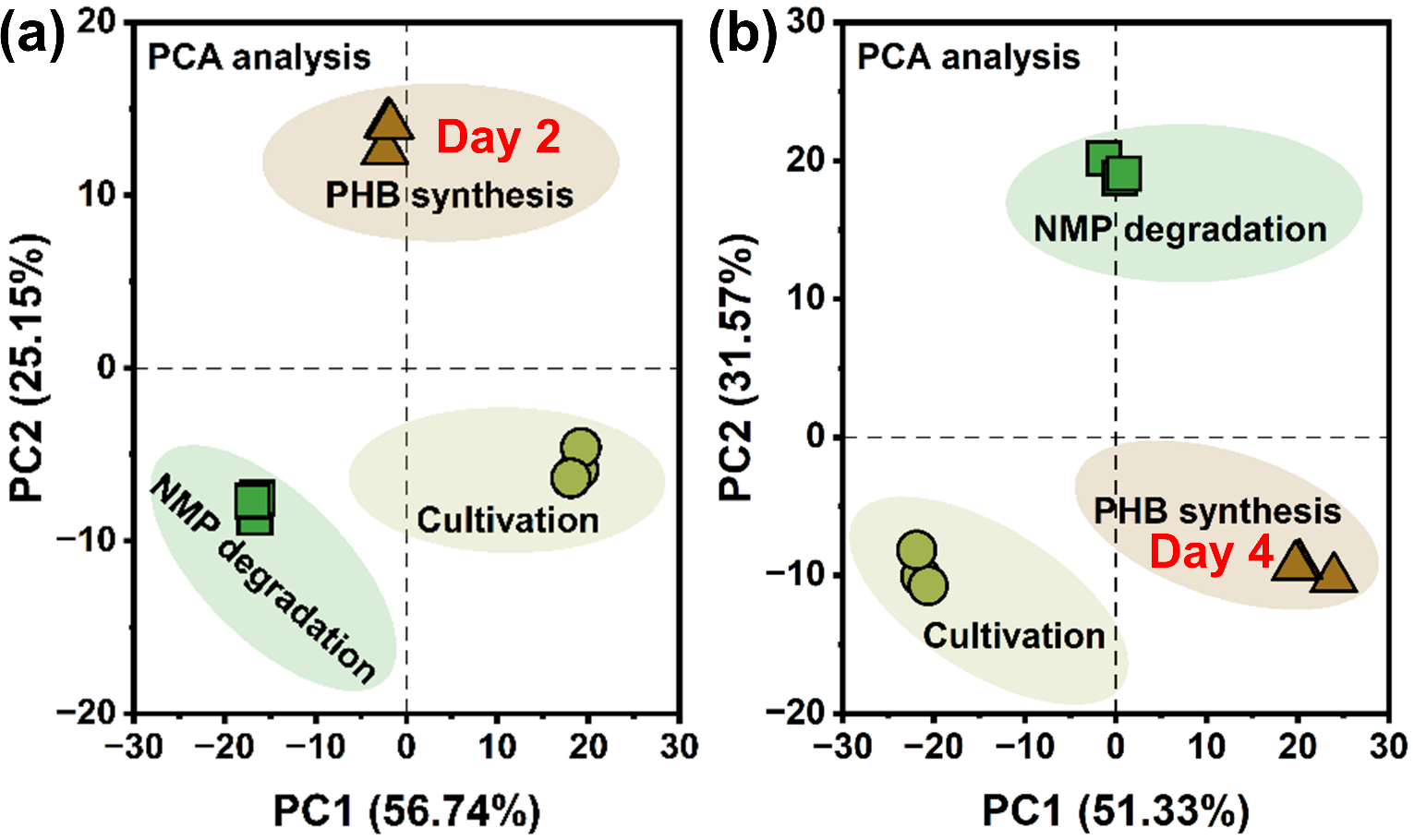
**

**Supplementary Fig. S28.** The principal component analysis (PCA) of *Paracoccus* sp. ZQW-1 under different groups (i.e., *Paracoccus* sp. ZQW-1 cultivation, NMP degradation stage, and PHB synthesis stage). **a,** The second day of fermentation corresponded to PHB accumulation stage (Day 2). **b,** The fourth day of fermentation corresponded to PHB consumption stage (Day 4).

The principal component analysis demonstrated that the three replicates of each treatment grouped together and significant deviations were observed among different treatment groups, which indicated the significantly divergent transcriptional responses in *Paracoccus* sp. ZQW-1 among different experimental groups.

**
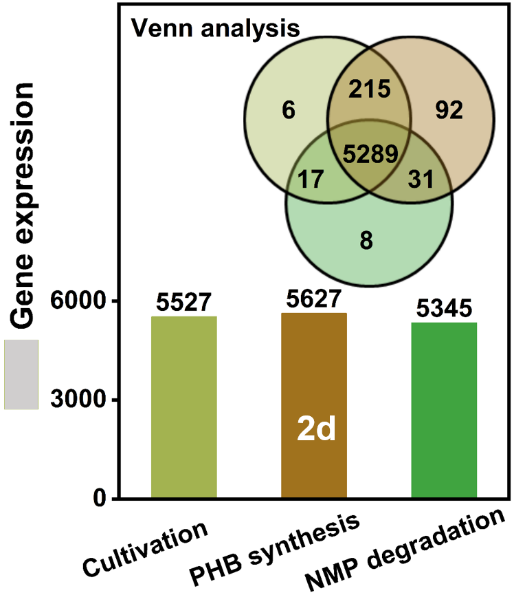
**

**Supplementary Fig. S29.** The Venn diagram of transcriptome regulation of *Paracoccus* sp. ZQW-1 under different groups (i.e., *Paracoccus* sp. ZQW-1 cultivation, NMP degradation, and PHB synthesis process (PHB accumulation stage, Day 2)) to illustrate the overlap of functional genes.

Obviously, Venn diagram and numbers of differentially expressed genes (DEGs) suggested that different treatments activated some genes, respectively. Specifically, 5504 genes were shared between *Paracoccus* sp. ZQW-1 cultivation stage and PHB accumulation stage, 5320 genes between PHB accumulation stage and NMP degradation stage, and 5306 genes between NMP degradation stage and *Paracoccus* sp. ZQW-1 cultivation stage. In addition, 5289 genes were shared across all three treatment groups.

**
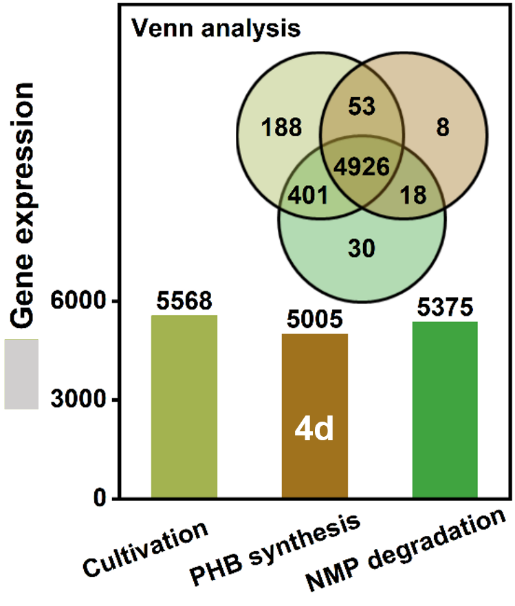
**

**Supplementary Fig. S30.** The Venn diagram of transcriptome regulation of *Paracoccus* sp. ZQW-1 under different groups (i.e., *Paracoccus* sp. ZQW-1 cultivation, NMP degradation, and PHB synthesis process (PHB consumption stage, Day 4)) to illustrate the overlap of functional genes.

Specifically, 4979 genes were shared between *Paracoccus* sp. ZQW-1 cultivation stage and PHB consumption stage, 4934 genes between PHB consumption stage and NMP degradation stage, and 5327 genes between NMP degradation stage and *Paracoccus* sp. ZQW-1 cultivation stage. In addition, 4926 genes were shared across all three treatment groups.

**
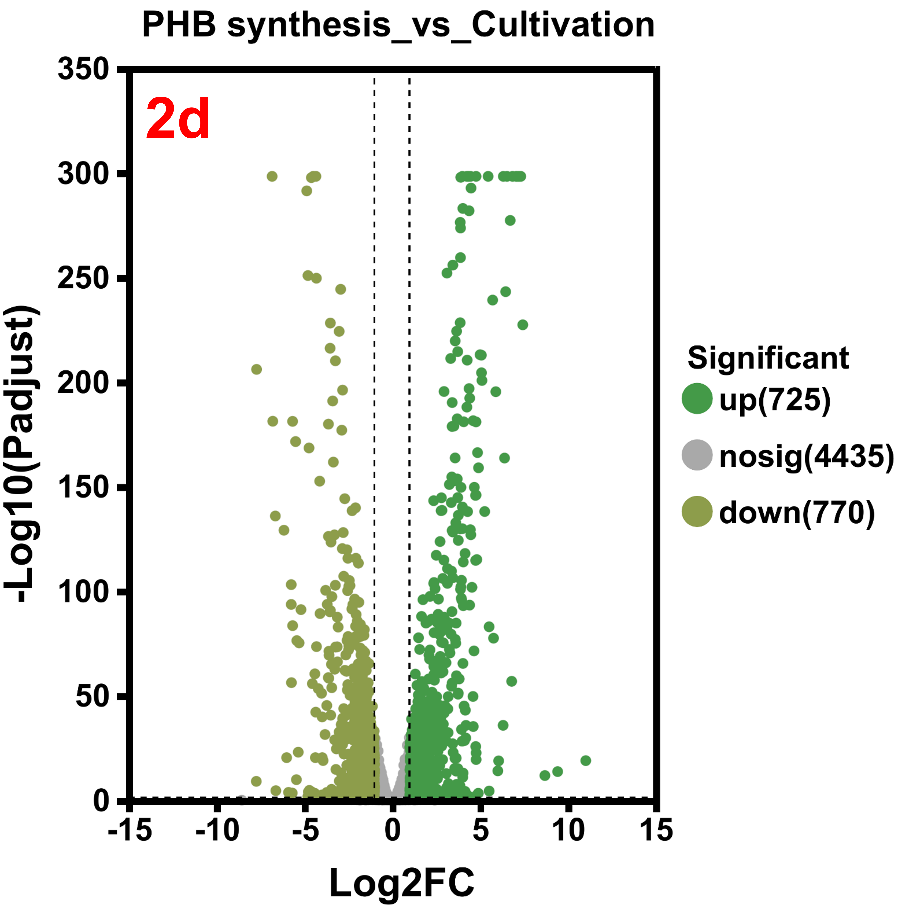
**

**Supplementary Fig. S31.** The volcano plot of differential genes under different groups (i.e., *Paracoccus* sp. ZQW-1 cultivation and PHB synthesis process (PHB accumulation stage, Day 2)).

The volcano plots revealed that 725 genes were up-regulated and 770 genes were down-regulated in PHB accumulation stage, as compared to *Paracoccus* sp. ZQW-1 cultivation stage.

**
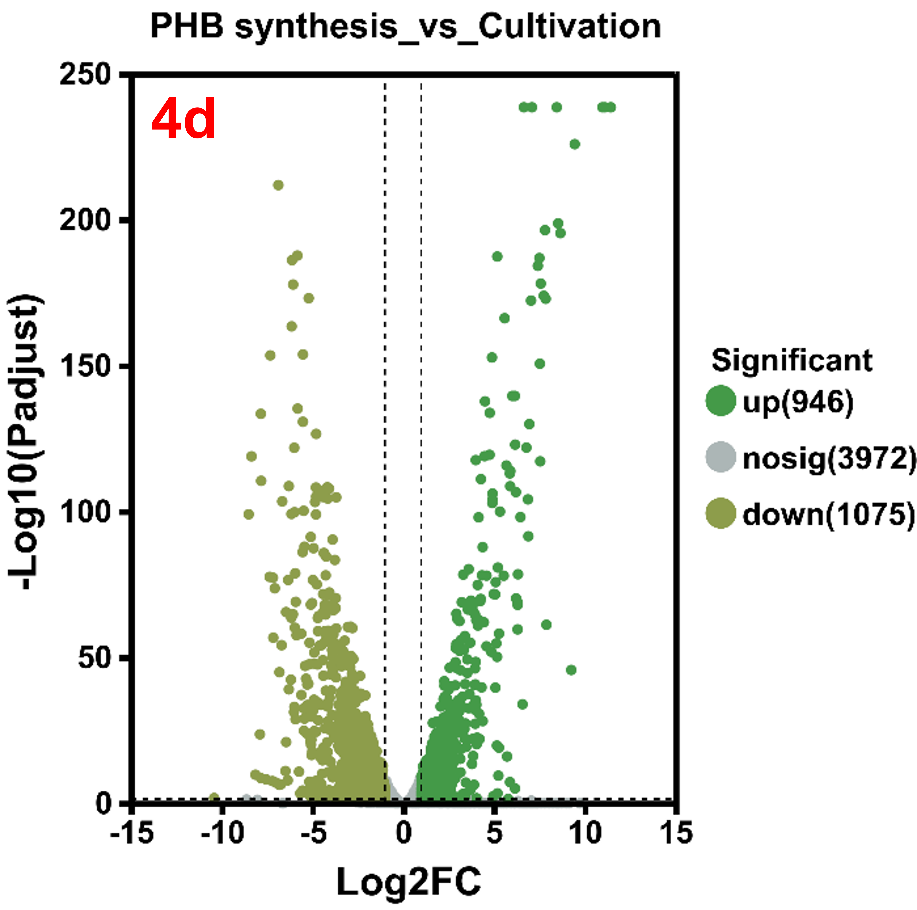
**

**Supplementary Fig. S32.** The volcano plot of differential genes under different groups (i.e., *Paracoccus* sp. ZQW-1 cultivation and PHB synthesis process (PHB consumption stage, Day 4)).

The volcano plots revealed that there were 2021 DEGs between *Paracoccus* sp. ZQW-1 cultivation stage and PHB consumption stage, of which 946 genes were up-regulated and 1075 genes were down-regulated.

**
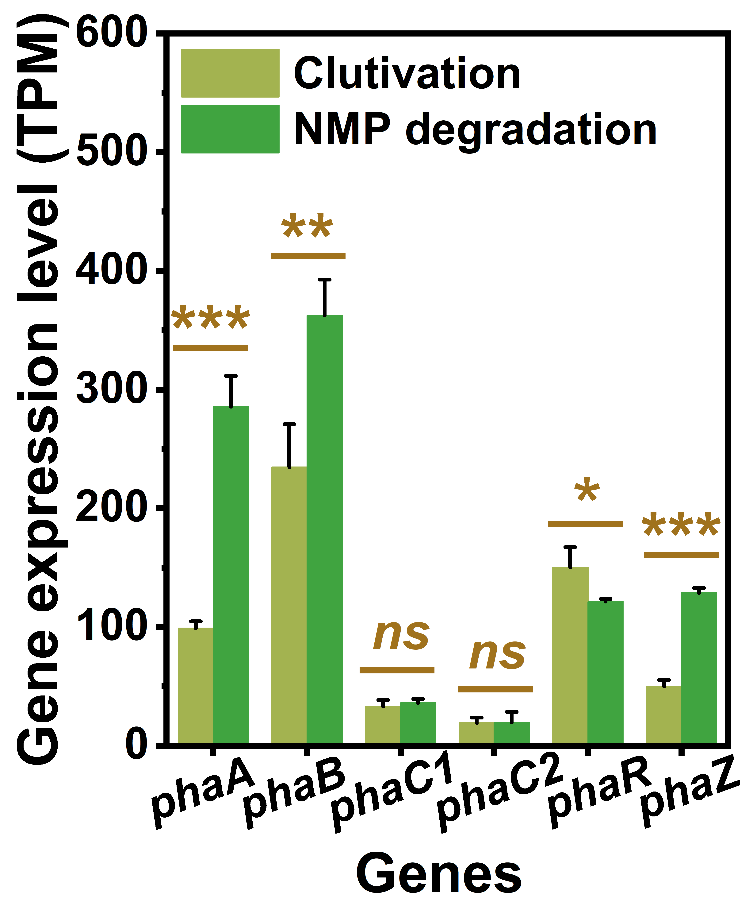
**

**Supplementary Fig. S33.** The expression levels of genes responsible for PHA synthesis under different groups (i.e., *Paracoccus* sp. ZQW-1 cultivation and NMP degradation stage) by transcript per million (TPM) method.

**
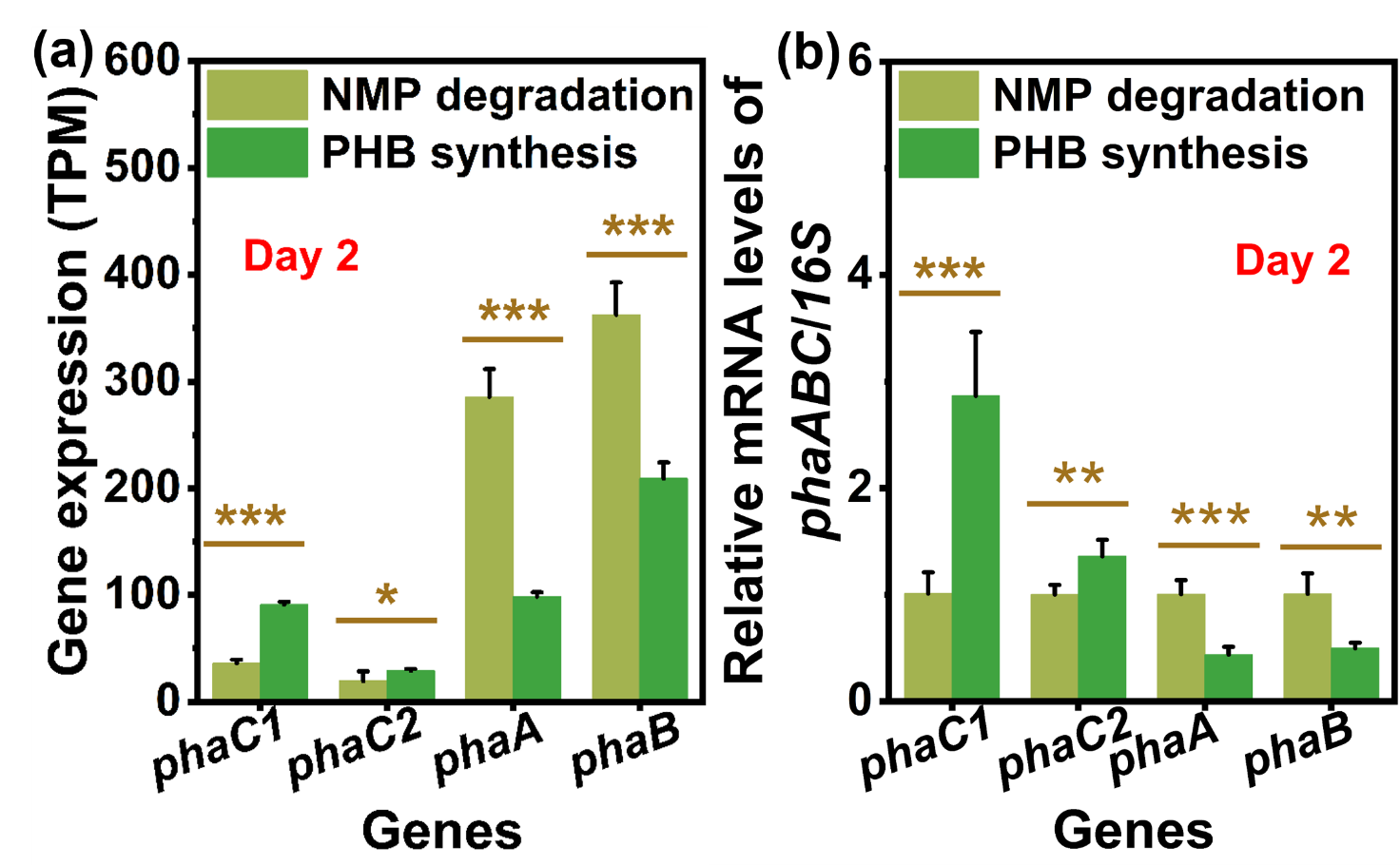
**

**Supplementary Fig. S34.** The expression levels of *phaA*, *phaB*, and *phaC* under different groups (i.e., NMP degradation stage and PHB synthesis stage (Day 2)) obtained by transcriptome analysis and RT-qPCR. **a,** Transcriptome analysis. **b,** RT-qPCR results.

**
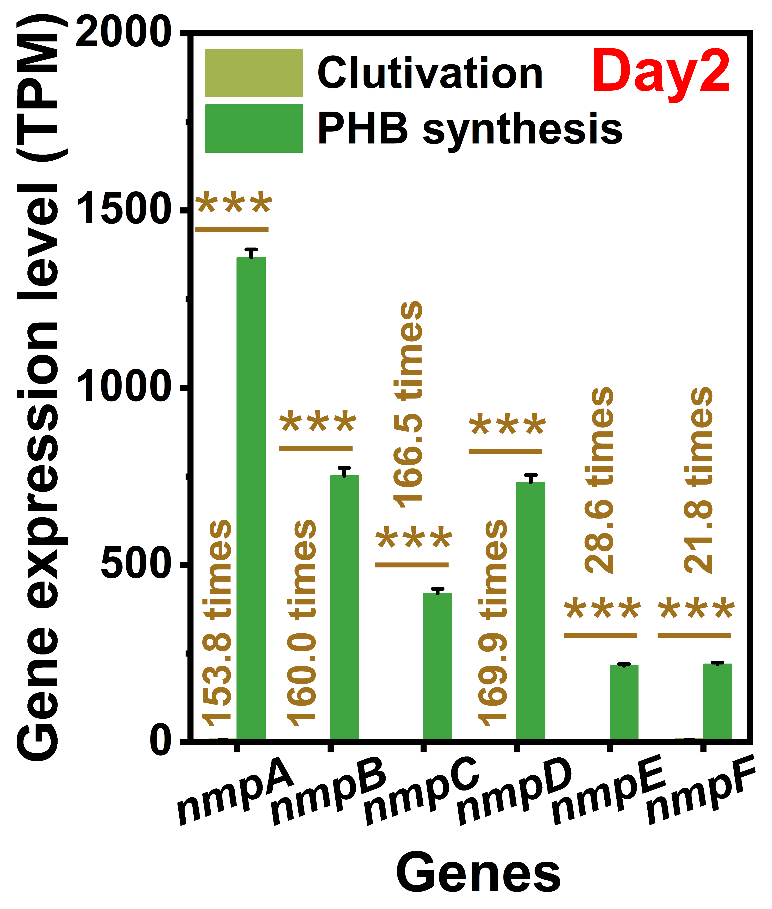
**

**Supplementary Fig. S35.** The expression levels of NMP-degrading genes under different groups (i.e., *Paracoccus* sp. ZQW-1 cultivation and PHB synthesis process (PHB accumulation stage, Day 2)) by transcript per million (TPM) method.

**
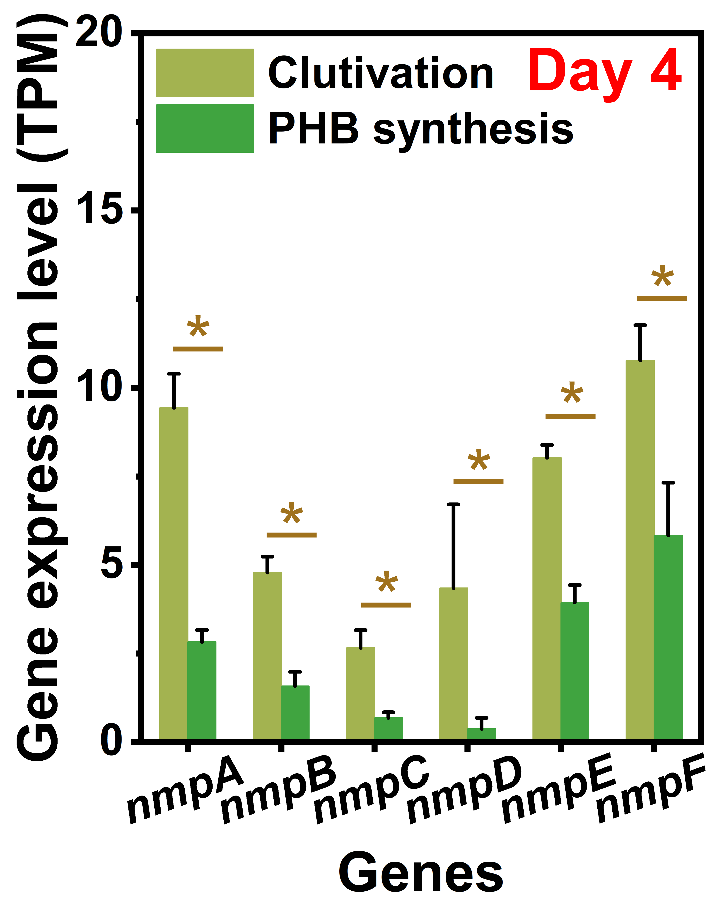
**

**Supplementary Fig. S36.** The expression levels of NMP-degrading genes under different groups (i.e., *Paracoccus* sp. ZQW-1 cultivation and PHB synthesis process (PHB consumption stage, Day 4)) by transcript per million (TPM) method.


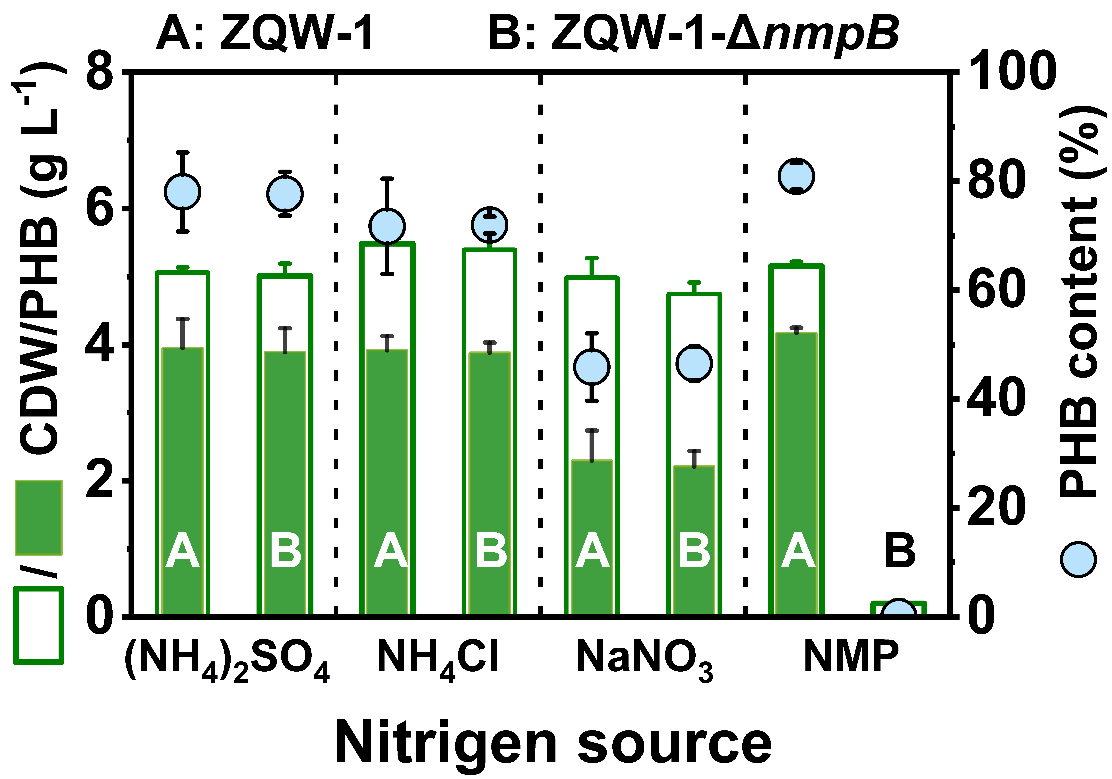


**Supplementary Fig. S37.** The comparison of PHB production by *Paracoccus* sp. ZQW-1 and *Paracoccus* sp. ZQW-1-Δ*nmpB* under different nitrogen sources. ([sucrose] = 20 g L^−1^, [C/N] = 40, and [inoculation dosage] = 4%).


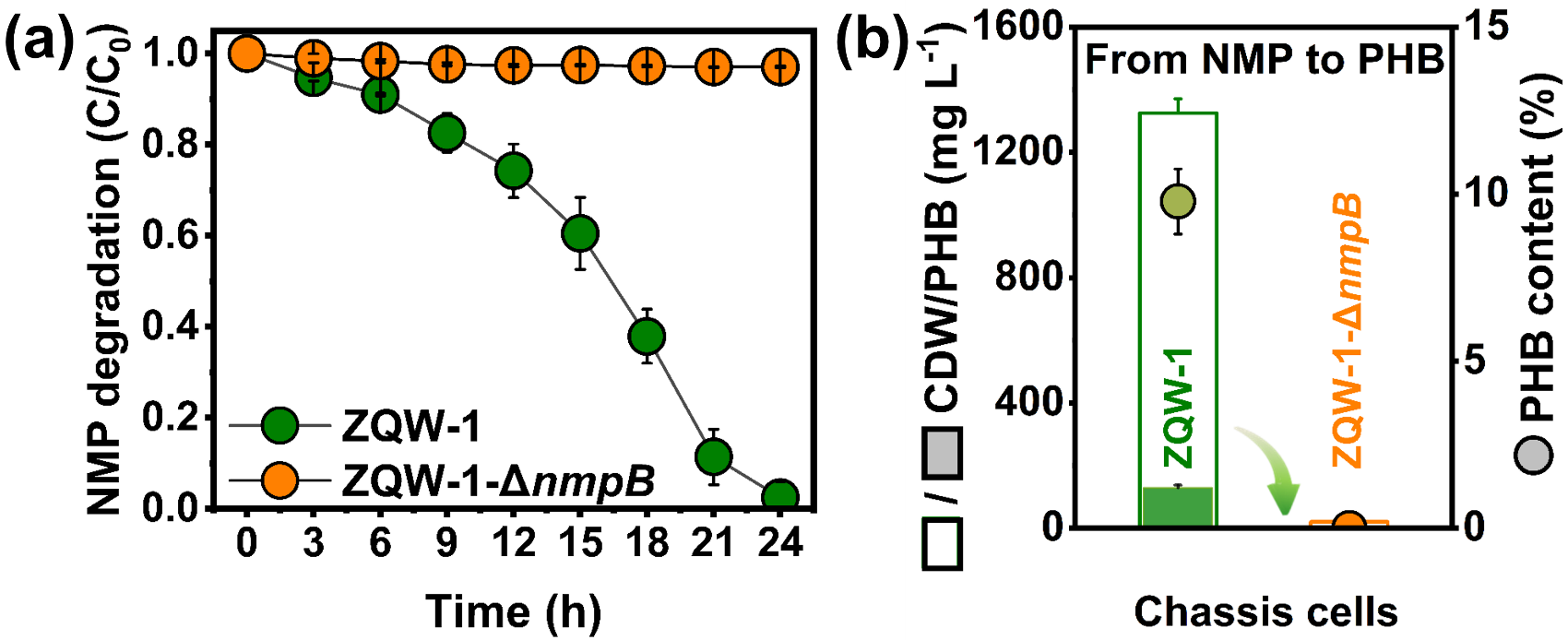


**Supplementary Fig. S38.** The NMP degradation and PHB synthesis capabilities of *Paracoccus* sp. ZQW-1-Δ*nmpB*. **a,** Degradation of 1 g L^−1^ NMP. **b,** Synthesis of PHB by using 2 g L^−1^ NMP as the substrate.


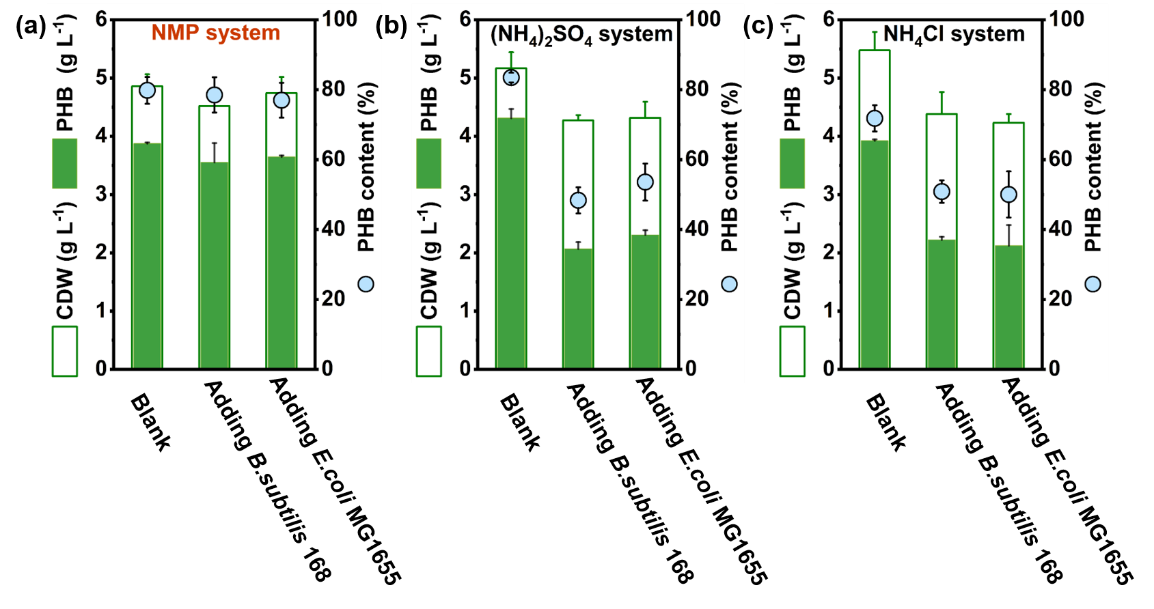


**Supplementary Fig. S39.** The PHB production by co-culture of *Paracoccus* sp. ZQW-1 and representative microbial competitors (i.e., *B. subtilis* 168 and *E. coli* MG1655) using different nitrogen sources. **a,** NMP. **b,** (NH_4_)_2_SO_4_. **c,** NH_4_Cl.


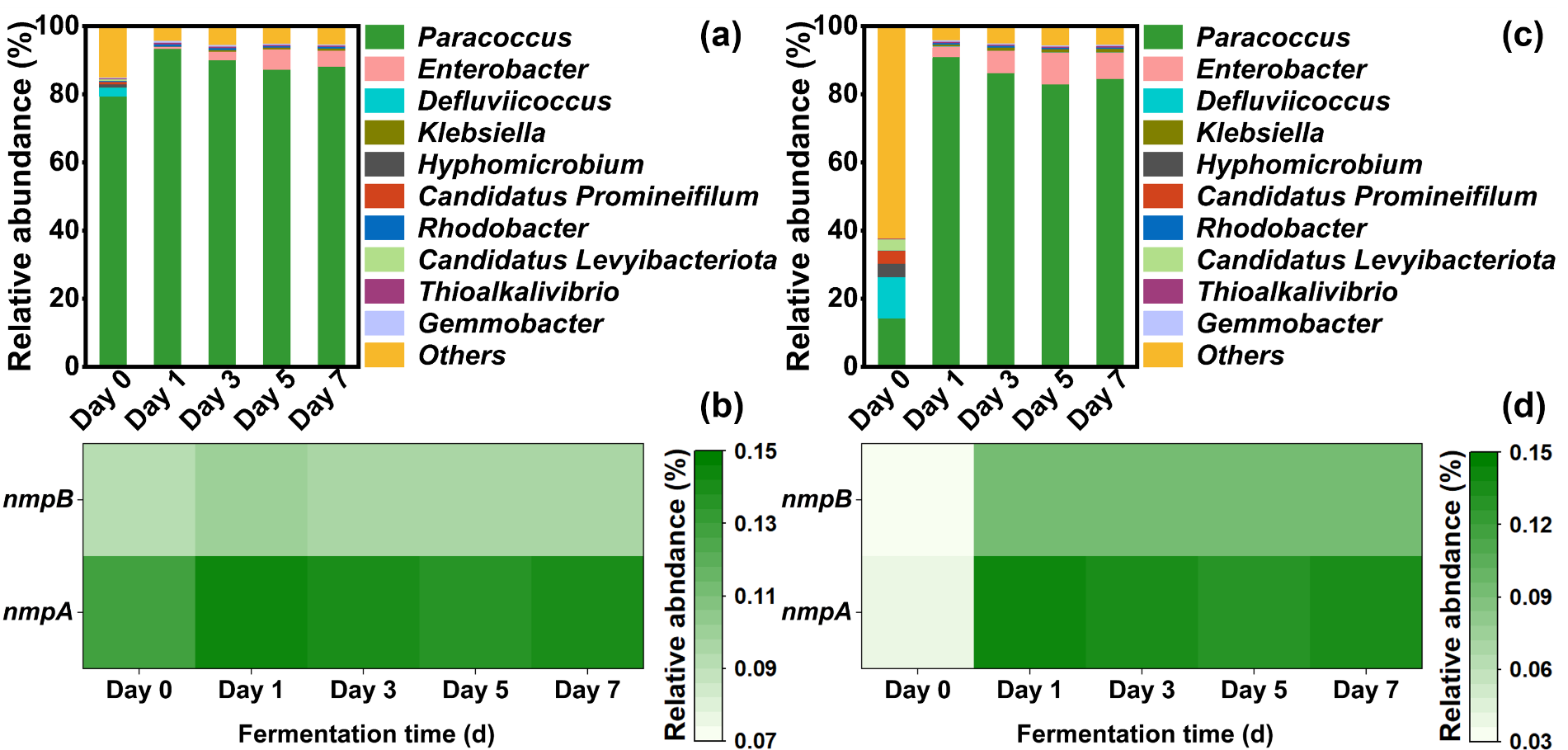


**Supplementary Fig. S40.** Elucidating the microbial community dynamics in NMP-driven PHB fermentation systems by metagenomic analyses. **a,** The inoculum concentration of *Paracoccus* sp. ZQW-1 and activated sludge was 4% and 0.4%, respectively. **b,** The corresponding abundance changes of *nmpA* and *nmpB* in **Fig. S40a**. **c,** The inoculum concentration of *Paracoccus* sp. ZQW-1 and activated sludge was 4% and 8%, respectively. **d,** The corresponding abundance changes of *nmpA* and *nmpB* in **Fig. S40c**.


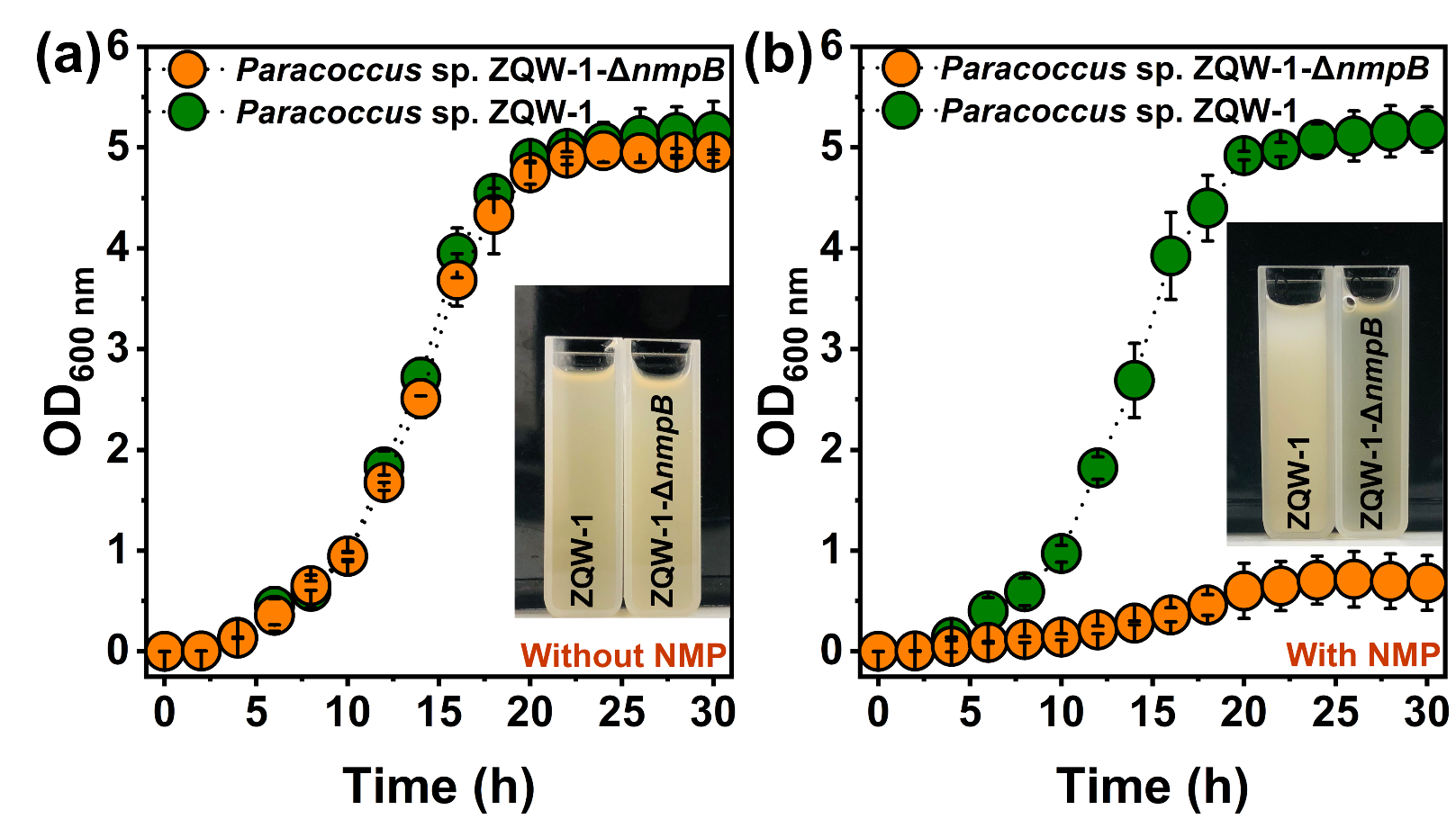


**Supplementary Fig. S41.** The growth of *Paracoccus* sp. ZQW-1 and *Paracoccus* sp. ZQW-1-Δ*nmpB* in LB medium. **a,** Growth curves without selective pressure. **b,** Growth curves under selective pressure imposed by NMP (1.0 g L^−1^).


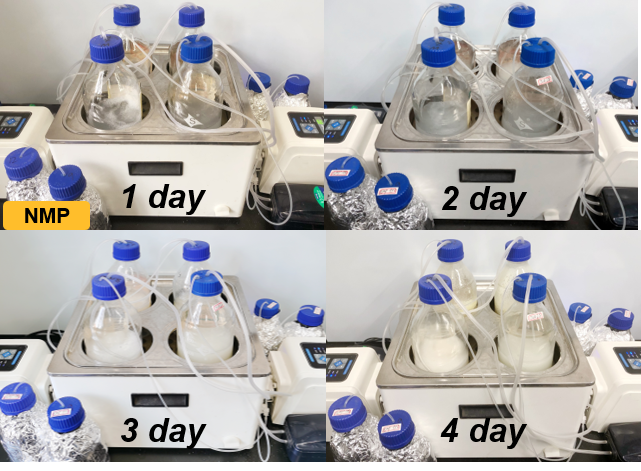


**Supplementary Fig. S42.** The picture of semi-continuous PHB production (1-L scale) using simulated NMP wastewater as the nitrogen source under non-sterile conditions.


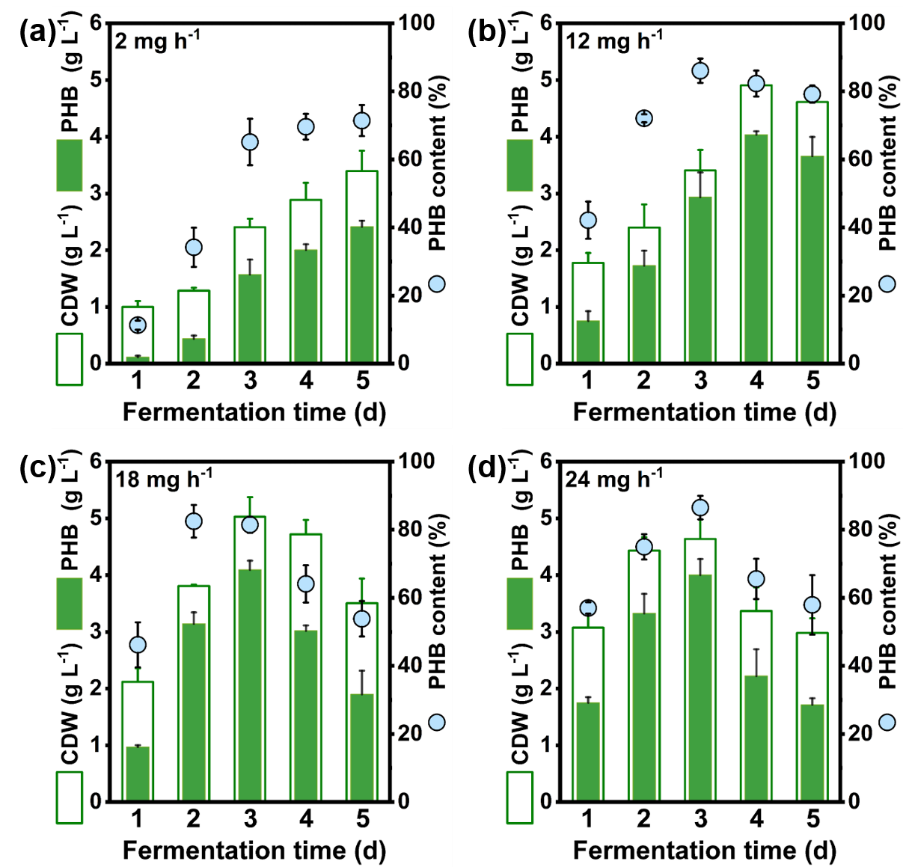


**Supplementary Fig. S43.** The non-sterile PHB production in a 1-L bioreactor by controlling the feeding amount of NMP. **a,** 2 mg h^−1^. **b,** 12 mg h^−1^. **c,** 18 mg h^−1^. **d,** 24 mg h^−1^.


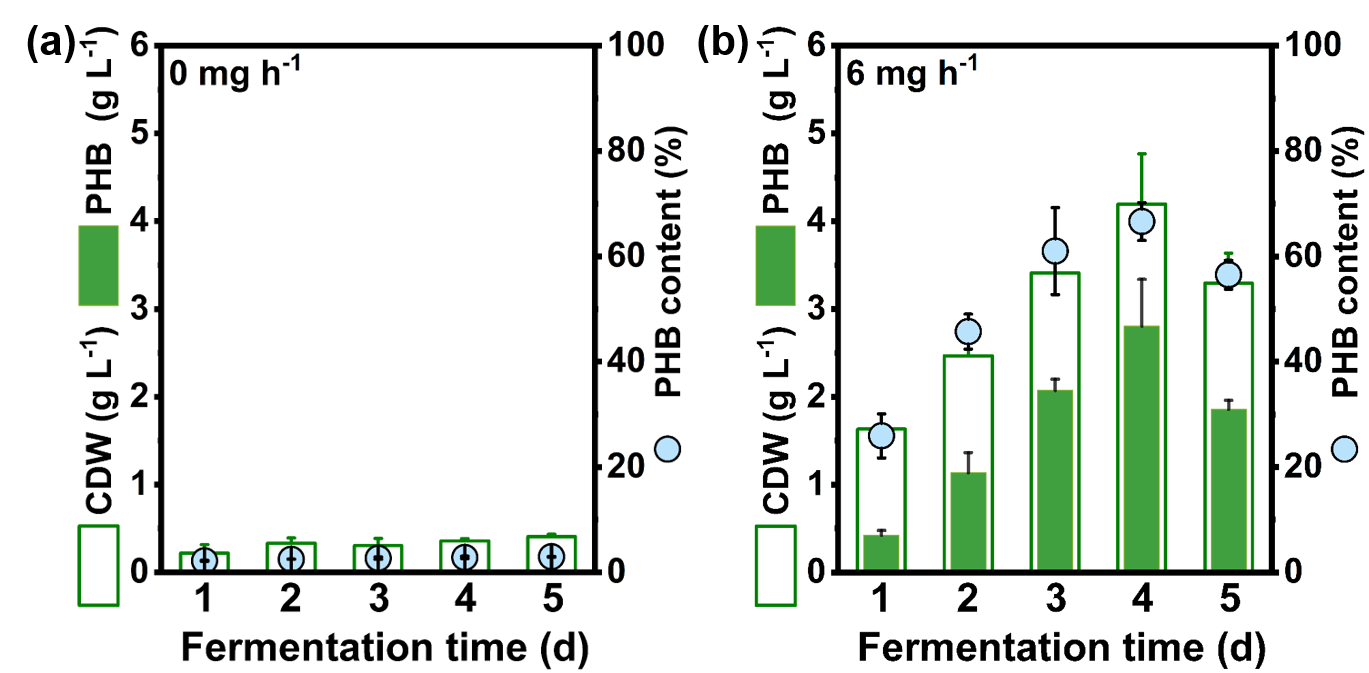


**Supplementary Fig. S44.** The non-sterile PHB production in a 1-L bioreactor by controlling the feeding amount of (NH_4_)_2_SO_4_. **a,** 0 mg h^−1^. **b,** 6 mg h^−1^.


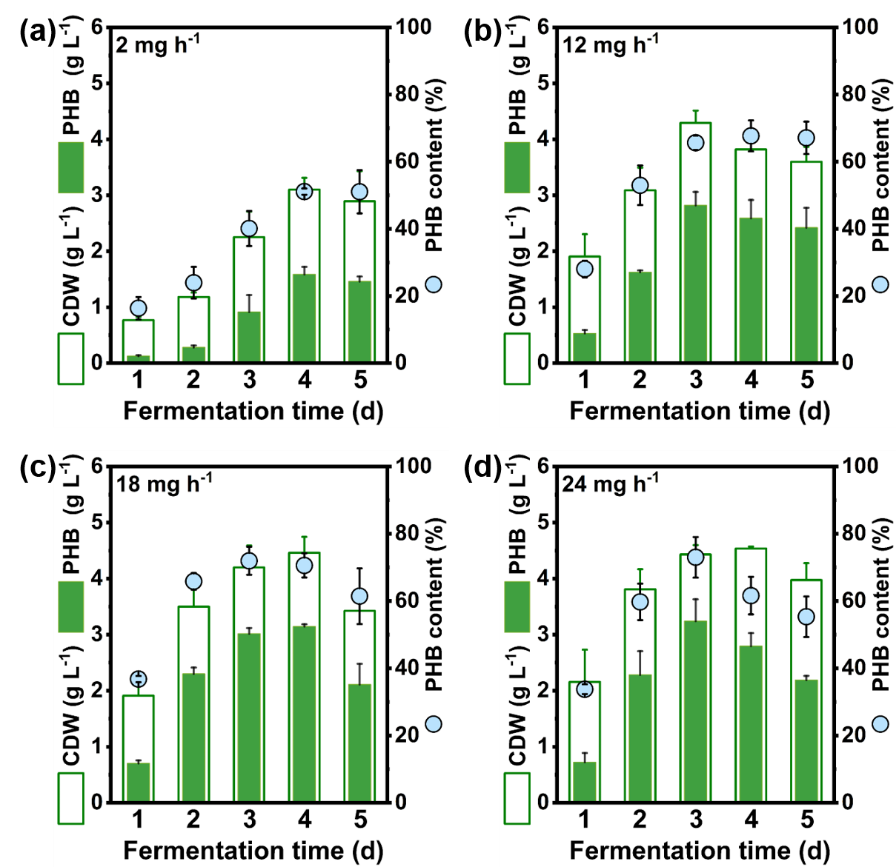


**Supplementary Fig. S45.** The non-sterile PHB production in a 1-L bioreactor by controlling the feeding amount of (NH_4_)_2_SO_4_. **a,** 2 mg h^−1^. **b,** 12 mg h^−1^. **c,** 18 mg h^−1^. **d,** 24 mg h^−1^.


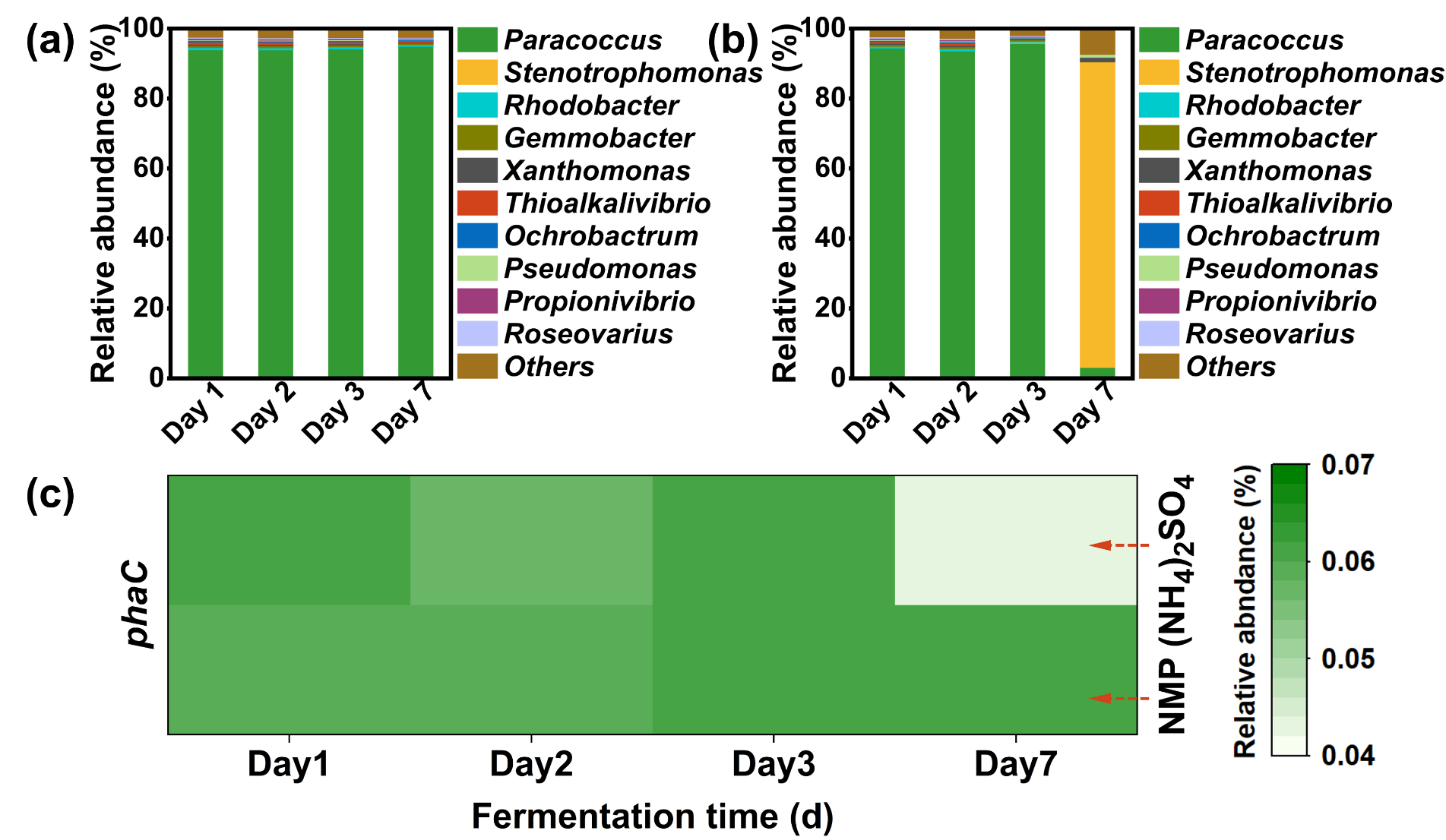


**Supplementary Fig. S46.** The microbial community dynamics of NMP and (NH_4_)_2_SO_4_-driven PHB fermentation systems under non-sterile conditions. **a,** NMP-driven PHB fermentation system. **b,** (NH_4_)_2_SO_4_-driven PHB fermentation system. **c,** The abundance changes of *phaC* in NMP and (NH_4_)_2_SO_4_-driven PHB fermentation systems.


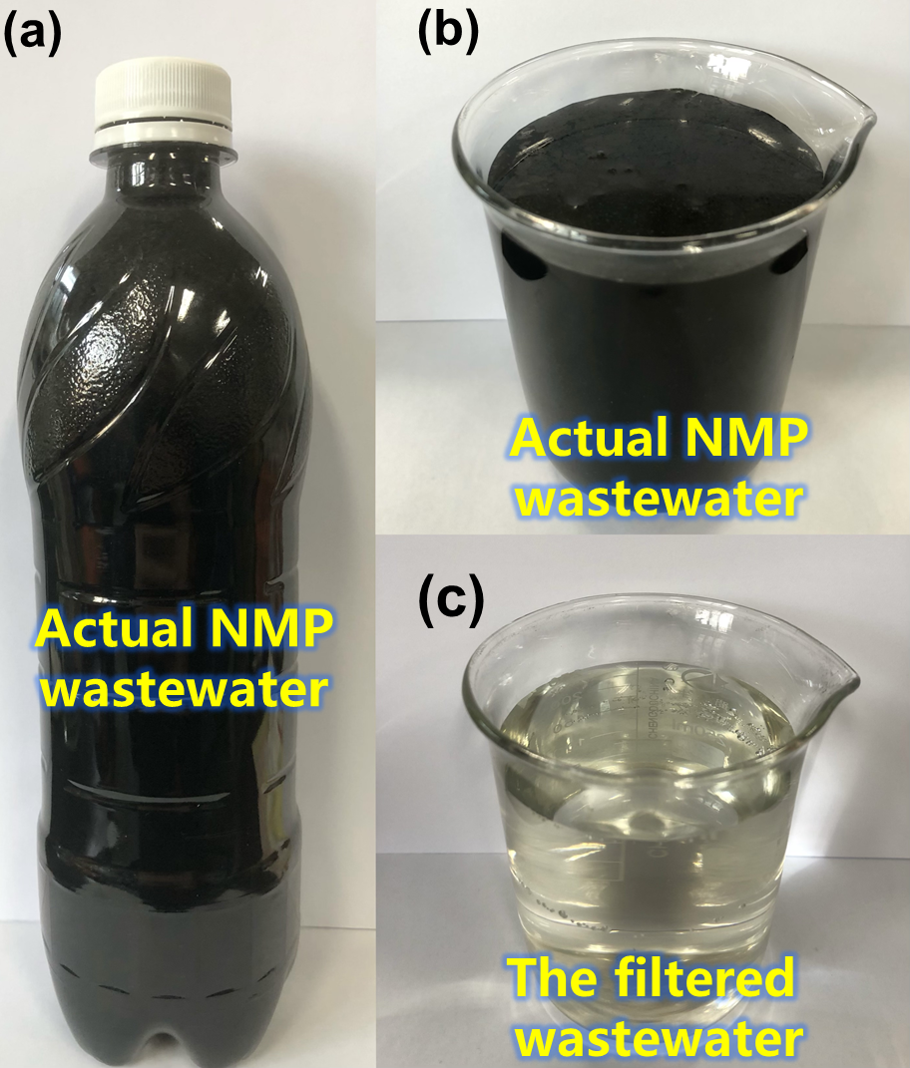


**Supplementary Fig. S47.** The collected NMP wastewater and the filtered wastewater.


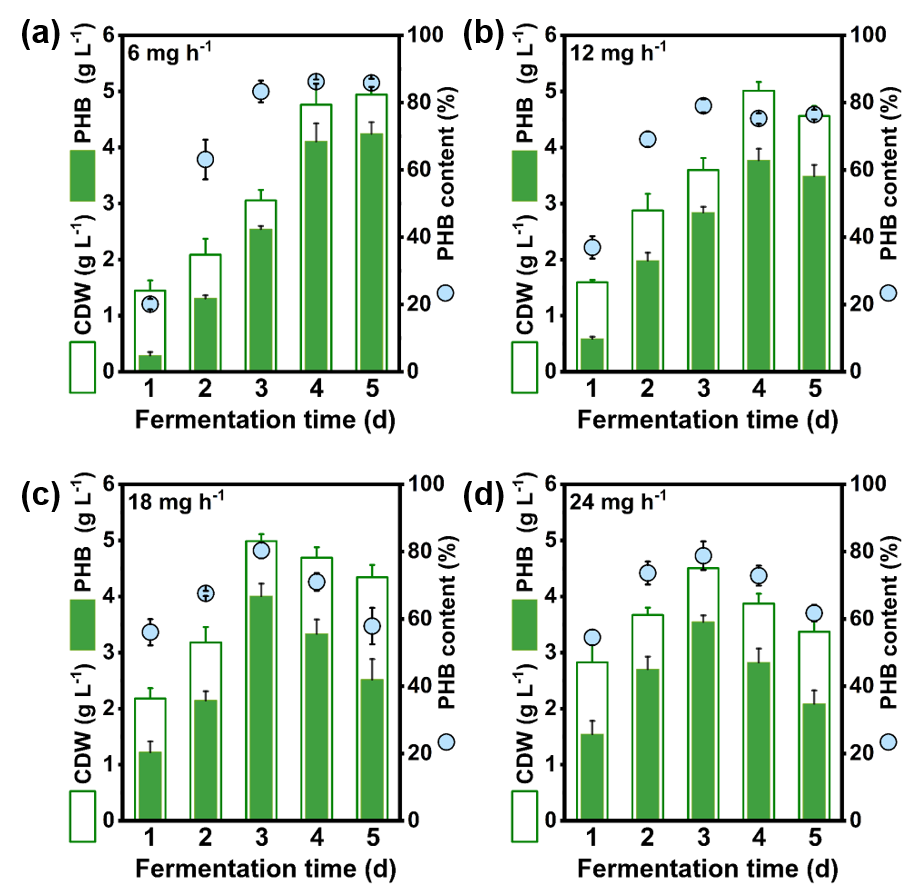


**Supplementary Fig. S48.** The semi-continuous PHB production from actual NMP wastewater in a 1-L bioreactor by controlling the feeding amount of NMP. **a,** 6 mg h^−1^. **b,** 12 mg h^−1^. **c,** 18 mg h^−1^. **d,** 24 mg h^−1^.


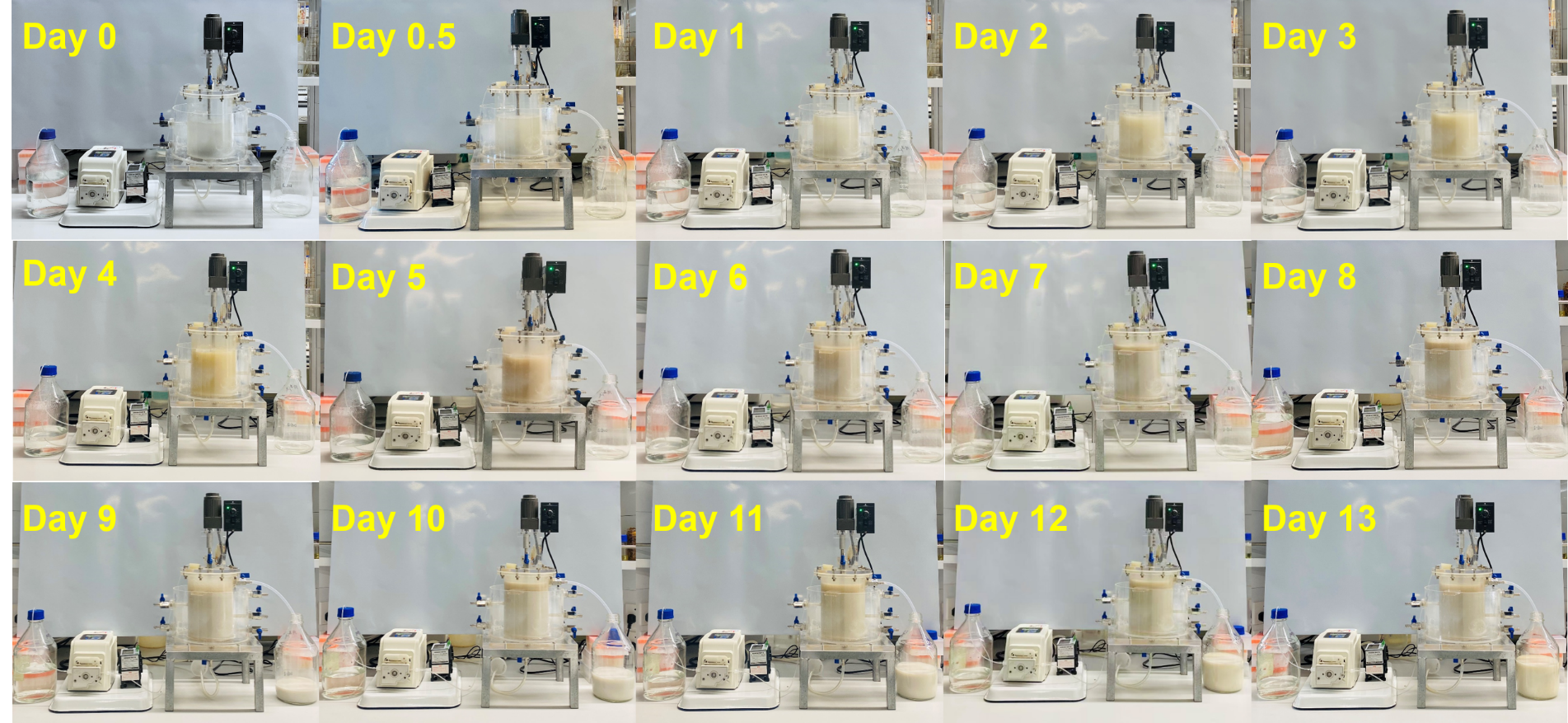


**Supplementary Fig. S49.** The continuous PHB production from filtered industrial NMP wastewater using *Paracoccus* sp. ZQW-1 as a “sustainable cell factory” under non-sterile conditions. ([sucrose] = 20 g L^−1^, [NMP] = 5 g L^−1^, [pH] = 8, [wastewater input] = 120 ml d^−1^).


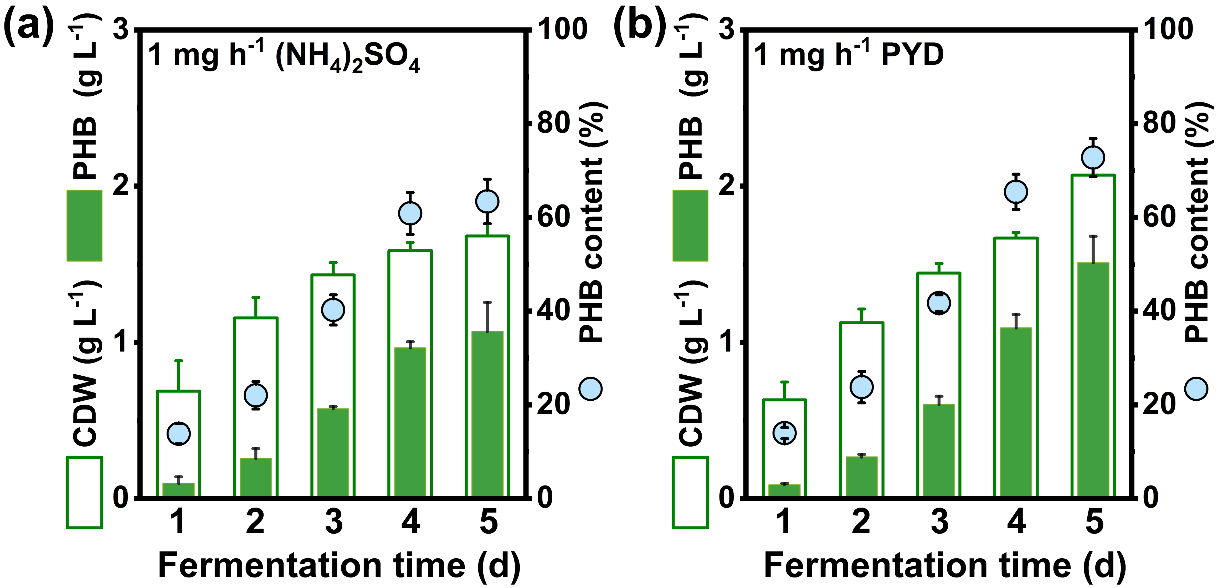


**Supplementary Fig. S50.** The semi-continuous PHB production by *Paracoccus* sp. ZQW-2 using simulated PYD wastewater and (NH_4_)_2_SO_4_ as nitrogen sources under non-sterile conditions. ([glycerol] = 5 g L^−1^, [pH] = 7, [inoculum concentration] = 6%, [temperature] = 30 °C, [nitrogen source] = 1 mg h^−1^).


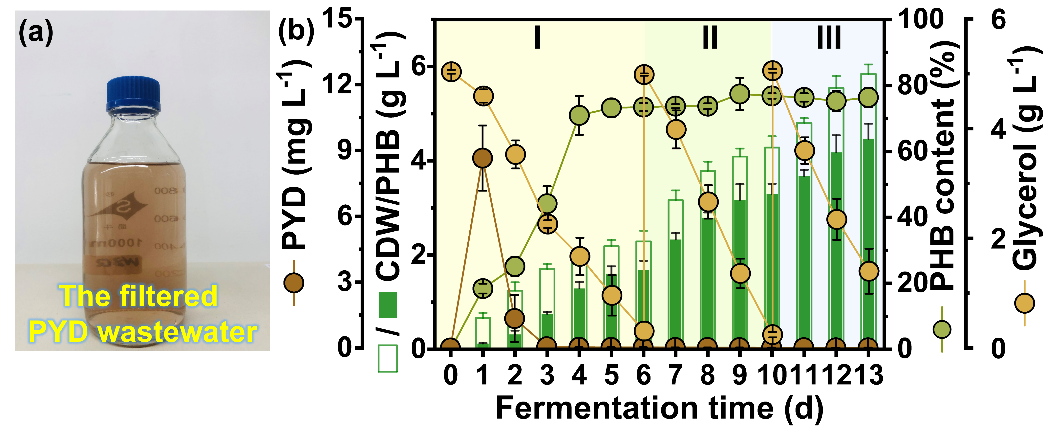


**Supplementary Fig. S51.** The continuous PHB production from actual PYD wastewater by *Paracoccus* sp. ZQW-2 under non-sterile conditions. **a,** The filtered PYD wastewater. **b,** Continuous PHB synthesis by *Paracoccus* sp. ZQW-2 using actual PYD wastewater under non-sterile conditions. ([glycerol] = 5 g L^−1^, [inoculum concentration] = 6%, [PYD] = 1 g L^−1^, [pH] = 9, [wastewater input] = 120 ml d^−1^).


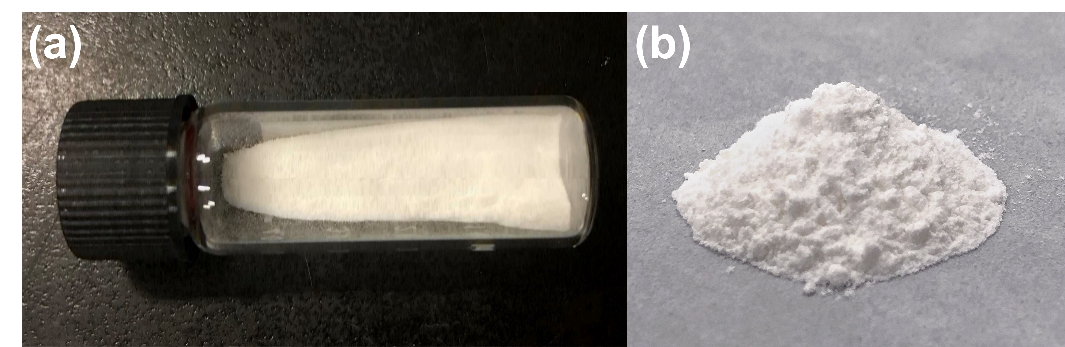


**Supplementary Fig. S52.** The extracted PHB from *Paracoccus* sp. ZQW-1.


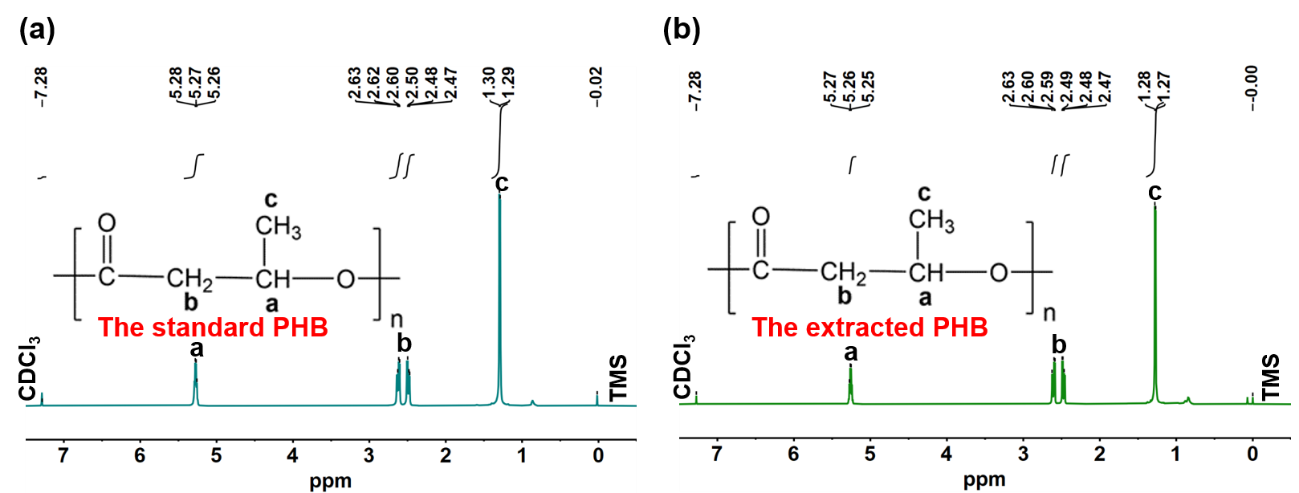


**Supplementary Fig. S53.** The comparison between standard PHB and wastewater-derived PHB by ^1^H-NMR spectra. **a,** Standard PHB (sigma). **b,** Wastewater-derived PHB.


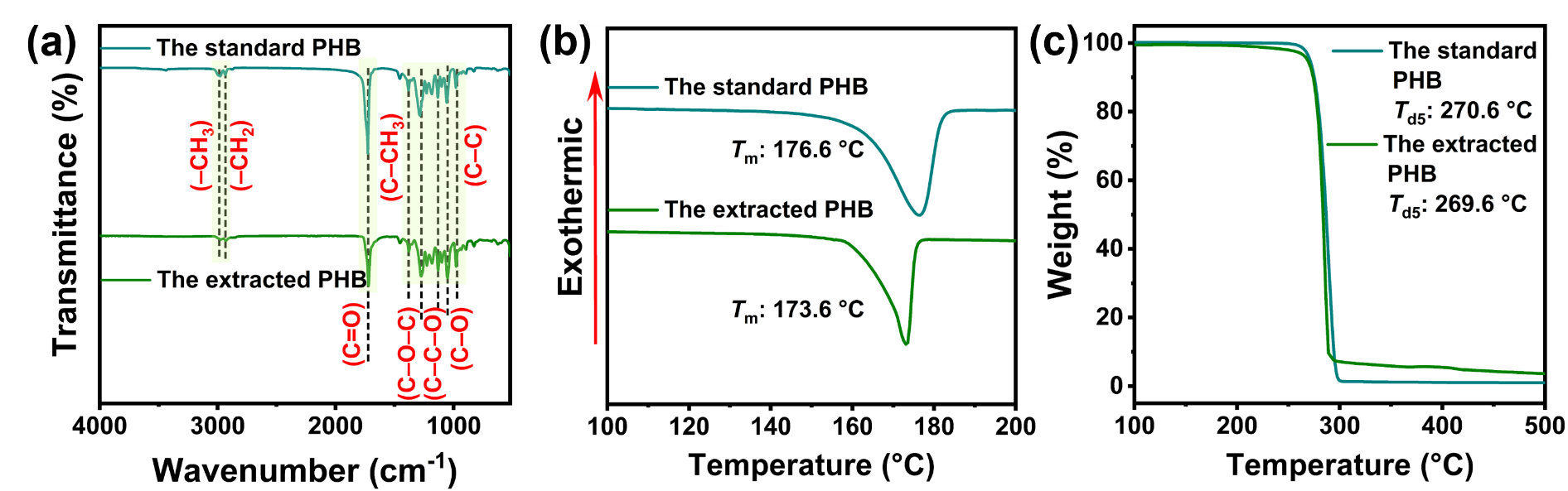


**Supplementary Fig. S54.** The comparison between standard PHB and wastewater-derived PHB by FT-IR spectrum, DSC analysis, and TGA analysis. **a,** FT-IR spectrum. **b,** DSC analysis. **c,** TGA analysis.


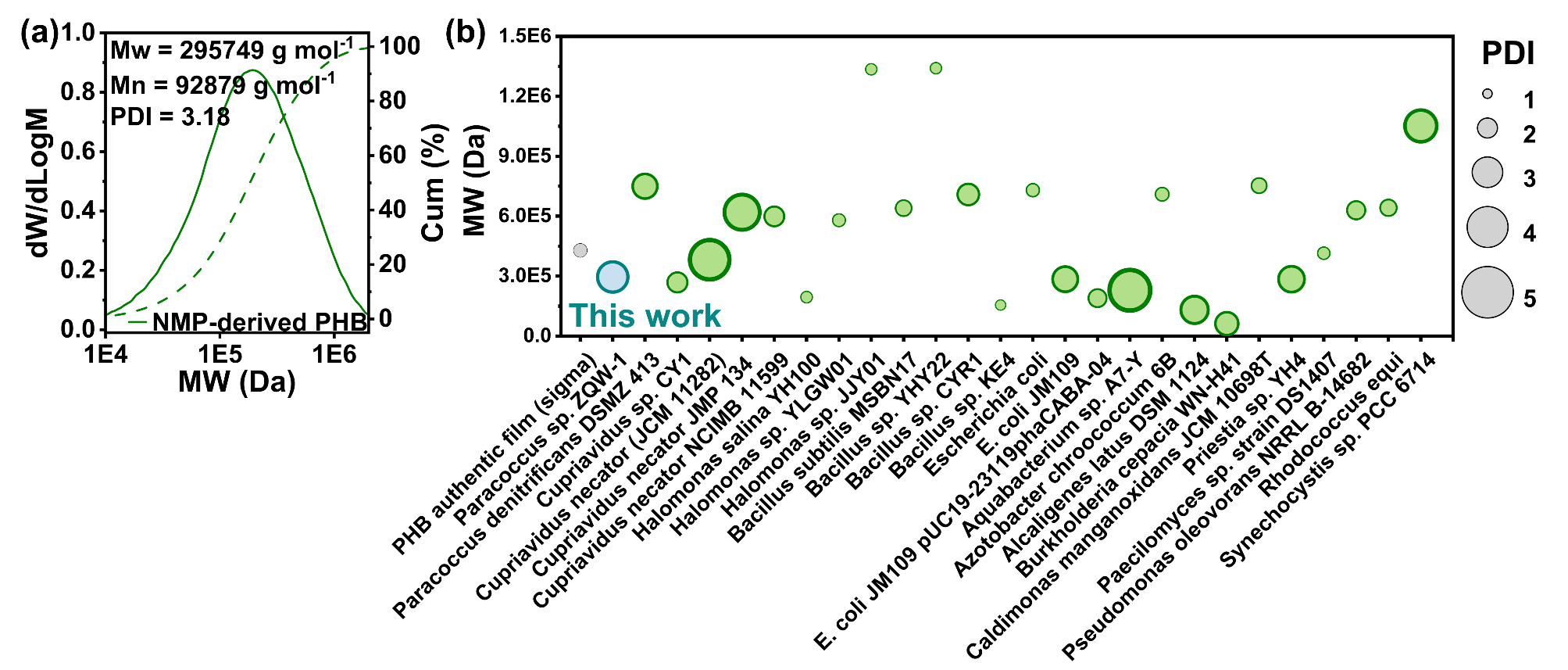


**Supplementary Fig. S55.** The molecular weight distribution of wastewater-derived PHB and the comparison with other documented PHB polymers. (Molecular weight (MW), weight average molecular weight (Mw), number average molecular weight (Mn), polydispersity index (PDI), cumulative molar mass distribution (Cum)).


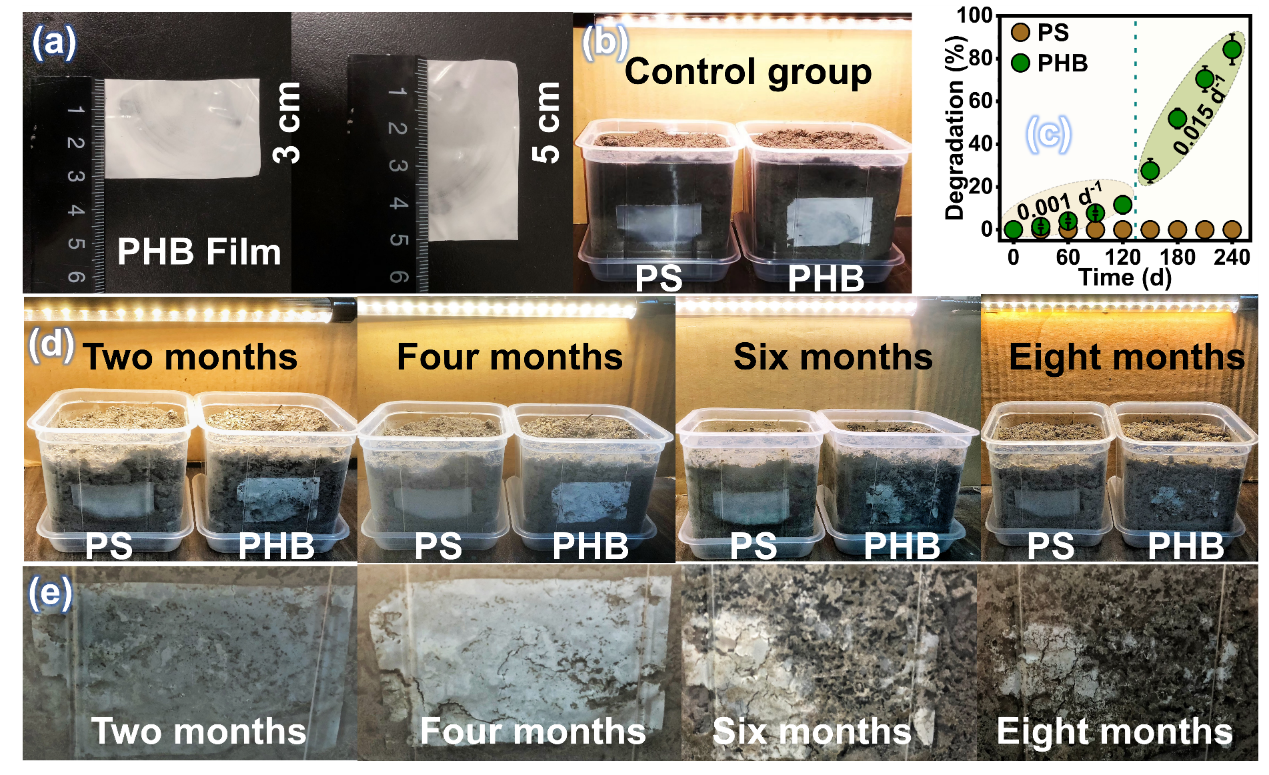


**Supplementary Fig. S56.** The degradation of PHB film and polystyrene (PS) film in soil and the morphological changes. **a,** PHB film (3 cm×5 cm). **b,** The self-assembled PHB degradation system. **c,** PHB degradation during 240 days. **d, e,** The morphological changes of PHB films during degradation process.


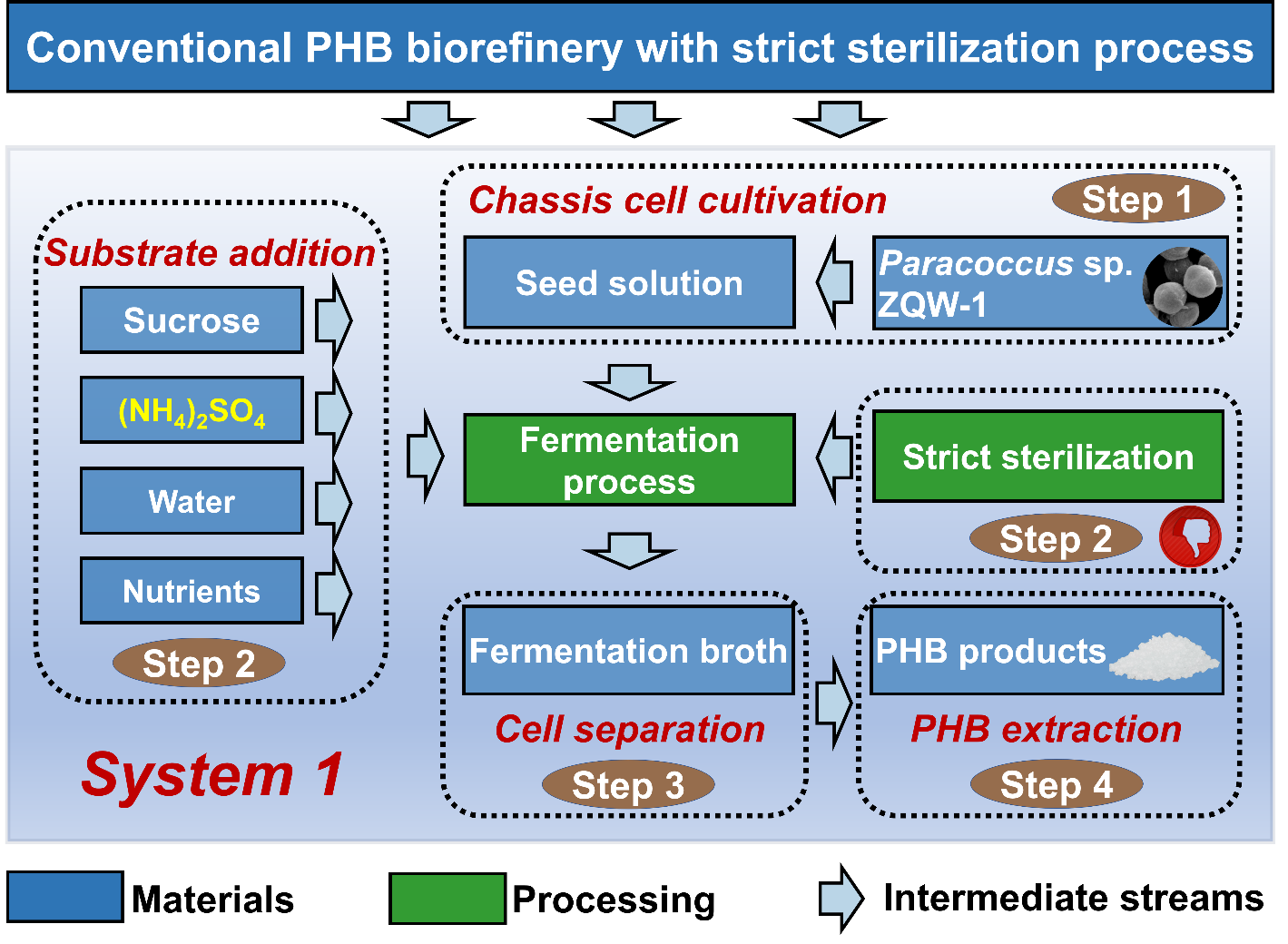


**Supplementary Fig. S57.** The system boundary of conventional PHB production under sterile conditions.


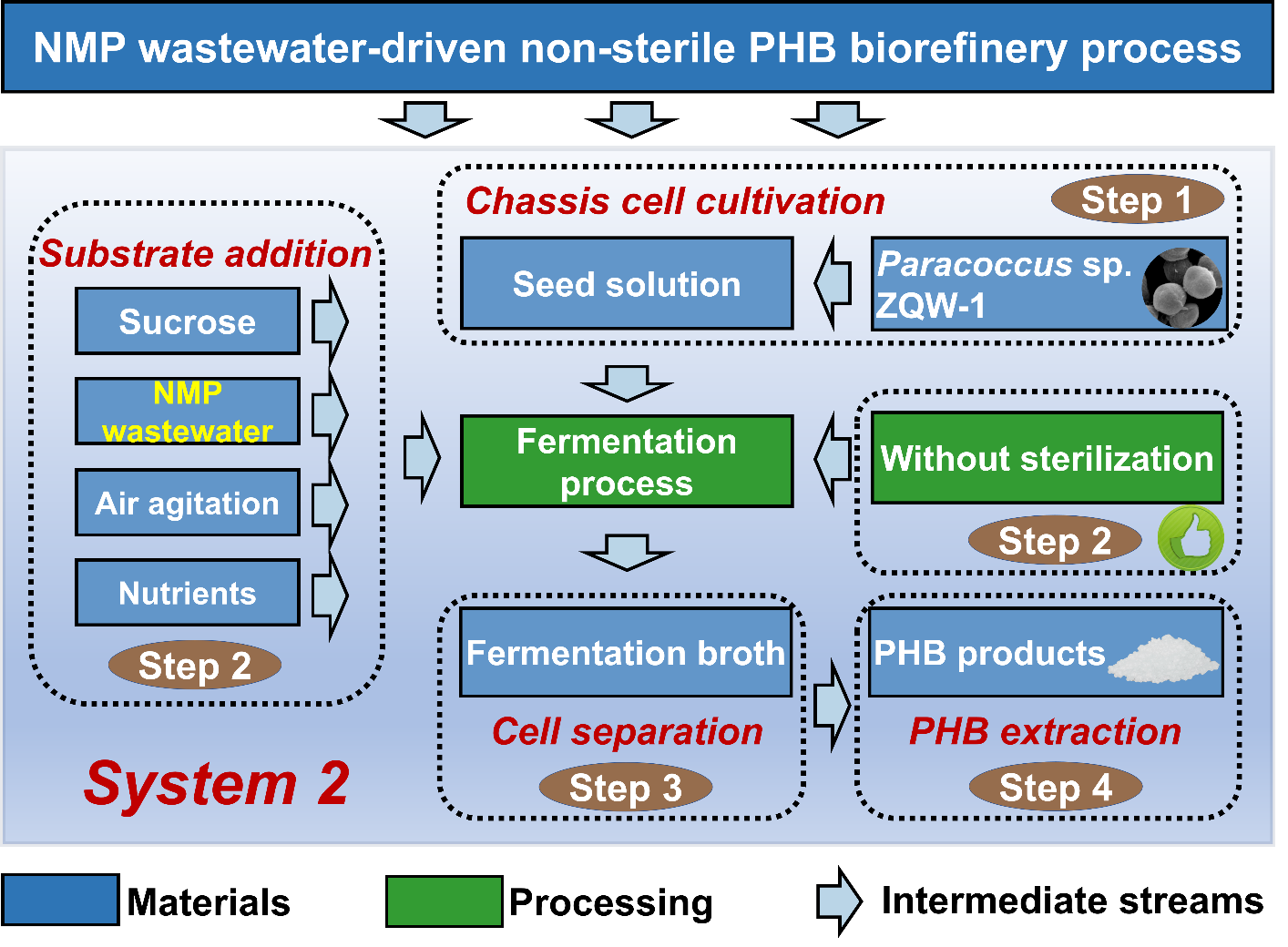


**Supplementary Fig. S58.** The system boundary of wastewater-driven PHB production under non-sterile conditions.


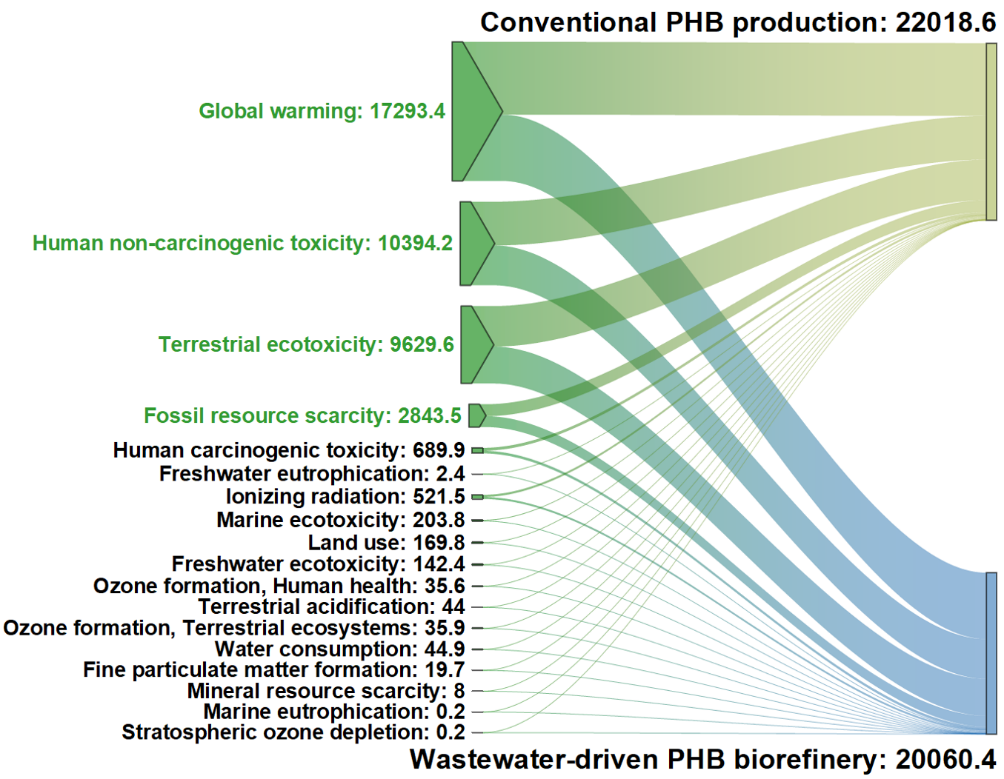


**Supplementary Fig. S59.** The sankey flow diagram of 18 midpoint indicators to show their contribution for PHB production.


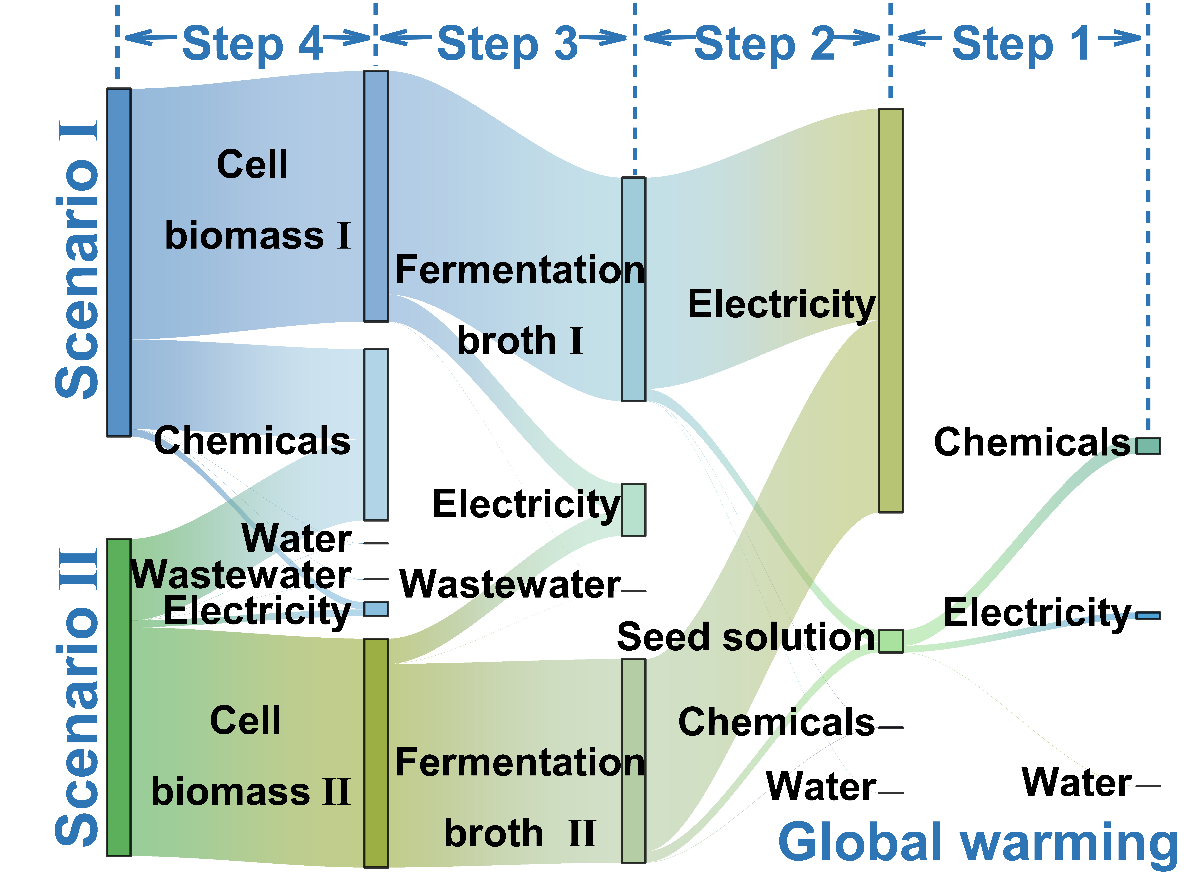


**Supplementary Fig. S60.** The sankey flow diagram of the contribution of each production step for global warming.


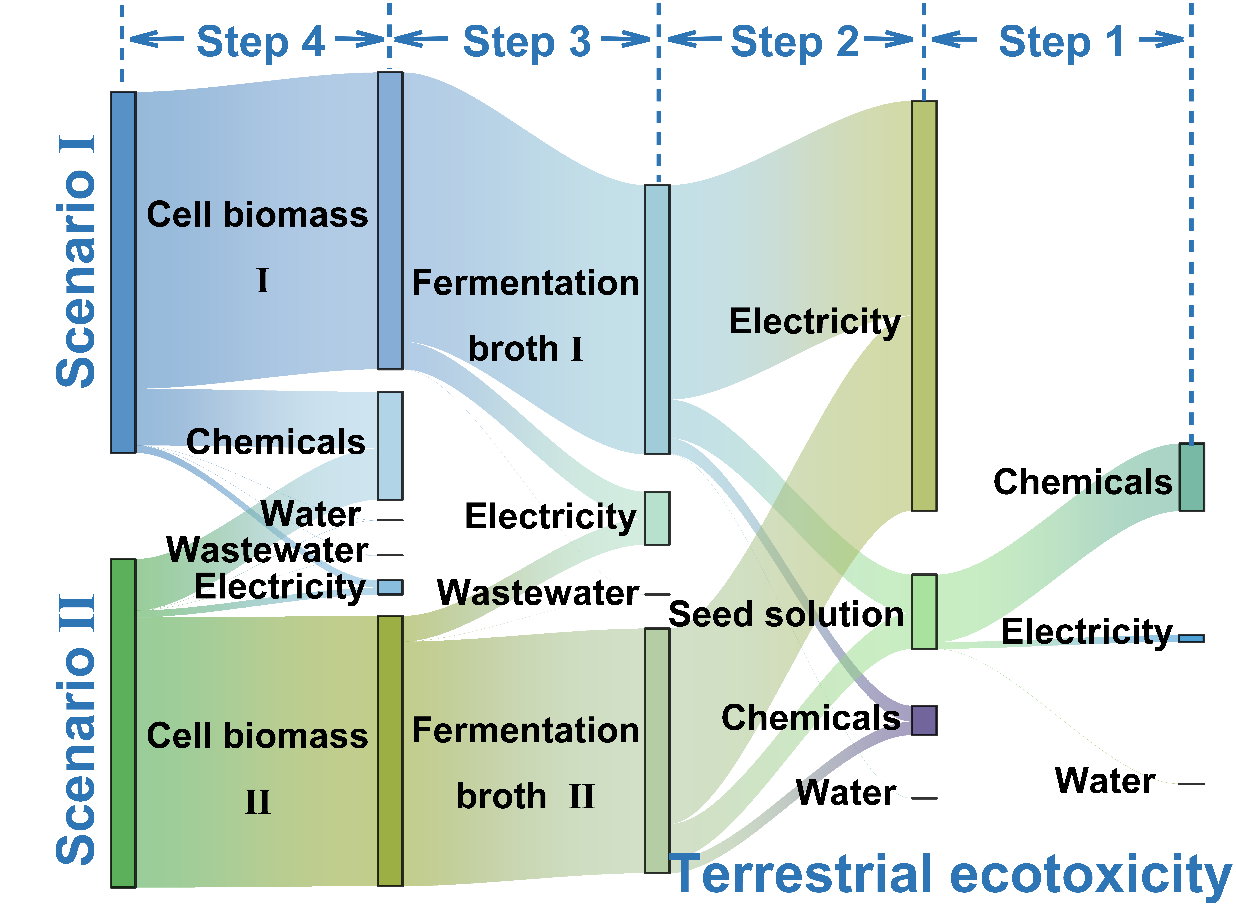


**Supplementary Fig. S61.** The sankey flow diagram of the contribution of each production step for terrestrial ecotoxicity.


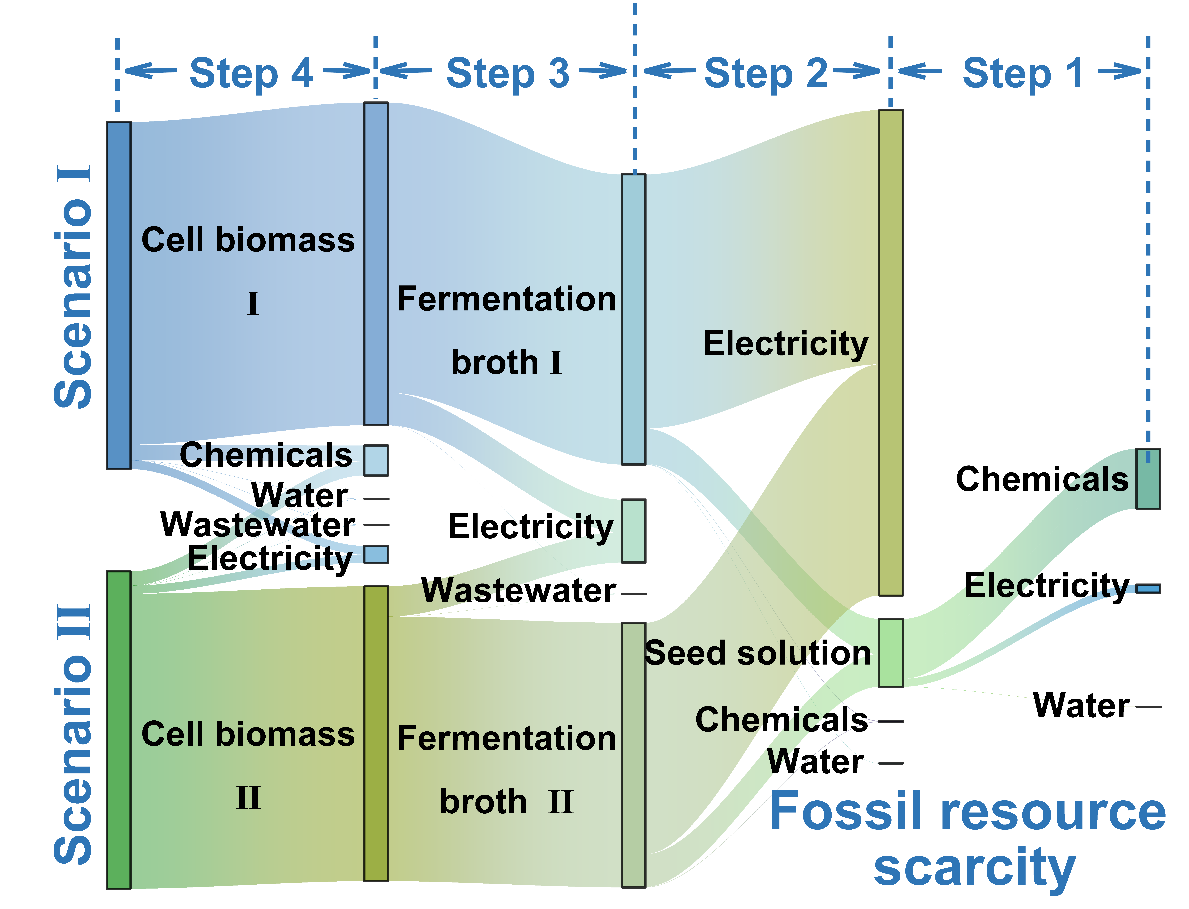


**Supplementary Fig. S62.** The sankey flow diagram of the contribution of each production step for fossil resource scarcity.


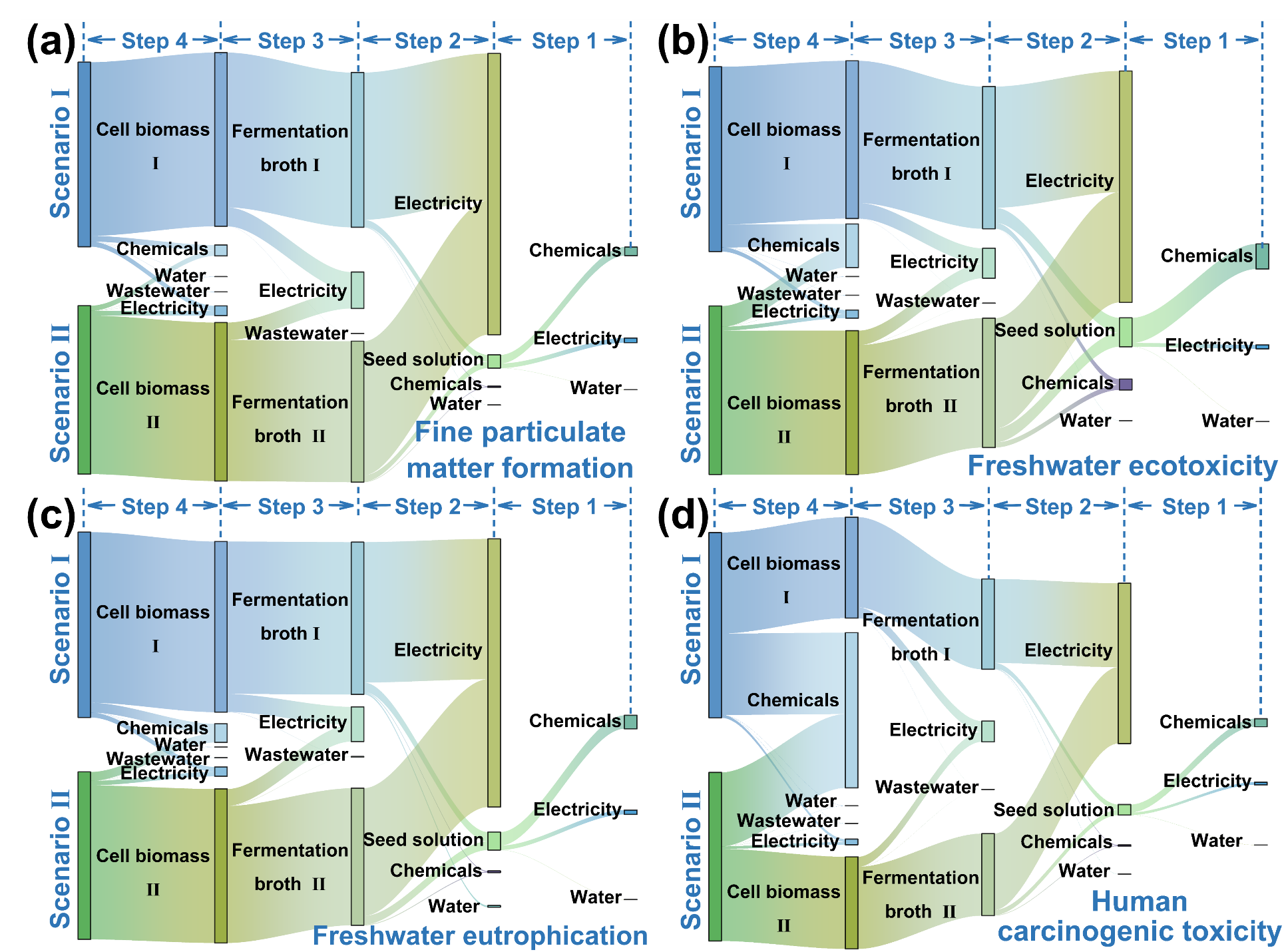


**Supplementary Fig. S63.** The sankey flow diagram of the contribution of each production step for fine particulate matter formation, freshwater ecotoxicity, freshwater eutrophication, and human carcinogenic toxicity.


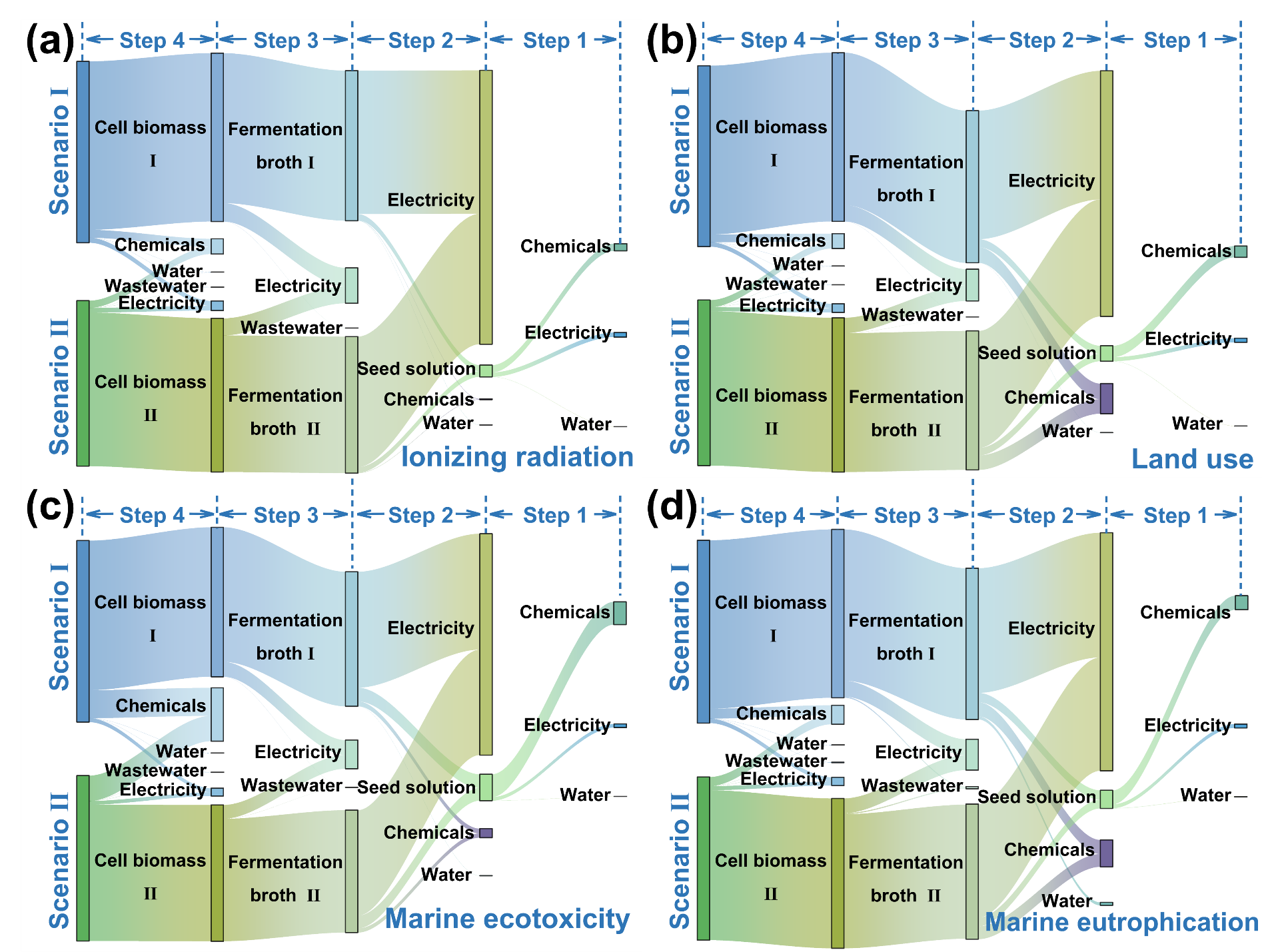


**Supplementary Fig. S64.** The sankey flow diagram of the contribution of each production step for ionizing radiation, land use, marine ecotoxicity, and marine eutrophication.


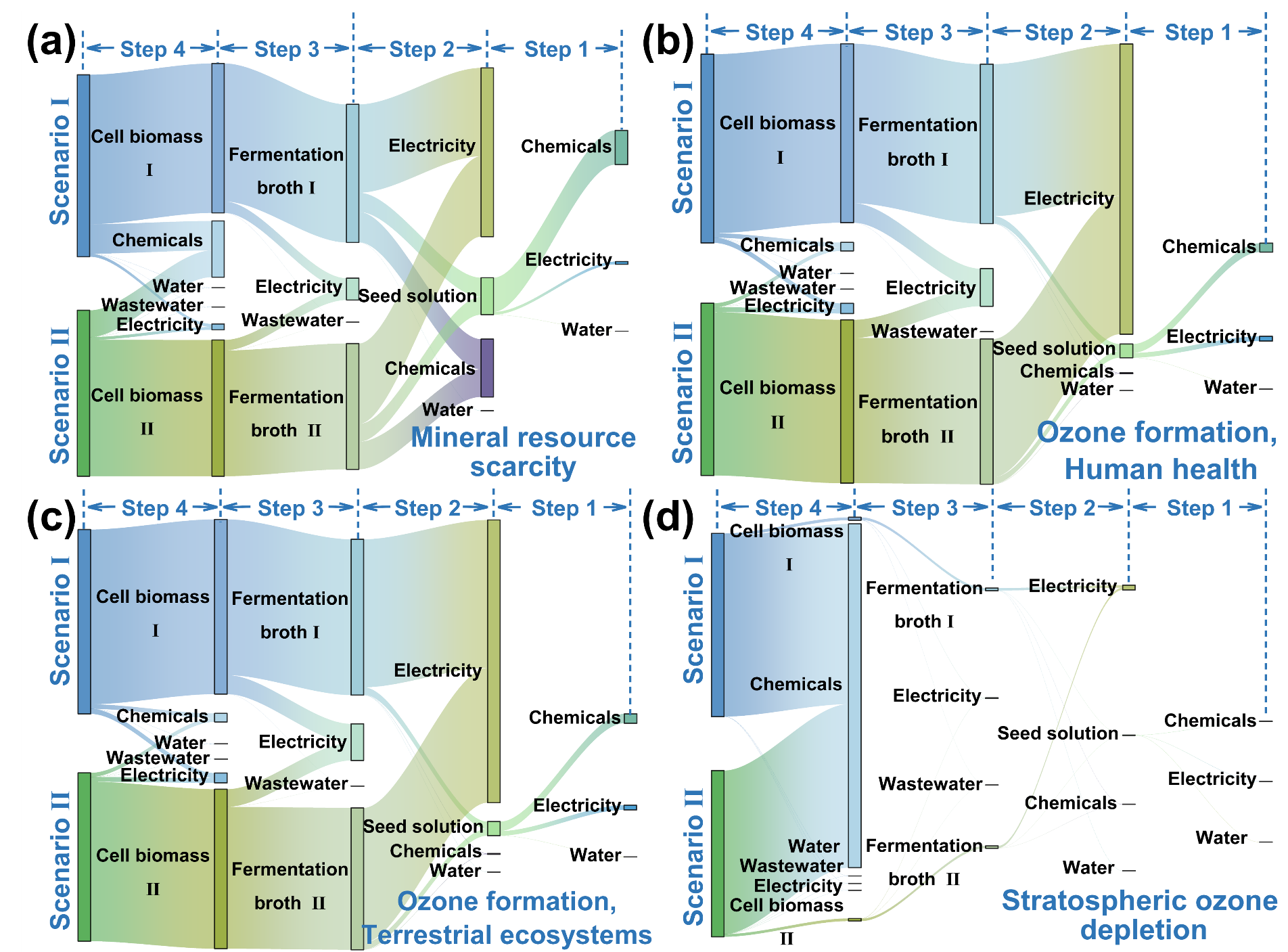


**Supplementary Fig. S65.** The sankey flow diagram of the contribution of each production step for mineral resource scarcity, ozone formation, human health, ozone formation, terrestrial ecosystems, stratospheric ozone depletion.


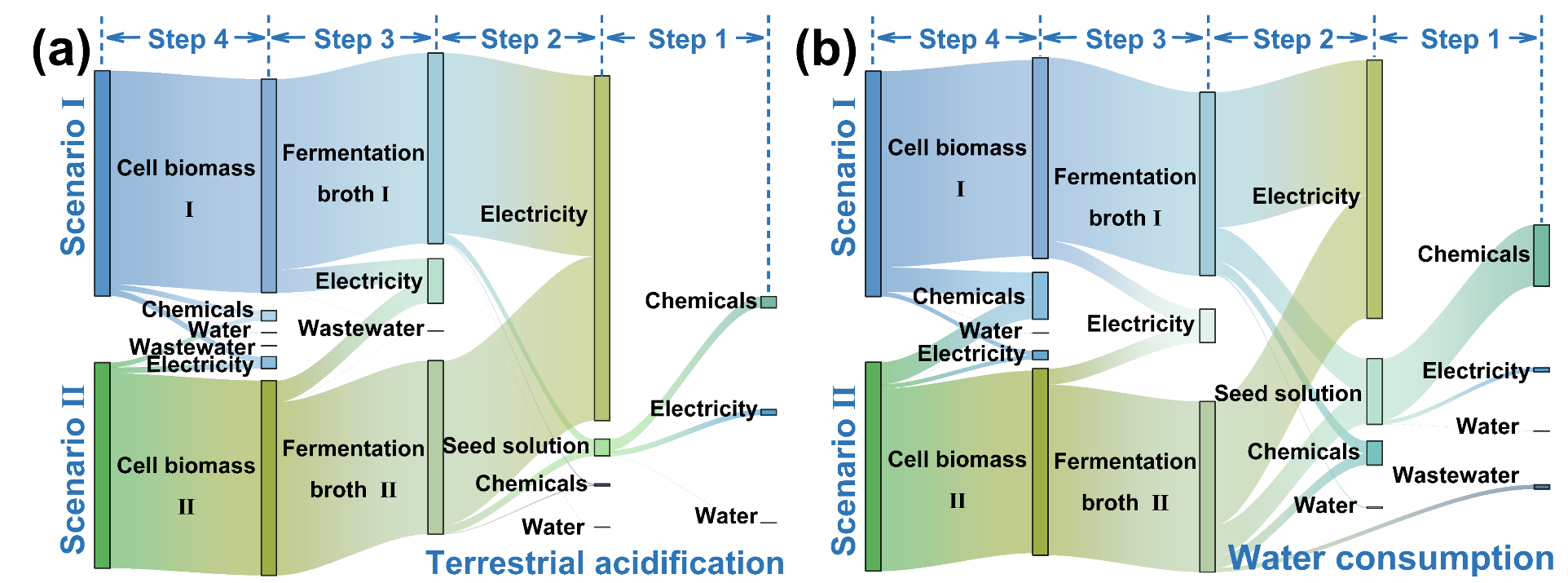


**Supplementary Fig. S66.** The sankey flow diagram of the contribution of each production step for terrestrial acidification and water consumption.

**Supplementary Table S1.** The basic information of NMP wastewater collected from a New Materials Technology Co., Ltd. in ZhenJiang, China.

| **Water sample** | **pH ^a^** | **COD_Cr_ (mg L^-1^) ^b^** | **NMP (g L^-1^) ^c^** | **NH_3_-N (mg L^-1^) ^d^** |
| --- | --- | --- | --- | --- |
| NMP wastewater | 7. 0± 0.02 | 48755 ± 671 | 33.2 ± 0.57 | 3.36 ± 0.13 |

**^a^** The solution pH was detected by a PHS-3C pH meter (INESA).

**^b^** The COD_cr_ was measured based on the standard analytical methods for the wastewater issued by the State Environmental Protection Administration (SEPA), China.

**^c^** The concentration of NMP was detected by HPLC.

**^d^** The concentration of NH_3_-N was detected by Nessler’s reagent colorimetric method.

**Supplementary Table S2.** The biodegradation of NMP by *Paracoccus* sp. ZQW-1 and other documented bacterial strains.

| **NMP degrading strain** | **NMP concentration (g L^-1^)** | **Degradation efficiency (%)** | **Reaction time (h)** | **Reference** |
| --- | --- | --- | --- | --- |
| ***Paracoccus* sp. ZQW-1** | **2.0** | **100%** | **36** | **This work** |
| *Paracoccus* sp. NMD-4 | 0.5 | 100% | 24 | ^[^[^4^](#_ENREF_4)^]^ |
| *Paracoccus pantotrophus* NJUST38 | 0.5 | 100% | 11 | ^[^[^5^](#_ENREF_5)^]^ |
| *Paracoccus* sp. MP1 | 0.3 | 92.45 ± 2.24% | 72 | ^[^[^6^](#_ENREF_6)^]^ |
| *Bacillus* sp. NMP-2 | 0.5 | 100% | 36 | ^[^[^7^](#_ENREF_7)^]^ |
| *Bacillus* pumilus NJUST39 | 1.2 | 100% | 23 | ^[^[^8^](#_ENREF_8)^]^ |
| *M. organophilum* | 0.3 | 100% | 5 | ^[^[^9^](#_ENREF_9)^]^ |
| *S. melonis* | 0.3 | 100% | 8 | ^[^[^9^](#_ENREF_9)^]^ |
| *Pseudomonas* sp. MP10 | 0.3 | 79.21 ± 0.91% | 72 | ^[^[^6^](#_ENREF_6)^]^ |
| *Pseudomonas* sp. MP11 | 0.3 | 78.29 ± 1.99% | 72 | ^[^[^6^](#_ENREF_6)^]^ |
| *Acinetobacter* sp. MP12 | 0.3 | 74.38 ± 1.36% | 72 | ^[^[^6^](#_ENREF_6)^]^ |
| *Rhodococcus* sp. MP2 | 0.3 | 78.65 ± 1.06% | 72 | ^[^[^6^](#_ENREF_6)^]^ |

**Supplementary Table S3.** The functional genes related to NMP biodegradation.

| **Sequence name** | **Sequence description** | **Sequence length** | **COG ID** |
| --- | --- | --- | --- |
| Gene1813 | N-methylhydantoin amidohydrolase A (*nmpA*) | 2031 bp | COG0145 |
| Gene1814 | N-methylhydantoin amidohydrolase B (*nmpB*) | 1764 bp | COG0146 |
| Gene1815 | Amino acid oxidase (*nmpC*) | 1404 bp | COG0665 |
| Gene1816 | Cupin domain-containing protein (*nmpD)* | 339 bp | COG3450 |
| Gene1817 | PucR family transcriptional regulator (*nmpE*) | 1542 bp | COG2508 |
| Gene1818 | Succinic-semialdehyde dehydrogenase (*nmpF*) | 1449 bp | COG1012 |

**Supplementary Table S4.** The production of PHA by different carbon sources (Fermentation conditions: carbon sources: 10 g L^−1^, C/N ratio: 40:1, temperature: 30 ℃, fermentation time: 2 d, inoculation concentration: 4 %).

| **Carbon sources** | **CDW (g L^−1^)** | **PHA content (%)** | **PHA concentration (g** **L^−1^)** |
| --- | --- | --- | --- |
| Glucose | 3.24 ± 0.30 | 45.96 ± 2.33 | 1.49 ± 0.21 |
| Fructose | 3.43 ± 0.26 | 53.38 ± 0.91 | 1.83 ± 0.17 |
| **Sucrose** | **4.18 ± 0.17** | **69.81 ± 0.68** | **2.92 ± 0.15** |
| Maltose | 3.16 ± 0.30 | 51.87 ± 0.99 | 1.64 ± 0.19 |
| Lactose | 0.88 ± 0.07 | 0.00 | 0.00 |
| Xylose | 0.68 ± 0.13 | 0.00 | 0.00 |
| Galactose | 2.76 ± 0.36 | 33.05 ± 7.32 | 0.92 ± 0.32 |
| Sodium citrate | 0.59 ± 0.34 | 0.00 | 0.00 |
| Cellulose | 0.72 ± 0.11 | 0.00 | 0.00 |
| Lignin | 0.84 ± 0.15 | 0.00 | 0.00 |
| Glycerin | 2.33 ± 0.43 | 26.98 ± 2.65 | 0.63 ± 0.18 |
| Butyric acid | 3.07 ± 0.36 | 40.34 ± 2.24 | 1.24 ± 0.21 |
| Acetic acid | 2.13 ± 0.33 | 63.63 ± 1.14 | 1.36 ± 0.23 |
| Propionic acid | 2.33 ± 0.36 | 23.70 ± 5.61 | 0.56 ± 0.22 |
| Valeric acid | 2.98 ± 0.35 | 45.34 ± 2.87 | 1.35 ± 0.25 |
| Mannitol | 3.36 ± 0.34 | 50.48 ± 2.58 | 1.70 ± 0.26 |
| Ethanol | 1.43 ± 0.04 | 15.03 ± 0.33 | 0.21 ± 0.01 |
| Methanol | 1.69 ± 0.22 | 17.55 ± 1.89 | 0.30 ± 0.01 |
| Soluble starch | 2.21 ± 0.42 | 10.92 ± 4.81 | 0.25 ± 0.15 |

**Supplementary Table S5.** The optimized PHB production by using sucrose as the carbon source (Fermentation conditions: carbon sources: 5−25 g L^−1^, C/N ratio: 5−100, temperature: 30 ℃, fermentation time: 2 d, inoculation concentration: 4 %).

| **Sucrose concentration (g L^−1^)** | **C/N ratio** | **CDW**  **(g L^−1^)** | **PHA content (%)** | **PHA concentration (g L^−1^)** |
| --- | --- | --- | --- | --- |
| 5 | 5 | 3.63 ± 0.13 | 16.55 ± 1.50 | 0.60 ± 0.08 |
| 10 | 5 | 4.13 ±0.07 | 19.09 ± 1.84 | 0.79 ± 0.06 |
| 15 | 5 | 4.08 ±0.22 | 23.68 ± 2.06 | 0.96 ± 0.03 |
| 20 | 5 | 4.14 ±0.02 | 22.85 ± 1.36 | 0.95 ± 0.05 |
| 25 | 5 | 4.19 ±0.11 | 23.83 ± 0.93 | 0.99 ± 0.07 |
| 5 | 10 | 2.45 ±0.30 | 18.60 ± 4.99 | 0.46 ± 0.18 |
| 10 | 10 | 4.19 ±0.09 | 20.73 ± 2.97 | 0.87 ± 0.14 |
| 15 | 10 | 4.28 ±0.33 | 20.70 ± 3.67 | 0.89 ± 0.22 |
| 20 | 10 | 4.44 ±0.15 | 26.23 ± 3.88 | 1.17 ± 0.21 |
| 25 | 10 | 4.39 ±0.35 | 24.31 ± 0.62 | 1.07 ± 0.11 |
| 5 | 20 | 2.36 ±0.19 | 23.98 ± 4.25 | 0.57 ± 0.14 |
| 10 | 20 | 4.21 ±0.04 | 30.93 ± 3.65 | 1.30 ± 0.14 |
| 15 | 20 | 4.25 ±0.27 | 30.70 ± 2.28 | 1.31 ± 0.18 |
| 20 | 20 | 4.57 ±0.24 | 24.97 ± 0.53 | 1.14 ± 0.08 |
| 25 | 20 | 4.50 ±0.21 | 28.36 ± 1.34 | 1.28 ± 0.12 |
| 5 | 40 | 2.31 ±0.17 | 52.69 ± 2.89 | 1.22 ± 0.16 |
| 10 | 40 | 4.29 ±0.17 | 68.26 ± 1.95 | 2.93 ± 0.20 |
| 15 | 40 | 4.37 ±0.22 | 71.46 ± 2.47 | 3.12 ± 0.05 |
| **20** | **40** | **5.04 ±0.12** | **74.93 ± 0.45** | **3.77 ± 0.07** |
| 25 | 40 | 5.03 ±0.22 | 68.65 ± 1.11 | 3.45 ± 0.09 |
| 5 | 60 | 2.29 ±0.12 | 58.30 ± 2.54 | 1.34 ± 0.13 |
| 10 | 60 | 3.08 ±0.39 | 57.63 ± 0.81 | 1.77 ± 0.19 |
| 15 | 60 | 3.99 ±0.04 | 57.17 ± 3.82 | 2.28 ± 0.18 |
| 20 | 60 | 4.48 ±0.09 | 56.78 ± 1.41 | 2.55 ± 0.12 |
| 25 | 60 | 4.40 ±0.18 | 58.29 ± 3.07 | 2.56 ± 0.03 |
| 5 | 80 | 2.38 ±0.09 | 53.25 ± 3.63 | 1.27 ± 0.03 |
| 10 | 80 | 2.85 ±0.14 | 61.89 ± 0.97 | 1.76 ± 0.12 |
| 15 | 80 | 3.96 ±0.11 | 61.89 ± 1.21 | 2.45 ± 0.12 |
| 20 | 80 | 4.19 ±0.22 | 57.63 ± 0.83 | 2.42 ± 0.16 |
| 25 | 80 | 4.37 ±0.24 | 57.85 ± 5.15 | 2.52 ± 0.09 |
| 5 | 100 | 2.18 ±0.21 | 52.79 ± 2.88 | 1.15 ± 0.05 |
| 10 | 100 | 2.76 ±0.32 | 49.36 ± 6.17 | 1.35 ± 0.01 |
| 15 | 100 | 3.91 ±0.31 | 48.33 ± 3.28 | 1.88 ± 0.02 |
| 20 | 100 | 4.06 ±0.26 | 43.90 ± 1.67 | 1.78 ± 0.05 |
| 25 | 100 | 4.24 ±0.23 | 47.33 ± 2.31 | 2.01 ± 0.21 |

**Supplementary Table S6.** The effects of fermentation time on PHB production (Fermentation conditions: sucrose: 20 g L^−1^, C/N ratio: 40:1, temperature: 30 ℃, fermentation time: 1−7 d, inoculation concentration: 4 %).

| **Fermentation time (d)** | **CDW (g L^−1^)** | **PHA content (%)** | **PHA concentration (g L^−1^)** |
| --- | --- | --- | --- |
| 1 | 3.53 ± 0.05 | 24.04 ± 5.54 | 0.85 ± 0.21 |
| 2 | 5.01 ± 0.19 | 74.28 ± 0.45 | 3.72 ± 0.17 |
| **3** | **5.21 ± 0.15** | **80.51 ± 3.84** | **4.19 ± 0.08** |
| 4 | 5.41 ± 0.21 | 64.28 ± 4.82 | 3.48 ± 0.39 |
| 5 | 4.83 ± 0.19 | 63.48 ± 0.49 | 3.07 ± 0.09 |
| 6 | 4.69 ± 0.11 | 61.36 ± 4.79 | 2.88 ± 0.15 |
| 7 | 4.64 ± 0.18 | 57.94 ± 3.07 | 2.69 ± 0.24 |

**Supplementary Table S7.** The effects of inoculation dosage on PHB production (Fermentation conditions: sucrose: 20 g L^−1^, C/N ratio: 40:1, temperature: 30 ℃, fermentation time: 3 d, inoculation concentration: 2−14 %).

| **Inoculation dosage (%)** | **CDW (g L^−1^)** | **PHA content (%)** | **PHA concentration (g L^−1^)** |
| --- | --- | --- | --- |
| 2 | 4.32 ± 0.29 | 55.82 ± 2.62 | 2.41 ± 0.28 |
| **4** | **5.01 ± 0.31** | **79.23 ± 1.56** | **3.97 ± 0.32** |
| 6 | 4.96 ± 0.11 | 77.84 ± 2.22 | 3.86 ± 0.20 |
| 8 | 4.91 ± 0.09 | 71.66 ± 3.13 | 3.52 ± 0.22 |
| 10 | 4.99 ± 0.27 | 72.99 ± 5.14 | 3.64 ± 0.06 |
| 12 | 4.95 ± 0.27 | 74.13 ± 3.78 | 3.67 ± 0.38 |
| 14 | 4.83 ± 0.07 | 66.47 ± 2.44 | 3.21 ± 0.07 |

**Supplementary Table S8.** The effects of solution pH on PHB production (Fermentation conditions: sucrose: 20 g L^−1^, C/N ratio: 40:1, temperature: 30 ℃, fermentation time: 3 d, inoculation concentration: 4 %, pH 5−11).

| **pH** | **CDW (g L^−1^)** | **PHA content (%)** | **PHA concentration (g L^−1^)** |
| --- | --- | --- | --- |
| 5 | 0.00 | 0.00 | 0.00 |
| 6 | 0.00 | 0.00 | 0.00 |
| 7 | 4.87 ± 0.03 | 75.16 ± 3.93 | 3.66 ± 0.17 |
| **8** | **5.02 ± 0.29** | **79.41 ± 2.61** | **3.99 ± 0.36** |
| 9 | 4.58 ± 0.12 | 62.80 ± 3.97 | 2.88 ± 0.26 |
| 10 | 0.00 | 0.00 | 0.00 |
| 11 | 0.00 | 0.00 | 0.00 |

**Supplementary Table S9.** The effects of NaCl concentration on PHB production (Fermentation conditions: sucrose: 20 g L^−1^, C/N ratio: 40:1, temperature: 30 ℃, fermentation time: 3 d, inoculation concentration: 4 %, pH 8, NaCl: 5−70 g L^−1^).

| **NaCl (g L^−1^)** | **CDW (g L^−1^)** | **PHA content (%)** | **PHA concentration (g L^−1^)** |
| --- | --- | --- | --- |
| **0** | **5.02 ± 0.06** | **80.12 ± 2.93** | **4.02 ± 0.20** |
| 10 | 4.48 ± 0.20 | 68.98 ± 5.59 | 3.01 ± 0.11 |
| 20 | 4.17 ± 0.26 | 33.81 ± 4.03 | 1.41 ± 0.25 |
| 30 | 3.94 ± 0.19 | 22.36 ± 4.31 | 0.88 ± 0.13 |
| 40 | 1.33 ± 0.17 | 5.95 ± 1.05 | 0.08 ± 0.01 |
| 50 | 0.00 | 0.00 | 0.00 |
| 60 | 0.00 | 0.00 | 0.00 |
| 70 | 0.00 | 0.00 | 0.00 |

**Supplementary Table S10.** The effects of fermentation temperature on PHB production (Fermentation conditions: sucrose: 20 g L^−1^, C/N ratio: 40:1, temperature: 15−45 ℃, fermentation time: 3 d, inoculation concentration: 4 %, pH 8, NaCl: 5 g L^−1^).

| **Temperature (℃)** | **CDW (g L^−1^)** | **PHA content (%)** | **PHA concentration (g L^−1^)** |
| --- | --- | --- | --- |
| 15 | 2.01 ± 0.32 | 13.21 ± 1.94 | 0.26 ± 0.003 |
| 20 | 3.69 ± 0.20 | 49.88 ± 3.05 | 1.85 ± 0.21 |
| 25 | 4.68 ± 0.19 | 70.65 ± 5.07 | 3.31 ± 0.37 |
| **30** | **5.06 ± 0.23** | **79.51 ± 3.19** | **4.03 ± 0.34** |
| 35 | 4.32 ± 0.27 | 60.52 ± 4.69 | 2.62 ± 0.37 |
| 40 | 2.54 ± 0.34 | 30.47 ± 1.25 | 0.78 ± 0.13 |
| 45 | 0.88 ± 0.24 | 20.24 ± 4.48 | 0.18 ± 0.09 |

**Supplementary Table S11.** The effects of nitrogen sources on PHB production (Fermentation conditions: sucrose: 20 g L^−1^, C/N ratio: 40:1, temperature: 30 ℃, fermentation time: 3 d, inoculation concentration: 4 %, pH 8, NaCl: 5 g L^−1^).

| **Nitrogen sources** | **CDW (g L^−1^)** | **PHA content (%)** | **PHA concentration (g L^−1^)** |
| --- | --- | --- | --- |
| **NMP** | **4.96 ± 0.31** | **80.81 ± 3.82** | **3.99 ± 0.06** |
| (NH_4_)_2_SO_4_ | 5.05 ± 0.11 | 78.16 ± 2.18 | 3.95 ± 0.19 |
| (NH_4_)Cl | 5.48 ± 0.32 | 71.80 ± 3.75 | 3.93 ± 0.02 |
| NaNO_3_ | 4.98 ± 0.06 | 45.85 ± 5.31 | 2.28 ± 0.24 |
| NaNO_2_ | 5.38 ± 0.11 | 57.10 ± 5.13 | 3.08 ± 0.34 |
| CH_4_N_2_O | 5.54 ± 0.04 | 76.56 ± 2.88 | 4.24 ± 0.19 |

**Supplementary Table S12.** The comparison of PHA production by *Paracoccus* sp. ZQW-1 with other documented *Paracoccus* sp. strains.

| ***Paracoccus* sp. strain** | **CDW (g L^-1^)** | **PHA content (%)** | **PHA titer (g L^-1^)** | **Reference** |
| --- | --- | --- | --- | --- |
| *Paracoccus* sp. ZQW-1 | 5.21 | 80.5 | 4.19 | This study |
| *Paracoccus* sp. TOH | 3.08 | 81.1 | 2.49 | ^[^[^10^](#_ENREF_10)^]^ |
| *Paracoccus* sp. PXZ | 4.49 | 61.0 | 2.74 | ^[^[^11^](#_ENREF_11)^]^ |
| *Paracoccus* sp. DSM 2944 | 2.48 | 27.4 | 0.68 | ^[^[^12^](#_ENREF_12)^]^ |
| *Paracoccus* sp. DSMZ 17862 | 2.5 | 17.0 | 0.43 | ^[^[^13^](#_ENREF_13)^]^ |
| *Paracoccus* sp. PD1222 | 10.0 | 16.7 | 1.67 | ^[^[^14^](#_ENREF_14)^]^ |
| *Paracoccus* sp. LL1 | 8.73 | 43.2 | 3.77 | ^[^[^15^](#_ENREF_15)^]^ |
| *Paracoccus* sp. ATCC 17741 | 6.5 | 24.0 | 1.56 | ^[^[^15^](#_ENREF_15)^]^ |
| *Paracoccus* sp. DSMZ 413 | 3.0 | 4.0 | 0.12 | ^[^[^16^](#_ENREF_16)^]^ |

**Supplementary Table S13.** The genes in *Paracoccus* sp. ZQW-1 related to PHB metabolism.

| **Sequence name** | **Sequence description** | **Sequence length** | **COG ID** | **COG category** |
| --- | --- | --- | --- | --- |
| Gene2445 | Acetyl-CoA acetyltransferase (*phaA*) | 1176 bp | COG0183 | metabolism |
| Gene2446 | Acetoacetyl-CoA reductase (*phaB*) | 726 bp | COG1028 | metabolism |
| pA_gene0459 | Poly(3-hydroxyalkanoate) polymerase (*phaC1*) | 1803 bp | COG3243 | metabolism |
| Gene0974 | Poly(3-hydroxyalkanoate) polymerase (*phaC2*) | 1875 bp | COG3243 | metabolism |
| Gene1838 | Polyhydroxyalkanoate depolymerase (*phaZ*) | 1344 bp | COG4553 | metabolism |
| Gene1841 | Polyhydroxyalkanoate synthesis repressor (*phaR*) | 588 bp | COG5394 | metabolism |
| Gene2423 | Acyl-CoA synthetase (*fadD*) | 1887 bp | COG0318 | metabolism |
| Gene1236 | Acyl-CoA dehydrogenase (*fadE*) | 1692 bp | COG1960 | metabolism |
| pA_gene0528 | 3-oxoadipyl-CoA thiolase (*fadA*) | 1206 bp | COG0183 | metabolism |
| Gene2763 | Enoyl-CoA hydratase (*fadJ*) | 2184 bp | COG1250 | metabolism |
| Gene2130 | 3-ketoacyl-ACP reductase (*fabG*) | 738 bp | COG1028 | metabolism |
| Gene3998 | glycerol kinase (*glpK*) | 1488 bp | COG0554 | metabolism |
| Gene0634 | glycerol kinase (*glpK*) | 1485 bp | COG0554 | metabolism |
| Gene2925 | glycerol-3-phosphate dehydrogenase (*glpA*) | 1620 bp | COG0578 | metabolism |
| Gene4005 | glycerol-3-phosphate dehydrogenase (*glpA*) | 1593 bp | COG0578 | metabolism |
| Gene2417 | glycerol-3-phosphate dehydrogenase (*gpsA*) | 945 bp | COG0240 | metabolism |

**Supplementary Table S14.** The mechanical characterizations of PHB produced by *Paracoccus* sp. ZQW-1.

| **PHB producing strain** | **Tensile Strength (MPa)** | **Elongation at break (%)** | **Young’s modulus (MPa)** |
| --- | --- | --- | --- |
| *Paracoccus* sp. ZQW-1 | 11.6 | 12.8 | 73.1 |

**Supplementary Table S15.** The molecular weight of PHB produced by *Paracoccus* sp. ZQW-1 and other documented strains.

| **PHB producing strains** | ***M*_w_**  **(g mol^-1^)** | ***M*_n_**  **(g mol^-1^)** | **PDI (*M*_w_/*M*_n_)** | **Reference** |
| --- | --- | --- | --- | --- |
| PHB authentic film (sigma) | 429000 | 319000 | 1.34 | ^[^[^17^](#_ENREF_17)^]^ |
| *Paracoccus* sp. ZQW-1 | 295749 | 92879 | 3.18 | This work |
| *Paracoccus denitrificans* DSMZ 413 | 750000 | 288000 | 2.6 | ^[^[^18^](#_ENREF_18)^]^ |
| *Cupriavidus* sp. CY1 | 269000 | 128700 | 2.09 | ^[^[^19^](#_ENREF_19)^]^ |
| *Cupriavidus necator* (JCM 11282) | 382000 | 92000 | 4.15 | ^[^[^20^](#_ENREF_20)^]^ |
| *Cupriavidus necator* JMP 134 | 620000 | 168000 | 3.7 | ^[^[^18^](#_ENREF_18)^]^ |
| *Cupriavidus necator* NCIMB 11599 | 599000 | 124000 | 2.07 | ^[^[^21^](#_ENREF_21)^]^ |
| *Halomonas salina* YH100 | 195000 | 159000 | 1.22 | ^[^[^22^](#_ENREF_22)^]^ |
| *Halomonas* sp. YLGW01 | 580000 | 430000 | 1.32 | ^[^[^23^](#_ENREF_23)^]^ |
| *Halomonas* sp. JJY01 | 1.33534E6 | 1.12142E6 | 1.2 | ^[^[^24^](#_ENREF_24)^]^ |
| *Bacillus subtilis* MSBN17 | 640000 | 380000 | 1.68 | ^[^[^25^](#_ENREF_25)^]^ |
| *Bacillus* sp. YHY22 | 1.341E6 | 1.129E6 | 1.18 | ^[^[^26^](#_ENREF_26)^]^ |
| *Bacillus* sp. CYR1 | 709000 | 315000 | 2.25 | ^[^[^27^](#_ENREF_27)^]^ |
| *Bacillus* sp. KE4 | 155436 | 143528 | 1.08 | ^[^[^28^](#_ENREF_28)^]^ |
| *Escherichia coli* | 730000 | 1000000 | 1.37 | ^[^[^29^](#_ENREF_29)^]^ |
| *E. coli* JM109 | 285000 | 106500 | 2.64 | ^[^[^30^](#_ENREF_30)^]^ |
| *E. coli* JM109 pUC19-23119phaCABA-04 | 190000 | 100000 | 1.9 | ^[^[^18^](#_ENREF_18)^]^ |
| *Aquabacterium* sp. A7-Y | 229499 | 982610 | 4.28 | ^[^[^31^](#_ENREF_31)^]^ |
| *Azotobacter* *chroococcum* 6B | 710000 | 500000 | 1.42 | ^[^[^32^](#_ENREF_32)^]^ |
| *Alcaligenes latus* DSM 1124 | 131000 | 46000 | 2.86 | ^[^[^33^](#_ENREF_33)^]^ |
| *Burkholderia cepacia* WN-H41 | 62000 | 26000 | 2.4 | ^[^[^34^](#_ENREF_34)^]^ |
| *Caldimonas manganoxidans* JCM 10698T | 753000 | 467700 | 1.61 | ^[^[^35^](#_ENREF_35)^]^ |
| *Priestia* sp. YH4 | 285000 | 105000 | 2.7 | ^[^[^17^](#_ENREF_17)^]^ |
| *Paecilomyces* sp. strain DS1407 | 415000 | 322000 | 1.29 | ^[^[^36^](#_ENREF_36)^]^ |
| *Pseudomonas oleovorans* NRRL B-14682 | 630000 | 328000 | 1.92 | ^[^[^37^](#_ENREF_37)^]^ |
| *Rhodococcus equi* | 642000 | 373000 | 1.72 | ^[^[^38^](#_ENREF_38)^]^ |
| *Synechocystis* sp. PCC 6714 | 1.0519E6 | 316060 | 3.33 | ^[^[^39^](#_ENREF_39)^]^ |

**Supplementary Table S16.** The environmental impacts resulting from the enrichment and culture of *Paracoccus* sp. ZQW-1 (1 cm^3^).

| **Impact category** | **Unit** | **Total** |
| --- | --- | --- |
| Global warming | kg CO_2_ eq | 0.029892 |
| Stratospheric ozone depletion | kg CFC11 eq | 5.57E-09 |
| Ionizing radiation | kBq Co-60 eq | 0.000907 |
| Ozone formation, Human health | kg NO_x_ eq | 6.89E-05 |
| Fine particulate matter formation | kg PM2.5 eq | 3.77E-05 |
| Ozone formation, Terrestrial ecosystems | kg NO_x_ eq | 7.31E-05 |
| Terrestrial acidification | kg SO_2_ eq | 8.83E-05 |
| Freshwater eutrophication | kg P eq | 6.29E-06 |
| Marine eutrophication | kg N eq | 4.95E-07 |
| Terrestrial ecotoxicity | kg 1,4-DCB | 0.053049 |
| Freshwater ecotoxicity | kg 1,4-DCB | 0.000596 |
| Marine ecotoxicity | kg 1,4-DCB | 0.000794 |
| Human carcinogenic toxicity | kg 1,4-DCB | 0.001054 |
| Human non-carcinogenic toxicity | kg 1,4-DCB | 0.014929 |
| Land use | m^2^a crop eq | 0.000387 |
| Mineral resource scarcity | kg Cu eq | 4.34E-05 |
| Fossil resource scarcity | kg oil eq | 0.014854 |
| Water consumption | m^3^ | 0.000348 |

**Supplementary Table S17.** The environmental impacts resulting from the production of 1 cm^3^ PHB fermentation liquid using (NH_4_)_2_SO_4_ as the nitrogen source.

| **Impact category** | **Unit** | **Total** |
| --- | --- | --- |
| Global warming | kg CO_2_ eq | 0.022909 |
| Stratospheric ozone depletion | kg CFC11 eq | 4.98E-09 |
| Ionizing radiation | kBq Co-60 eq | 0.000889 |
| Ozone formation, Human health | kg NO_x_ eq | 6.20E-05 |
| Fine particulate matter formation | kg PM2.5 eq | 3.40E-05 |
| Ozone formation, Terrestrial ecosystems | kg NO_x_ eq | 6.24E-05 |
| Terrestrial acidification | kg SO_2_ eq | 7.64E-05 |
| Freshwater eutrophication | kg P eq | 4.10E-06 |
| Marine eutrophication | kg N eq | 3.18E-07 |
| Terrestrial ecotoxicity | kg 1,4-DCB | 0.014816 |
| Freshwater ecotoxicity | kg 1,4-DCB | 0.000226 |
| Marine ecotoxicity | kg 1,4-DCB | 0.000312 |
| Human carcinogenic toxicity | kg 1,4-DCB | 0.000693 |
| Human non-carcinogenic toxicity | kg 1,4-DCB | 0.009761 |
| Land use | m^2^a crop eq | 0.000294 |
| Mineral resource scarcity | kg Cu eq | 1.25E-05 |
| Fossil resource scarcity | kg oil eq | 0.004891 |
| Water consumption | m^3^ | 7.50E-05 |

**Supplementary Table S18.** The environmental impacts resulting from the production of 1 cm^3^ PHB fermentation liquid by using NMP as specific nitrogen source.

| **Impact category** | **Unit** | **Total** |
| --- | --- | --- |
| Global warming | kg CO_2_ eq | 0.022454 |
| Stratospheric ozone depletion | kg CFC11 eq | 4.88E-09 |
| Ionizing radiation | kBq Co-60 eq | 0.000871 |
| Ozone formation, Human health | kg NO_x_ eq | 6.08E-05 |
| Fine particulate matter formation | kg PM2.5 eq | 3.33E-05 |
| Ozone formation, Terrestrial ecosystems | kg NO_x_ eq | 6.12E-05 |
| Terrestrial acidification | kg SO_2_ eq | 7.49E-05 |
| Freshwater eutrophication | kg P eq | 3.97E-06 |
| Marine eutrophication | kg N eq | 3.04E-07 |
| Terrestrial ecotoxicity | kg 1,4-DCB | 0.014486 |
| Freshwater ecotoxicity | kg 1,4-DCB | 0.000221 |
| Marine ecotoxicity | kg 1,4-DCB | 0.000305 |
| Human carcinogenic toxicity | kg 1,4-DCB | 0.000678 |
| Human non-carcinogenic toxicity | kg 1,4-DCB | 0.009554 |
| Land use | m^2^a crop eq | 0.000289 |
| Mineral resource scarcity | kg Cu eq | 1.22E-05 |
| Fossil resource scarcity | kg oil eq | 0.004800 |
| Water consumption | m^3^ | 7.51E-05 |

**Supplementary Table S19.** The environmental impacts resulting from the enrichment of 1 g dried cells using (NH_4_)_2_SO_4_ as the nitrogen source.

| **Impact category** | **Unit** | **Total** |
| --- | --- | --- |
| Global warming | kg CO_2_ eq | 5.076741 |
| Stratospheric ozone depletion | kg CFC11 eq | 1.10E-06 |
| Ionizing radiation | kBq Co-60 eq | 0.197158 |
| Ozone formation, Human health | kg NO_x_ eq | 0.013749 |
| Fine particulate matter formation | kg PM2.5 eq | 0.007546 |
| Ozone formation, Terrestrial ecosystems | kg NO_x_ eq | 0.013840 |
| Terrestrial acidification | kg SO_2_ eq | 0.016939 |
| Freshwater eutrophication | kg P eq | 0.000907 |
| Marine eutrophication | kg N eq | 7.00E-05 |
| Terrestrial ecotoxicity | kg 1,4-DCB | 3.227788 |
| Freshwater ecotoxicity | kg 1,4-DCB | 0.049514 |
| Marine ecotoxicity | kg 1,4-DCB | 0.068379 |
| Human carcinogenic toxicity | kg 1,4-DCB | 0.153270 |
| Human non-carcinogenic toxicity | kg 1,4-DCB | 2.157115 |
| Land use | m^2^a crop eq | 0.064418 |
| Mineral resource scarcity | kg Cu eq | 0.002666 |
| Fossil resource scarcity | kg oil eq | 1.075205 |
| Water consumption | m^3^ | 0.015844 |

**Supplementary Table S20.** The environmental impacts resulting from the enrichment of 1 g dried cells using NMP as specific nitrogen source.

| **Impact category** | **Unit** | **Total** |
| --- | --- | --- |
| Global warming | kg CO_2_ eq | 5.108017 |
| Stratospheric ozone depletion | kg CFC11 eq | 1.11E-06 |
| Ionizing radiation | kBq Co-60 eq | 0.198284 |
| Ozone formation, Human health | kg NO_x_ eq | 0.013833 |
| Fine particulate matter formation | kg PM2.5 eq | 0.007591 |
| Ozone formation, Terrestrial ecosystems | kg NO_x_ eq | 0.013925 |
| Terrestrial acidification | kg SO_2_ eq | 0.017041 |
| Freshwater eutrophication | kg P eq | 0.000903 |
| Marine eutrophication | kg N eq | 6.87E-05 |
| Terrestrial ecotoxicity | kg 1,4-DCB | 3.239493 |
| Freshwater ecotoxicity | kg 1,4-DCB | 0.049755 |
| Marine ecotoxicity | kg 1,4-DCB | 0.068707 |
| Human carcinogenic toxicity | kg 1,4-DCB | 0.154045 |
| Human non-carcinogenic toxicity | kg 1,4-DCB | 2.167527 |
| Land use | m^2^a crop eq | 0.064915 |
| Mineral resource scarcity | kg Cu eq | 0.002681 |
| Fossil resource scarcity | kg oil eq | 1.083049 |
| Water consumption | m^3^ | 0.016246 |

**Supplementary Table S21.** The environmental impacts of PHB production using (NH_4_)_2_SO_4_ as the nitrogen source.

| **Impact category** | **Unit** | **Total** |
| --- | --- | --- |
| Global warming | kg CO_2_ eq | 9.046071 |
| Stratospheric ozone depletion | kg CFC11 eq | 8.77837E-05 |
| Ionizing radiation | kBq Co-60 eq | 0.272665 |
| Ozone formation, Human health | kg NO_x_ eq | 0.018614 |
| Fine particulate matter formation | kg PM2.5 eq | 0.010294 |
| Ozone formation, Terrestrial ecosystems | kg NO_x_ eq | 0.018754 |
| Terrestrial acidification | kg SO_2_ eq | 0.022955 |
| Freshwater eutrophication | kg P eq | 0.001266 |
| Marine eutrophication | kg N eq | 9.76168E-05 |
| Terrestrial ecotoxicity | kg 1,4-DCB | 5.040738 |
| Freshwater ecotoxicity | kg 1,4-DCB | 0.074500 |
| Marine ecotoxicity | kg 1,4-DCB | 0.106624 |
| Human carcinogenic toxicity | kg 1,4-DCB | 0.361187 |
| Human non-carcinogenic toxicity | kg 1,4-DCB | 5.442716 |
| Land use | m^2^a crop eq | 0.088743 |
| Mineral resource scarcity | kg Cu eq | 0.004169 |
| Fossil resource scarcity | kg oil eq | 1.485701 |
| Water consumption | m^3^ | 0.023316 |

**Supplementary Table S22.** The environmental impacts of PHB production using NMP as specific nitrogen source.

| **Impact category** | **Unit** | **Total** |
| --- | --- | --- |
| Global warming | kg CO_2_ eq | 8.247300 |
| Stratospheric ozone depletion | kg CFC11 eq | 7.96463E-05 |
| Ionizing radiation | kBq Co-60 eq | 0.248840 |
| Ozone formation, Human health | kg NO_x_ eq | 0.016996 |
| Fine particulate matter formation | kg PM2.5 eq | 0.009398 |
| Ozone formation, Terrestrial ecosystems | kg NO_x_ eq | 0.017124 |
| Terrestrial acidification | kg SO_2_ eq | 0.020958 |
| Freshwater eutrophication | kg P eq | 0.001143 |
| Marine eutrophication | kg N eq | 8.71114E-05 |
| Terrestrial ecotoxicity | kg 1,4-DCB | 4.588920 |
| Freshwater ecotoxicity | kg 1,4-DCB | 0.067905 |
| Marine ecotoxicity | kg 1,4-DCB | 0.097164 |
| Human carcinogenic toxicity | kg 1,4-DCB | 0.328699 |
| Human non-carcinogenic toxicity | kg 1,4-DCB | 4.951543 |
| Land use | m^2^a crop eq | 0.081135 |
| Mineral resource scarcity | kg Cu eq | 0.003802 |
| Fossil resource scarcity | kg oil eq | 1.357808 |
| Water consumption | m^3^ | 0.021632 |

**Supplementary Table S23.** The price of chemicals used for techno-economic assessment.

| **Inputs** | **Price ($ ton^−1^/$ kWh^−1^)** | **Reference** |
| --- | --- | --- |
| Tryptone | 12000 | a |
| Yeast Extract | 1500 | ^[^[^40^](#_ENREF_40)^]^ |
| NaCl | 100 | b |
| Water | 0.22 | ^[^[^41^](#_ENREF_41)^]^ |
| Sucrose | 400 | ^[^[^40^](#_ENREF_40)^]^ |
| (NH_4_)_2_SO_4_ | 380 | ^[^[^42^](#_ENREF_42)^]^ |
| Na_2_HPO_4_•12H_2_O | 520 | ^[^[^43^](#_ENREF_43)^]^ |
| KH_2_PO_4_ | 1400 | ^[^[^43^](#_ENREF_43)^]^ |
| MgSO_4_•7H_2_O | 1220 | ^[^[^43^](#_ENREF_43)^]^ |
| H_3_BO_3_ | 800 | ^[^[^43^](#_ENREF_43)^]^ |
| CoCl_2_•6H_2_O | 4060 | ^[^[^43^](#_ENREF_43)^]^ |
| ZnSO_4_•7H_2_O | 1180 | ^[^[^43^](#_ENREF_43)^]^ |
| MnSO_4_•5H_2_O | 1500 | c |
| (NH_4_)_6_Mo_7_O_24_•4H_2_O | 5000 | d |
| CuSO_4_•5H_2_O | 840 | ^[^[^43^](#_ENREF_43)^]^ |
| NiCl_2_•6H_2_O | 2440 | ^[^[^43^](#_ENREF_43)^]^ |
| NaClO | 390 | ^[^[^44^](#_ENREF_44)^,^ [^45^](#_ENREF_45)^]^ |
| Chloroform | 500 | ^[^[^46^](#_ENREF_46)^]^ |
| Electricity | 0.1 | ^[^[^47^](#_ENREF_47)^]^ |
| PHB | 5000 | ^[^[^43^](#_ENREF_43)^,^ [^48-50^](#_ENREF_48)^]^ |

**Taken from the online trade:**

**a** https://shineherb.en.made-in-china.com/product/VQeUqzICHMpR/China-Wholesale-Price-Casein-Peptone-Tryptone-Powder-CAS-9064-67-9.html?pv_id=1imm68smse7e&faw_id=1imm69ai2dc7

**b** https://yuyuangroup.en.made-in-china.com/product/sSrJMnZonXpL/China-Sodium-Chloride-Industrial-Salt-Nacl.html?pv_id=1il0l3f4ibae&faw_id=1il0l62f6aa5.

**c** https://cnlygzha.en.made-in-china.com/product/RwQflcDdhikY/China-Manganese-Sulfate-China-Factory-Price-FCC-7785-87-7-Food-Grade-Monohydrate-Manganese-Sulfate-Mnso4.html?pv_id=1il0h2ss958b&faw_id=1il0h3mrn875.

**d** https://chuanghaibiotech.en.made-in-china.com/product/QEyRuYVosMpl/China-High-Quality-CAS-12054-85-2-Ammonium-Molybdate-Tetrahydrate.html?pv_id=1imomqkc2459&faw_id=1imomqr4d0f0.

The techno-economic assessment was applied to evaluate the economic potential of wastewater upgrading platform. To simplify the calculation, equipment depreciation was temporarily ignored, focusing on operating expense (OPEX). Several assumptions were listed as follows:

(1) 1000 tons of NMP wastewater.

(2) The concentration of NMP wastewater was 1.4 g L^−1^.

(3) The electricity cost was 0.1$ kWh^−1^.

(4) Approximately 1 ton of sludge was produced from wastewater treatment.

(5) The energy consumption for wastewater treatment/PHB fermentation was assumed to be 0.5 kWh/m^3^.^[^[^51^](#_ENREF_51)^]^

***Case A: Bioremediation of 1000 tons of NMP wastewater.***

The wastewater was treated in batches, each processing 100 tons. After each batch treatment, the wastewater was discharged, while the microbial biomass was retained for subsequent batches. This approach avoided repeated microbial cultivation, aligning with real industrial operation.

The cost of medium = (5×1500+10×12000+5×100) ×10^−6^+0.22×10^−3^ = 0.12822 $ L^−1^

The total cost of medium = 0.12822 $ L^−1^×4000 L = 512.88 $

The energy consumption for *Paracoccus* sp. ZQW-1 cultivation consisted of stirring (power = 1 kW), temperature control (power = 0.5 kW), and aeration (power = 0.5 kW).

The total energy consumption for strain cultivation = 1 kW×24 h+0.5 kW×24 h+0.5 kW×24 h = 48 kWh

The cost of strain cultivation = 48 kWh×0.1$ kWh^−1^= 4.8 $

The cost of wastewater treatment = 0.5 kWh/m^3^×100 m^3^ d^−1^×10 d×0.1$ kWh^−1^ = 50 $

The cost of sludge disposal was estimated to be 70 $ ton^−1^.

The total cost of sludge disposal=70 $ ton^−1^×1 ton = 70 $

The labor cost=4×20 $×10 d=800 $

The total operating expense (OPEX)=512.88+4.8+50+70+800 = 1437.68 $

The cost of NMP wastewater treatment = 1.44 $ ton^−1^

***Case B: Upgrading NMP wastewater into PHB.***

The wastewater upgrading was conducted in batches, each processing 100 tons.

The cost of medium = (5×1500+10×12000+5×100) ×10^−6^+0.22×10^−3^ = 0.12822 $ L^−1^

The total cost of medium (each batch) = 0.12822 $ L^−1^×4000 L = 512.88 $

The cost of strain cultivation = 48 kWh×0.1$ kWh^−1^ = 4.8 $

The cost of substrates **=** (400×20+9×520+1.5×1400+0.2×1220+0.3×800+0.2×4060+0.1×1180+0.1×1500+0.03×5000+0.02×2440+0.01×840) ×10^−6^ = 0.0165512 $ L^−1^

The cost of substrates (each batch) **=** 0.0165512 $ L^−1^×100000 L = 1655.12 $

Considering that PHB production and NMP wastewater treatment were carried out simultaneously, we assumed that the operating conditions of PHB production and wastewater treatment were consistent based on the experimental data.

The cost of energy consumption for PHB production (each batch) = 0.5 kWh/m^3^×100 m^3^×0.1$ kWh^−1^ ×3 d = 15 $

The total labor cost=5×20 $×3 d = 300 $

The cost of PHB separation was estimated to be 50% of substrate costs.

The cost of PHB separation = 1655.12 $×50% = 827.56 $

The amount of bacterial residues = 5.21 g L^−1^×100000 L×20%×10^−6^ = 0.1 ton

The cost of residues disposal was estimated to be 70 $ ton^−1^.

The cost of residues disposal = 7 $

The income of recovered PHB (each batch) = 4.19 g L^−1^×5000 $ ton^−1^×10^−6^×100000 L = 2095 $

The total cost of upgrading NMP wastewater (1000 ton) into PHB = 10× (512.88+4.8+1655.12+15+827.56+7-2095) +300 = 9573.6 $

The cost of NMP wastewater treatment = 9.57 $ ton^−1^

The calculated breakeven point of PHB = 7.28 $ kg^−1^

**Sensitivity analysis:**

(1) Cell dry weight (±20%)

With a 20% increase in cell dry weight, the breakeven point for PHB can drop to 6.0 $ kg^−1^.

(2) PHB content (±10%)

With a 10% increase in PHB content, the breakeven point for PHB can drop to 6.44 $ kg^−1^.

(3) Carbon source dosage (±20%)

With a 20% increase in carbon source dosage, the breakeven point for PHB can drop to 6.71 $ kg^−1^.

***Case C: Upgrading NMP wastewater by engineered Paracoccus sp. ZQW-1.***

The inherent tunability of microorganisms offered a promising avenue for optimizing carbon source conversion efficiency. The current conversion rate was 0.21g PHB/g sucrose, which indicated the significant room for improvement. Herein, we hypothesized that the advanced genetic engineering techniques could enhance carbon source conversion rate to 40% and 50.3% (theoretical conversion rate), respectively. Based on the optimized operating parameters, we recalculated the economic benefits of wastewater treatment.

(i) 40% of conversion rate

The income of recovered PHB (each batch) = 8 g L^−1^×5000 $ ton^−1^×10^−6^×100000 L = 4000 $

The total profit of upgrading NMP wastewater = 10× (4000-512.88-4.8-1655.12-15-827.56-7)-300 = 9476.4 $

The profit of upgrading NMP wastewater = 9.48 $ ton^−1^

(ii) 50.3% of conversion rate (theoretical conversion rate)

The income of recovered PHB (each batch) = 10.06 g L^−1^×5000 $ ton^−1^×10^−6^×100000 L = 5030 $

The total profit of upgrading NMP wastewater = 10× (5030-512.88-4.8-1655.12-15-827.56-7)-300 = 19776.4 $

The profit of upgrading NMP wastewater = 19.78 $ ton^−1^

Using one New Materials Technology Co., Ltd. in ZhenJiang as a case study, it produced 63.36 tons of highly concentrated NMP wastewater per year (30 g L^−1^). The high concentration NMP wastewater was diluted into 1357.71 tons of low concentration NMP wastewater (1.4 g L^−1^).

The profit of upgrading wastewater into PHB (40% of conversion rate) = 1357.71 tons×9.48 $ ton^−1^-1294.35 tons×0.22 $ ton^−1^ = 12586.33 $

The profit of upgrading wastewater into PHB (50% of conversion rate) = 1357.71 tons×19.78 $ ton^−1^-1294.35 tons×0.22 $ ton^−1^ = 26570.74 $

**Supplementary Table S24.** Representative and marketable high-value commodities recovered from WWTPs.

| **Commodities** | **Highlights** | **Challenges and opportunities** |
| --- | --- | --- |
| 1. Biogas | ● Recovered by anaerobic digestion  ● Well-established technology and wide implementation in WWTPs | ● Low financial return ($1.4−9.5 GJ^−1^) ^[^[^52^](#_ENREF_52)^]^  ● Restricted applicability scope |
| 1. Phosphorus | ● Commonly recovered by chemical precipitation ^[^[^53^](#_ENREF_53)^]^  ● Wastewater P recovery could fulfill 15-20% of global P demand ^[^[^54^](#_ENREF_54)^]^ | ● Low recovery efficiency ^[^[^55^](#_ENREF_55)^]^  ● Uncontrollable product quality  ● Limited market price ($200−1200 ton^−1^) ^[^[^56^](#_ENREF_56)^]^ |
| 1. Biosolid | ● Applied as organic fertilizers and soil conditioners  ● Reducing fertilizer price pressures on agriculture  ● Decreasing greenhouse gas emissions associated with chemical fertilizer production | ● Environmental risks tied to pathogens, heavy metals, and emerging contaminants (e.g., micro-plastics) in biosolid  ● Market increase is expected from $8.7 billion in 2023 to $12 billion by 2030 ^[^[^57^](#_ENREF_57)^]^ |
| 1. Cellulose | ● Commonly recovered from used toilet paper by physical separation ^[^[^58^](#_ENREF_58)^]^  ● Utilized as feedstock for biofuel and paper production | ● Operational challenges in source separation of toilet paper  ● Market increase is expected from $17.6 billion in 2022 to $42.24 billion by 2032 ^[^[^59^](#_ENREF_59)^]^ |
| 1. Protein | ● Lower environmental footprint than conventional protein sources  ● Utilized to strengthen food products with amino acids, vitamins, and minerals | ● Lack of efficient selective separation technologies  ● Market increase is expected from $13.1 billion in 2022 to $75.9 billion by 2032 ^[^[^59^](#_ENREF_59)^]^ |
| 1. PHA | ● Upcycling wastewater into PHA offers a promising solution to reduce environmental impact  ● Serving as eco-friendly alternatives to conventional plastics  ● Serving as value-added products for drug delivery, scaffold materials, and tissue engineering | ● Higher production costs than conventional petroleum-based plastics  ● Sustainability-driven market transformation  ● Market increase is expected from $93 million in 2022 to $195 million by 2028 ^[^[^59^](#_ENREF_59)^]^ |

**Supplementary Table S25.** The strains and plasmids used for *nmpB* gene knockout.

| **Strain or plasmid** | **Description** | **Source** |
| --- | --- | --- |
| **Strains** |  |  |
| *Paracoccus* sp. ZQW-1 | Wild-type strain; able to consume NMP | This study |
| *Paracoccus* sp. ZQW-1-Δ*nmpB* | ZQW-1 mutant; unable to consume NMP | This study |
| **Plasmids** |  |  |
| pRK600 | Conjugation helper plasmid | Lab stock |
| pJQ200SK | Gm^r^ Mob^+^ *orip15A lacZa*^+^ SacB, suicide plasmid | Lab stock |
| *nmpB*-pJQ200SK | Gm^r^, *nmpB* gene deletion plasmid | This study |

**Supplementary Table S26.** The primers used for *nmpB* gene knockout.

| **Primer** | **Sequence (5'→3')** |
| --- | --- |
| *nmpB*-pJQ200SK-F | GGAAACAGCTATGACCATGATTACGAATTCCAGATCGCCGAGGTGATC |
| *nmpB*-pJQ200SK-R | TGCAGGTCGACTCTAGAGGATCCGGAACTGATCGGCTATGCG |

**Supplementary Table S27.** The primers used in RT-qPCR experiments.

| **Primer** | **Sequence 5′-3′** | **Tm** | **GC%** | **Source** |  |
| --- | --- | --- | --- | --- | --- |
| 16S rRNA_F | GTGAGTGGAATTCCGAGTGTA | 55 | 48 | This study | |
| 16S rRNA_R | CGTTAGGTGTGTCACCGAACA | 58 | 52 | This study |  |
| *phaC1*_F | TTCACCAATCCCGAGGTTATC | 55 | 48 | This study |  |
| *phaC1*_R | GATCCAGGCCGGCACGATTAG | 61 | 62 | This study |  |
| *phaC2*_F | TCGACCTCTCGCCGCAAAACT | 63 | 57 | This study |  |
| *phaC2*_R | GCAATAACCGACGGCATGAAC | 59 | 52 | This study |  |
| *phaA*_F | TGCTCGACACCATGATCAAGG | 59 | 52 | This study |  |
| *phaA*_R | CTTGATGGTCACGGGCACGAT | 62 | 57 | This study |  |
| *phaB*_F | CGCATCGTCAACATCAGCTCG | 60 | 57 | This study |  |
| *phaB*_R | TTCAGCACCTTCTCGTCGATG | 58 | 52 | This study |  |

**Reference**

[1] J. Quandt, M.F. Hynes, Versatile suicide vectors which allow direct selection for gene replacement in Gram-negative bacteria, Gene, 1993, 127, 15-21.

[2] S. Xu, X. Wang, F. Zhang, Y. Jiang, Y. Zhang, M. Cheng, X. Yan, Q. Hong, J. He, J. Qiu, PicR as a MarR Family Transcriptional Repressor Multiply Controls the Transcription of Picolinic Acid Degradation Gene Cluster *pic* in *Alcaligenes faecalis* JQ135, Appl. Environ. Microbiol. 2022, 88, e00172-00122.

[3] Y. Jiang, K. Wang, L. Xu, L. Xu, Q. Xu, Y. Mu, Q. Hong, J. He, J. Jiang, J. Qiu, DipR, a GntR/FadR-family transcriptional repressor: regulatory mechanism and widespread distribution of the *dip* cluster for dipicolinic acid catabolism in bacteria, Nucleic Acids Res. 2024, 52, 10951-10964.

[4] S. Cai, T. Cai, S. Liu, Q. Yang, J. He, L. Chen, J. Hu, Biodegradation of *N*-Methylpyrrolidone by *Paracoccus* sp. NMD-4 and its degradation pathway, Int. Biodeter. Biodegrad. 2014, 93, 70-77.

[5] J. Wang, X. Liu, X. Jiang, L. Zhang, C. Hou, G. Su, L. Wang, Y. Mu, J. Shen, Nitrate stimulation of *N*-Methylpyrrolidone biodegradation by *Paracoccus pantotrophus*: Metabolite mechanism and Genomic characterization, Bioresour. Technol. 2019, 294, 122185.

[6] P. Jančová, M. Dvořáčková, J. Houser, L. Husárová, M. Julinová, J. Růžička, K. Křížek, N-methyl-2-pyrrolidone-degrading bacteria from activated sludge, Water Sci. Technol. 2015, 71, 776-782.

[7] X. Dong, J. Hu, Y. Zhang, A *Bacillus* NMP-2 with *N*-methylpyrrolidone degradation ability and its application, CN106591169B in Chinese, 2016.

[8] J. Shen, J. Wang, J. He, Q. Chi, H. Zhang, H. Wang, X. Jiang, *N*-methylpyrrolidone degrading bacteria and its application in wastewater treatment, CN112574916B in Chinese, 2022.

[9] Y. Gao, T. Chen, Y. Hou, R. Xue, R. Liu, F. Chen, Y. Zhang, B.E. Rittmann, The roles of *Methylobacterium organophilum* and *Sphingomonas melonis* for accelerating *N*-methyl pyrrolidone (NMP) biodegradation, J. Water Process Eng. 2023, 56, 104327.

[10] L. Zhao, S. Cai, J. Zhang, Q. Zhang, L. Chen, X. Ji, R. Zhang, T. Cai, Poly(3-hydroxybutyrate) biosynthesis under non-sterile conditions: Piperazine as nitrogen substrate control switch, Int. J. Biol. Macromol. 2022, 209, 1457-1464.

[11] L. Zhao, J. Pan, S. Cai, L. Chen, T. Cai, X.M. Ji, Biosynthesis of poly(3-hydroxybutyrate) by *N,N*-dimethylformamide degrading strain *Paracoccus* sp. PXZ: A strategy for resource utilization of pollutants, Bioresour. Technol. 2023, 384, 129318.

[12] E. Ucisik-Akkaya, O. Ercan, S.K. Yesiladali, T. Öztürk, E. Ubay‐Cokgor, D. Orhon, C. Tamerler, Z.P. Çakar, Enhanced polyhydroxyalkanoate production by *Paracoccus pantotrophus* from glucose and mixed substrate, Fresenius Environ. Bull. 2009, 18, 2013-2022.

[13] K. Szacherska, K. Moraczewski, P. Rytlewski, S. Czaplicki, S. Ciesielski, P. Oleskowicz-Popiel, J. Mozejko-Ciesielska, Polyhydroxyalkanoates production from short and medium chain carboxylic acids by *Paracoccus homiensis*, Sci. Rep. 2022, 12 7263.

[14] A. Olaya-Abril, V.M. Luque-Almagro, I. Manso, A.J. Gates, C. Moreno-Vivian, D.J. Richardson, M.D. Roldan, Poly(3-hydroxybutyrate) hyperproduction by a global nitrogen regulator *NtrB* mutant strain of *Paracoccus denitrificans* PD1222, FEMS Microbiol. Lett. 2018, 365, fnx251.

[15] P. Kumar, H.B. Jun, B.S. Kim, Co-production of polyhydroxyalkanoates and carotenoids through bioconversion of glycerol by *Paracoccus* sp. strain LL1, Int. J. Biol. Macromol. 2018, 107, 2552-2558.

[16] M.J. Mota, R.P. Lopes, M.M.Q. Simoes, I. Delgadillo, J.A. Saraiva, Effect of High Pressure on Paracoccus denitrificans Growth and Polyhydroxyalkanoates Production from Glycerol, Appl. Biochem. Biotechnol. 2019, 188, 810-823.

[17] H.J. Jung, S.H. Kim, N. Shin, S.-J. Oh, J.H. Hwang, H.J. Kim, Y.-H. Kim, S.K. Bhatia, J.-M. Jeon, J.-J. Yoon, Y.-H. Yang, Polyhydroxybutyrate (PHB) production from sugar cane molasses and tap water without sterilization using novel strain, *Priestia* sp. YH4, Int. J. Biol. Macromol. 2023, 250, 126152.

[18] G. Mothes, C. Schnorpfeil, J.U. Ackermann, Production of PHB from Crude Glycerol, Engineering in Life Sciences, 2007, 7, 475-479.

[19] M.V. Reddy, Y. Mawatari, R. Onodera, Y. Nakamura, Y. Yajima, Y.-C. Chang, Bacterial conversion of waste into polyhydroxybutyrate (PHB): A new approach of bio-circular economy for treating waste and energy generation, Bioresour. Technol. Rep. 2019, 7, 100246.

[20] C.U. Ugwu, Y. Tokiwa, H. Aoyagi, Utilization of Broken Rice for the Production of Poly(3-hydroxybutyrate), J. Polym. Environ. 2011, 20, 254-257.

[21] S.M. Lee, H.-J. Lee, S.H. Kim, M.J. Suh, J.Y. Cho, S. Ham, J.-M. Jeon, J.-J. Yoon, S.K. Bhatia, R. Gurav, E.Y. Lee, Y.-H. Yang, Screening of the strictly xylose-utilizing *Bacillus* sp. SM01 for polyhydroxybutyrate and its co-culture with *Cupriavidus necator* NCIMB 11599 for enhanced production of PHB, Int. J. Biol. Macromol. 2021, 181, 410-417.

[22] E. Hernández-Núñez, C.A. Martínez-Gutiérrez, A. López-Cortés, M.L. Aguirre-Macedo, C. Tabasco-Novelo, M.O. González-Díaz, J.Q. García-Maldonado, Physico-chemical Characterization of Poly(3-Hydroxybutyrate) Produced by *Halomonas salina*, Isolated from a Hypersaline Microbial Mat, J. Polym. Environ. 2019, 27, 1105-1111.

[23] B. Kim, S.J. Oh, J.H. Hwang, H.J. Kim, N. Shin, S.K. Bhatia, J.-M. Jeon, J.-J. Yoon, J. Yoo, J. Ahn, J.-H. Park, Y.-H. Yang, Polyhydroxybutyrate production from crude glycerol using a highly robust bacterial strain *Halomonas* sp. YLGW01, Int. J. Biol. Macromol. 2023, 236, 123997.

[24] J.-K. Park, J.-M. Jeon, Y.-H. Yang, S.-H. Kim, J.-J. Yoon, Efficient polyhydroxybutyrate production using acetate by engineered *Halomonas* sp. JJY01 harboring acetyl-CoA acetyltransferase, Int. J. Biol. Macromol. 2024, 254, 127475.

[25] G. Sathiyanarayanan, G. Saibaba, G. Seghal Kiran, J. Selvin, A statistical approach for optimization of polyhydroxybutyrate production by marine *Bacillus subtilis* MSBN17, Int. J. Biol. Macromol. 2013, 59, 170-177.

[26] H.-J. Lee, S.-G. Kim, D.-H. Cho, S.K. Bhatia, R. Gurav, S.-Y. Yang, J. Yang, J.-M. Jeon, J.-J. Yoon, K.-Y. Choi, Y.-H. Yang, Finding of novel lactate utilizing *Bacillus* sp. YHY22 and its evaluation for polyhydroxybutyrate (PHB) production, Int. J. Biol. Macromol. 2022, 201, 653-661.

[27] M. Venkateswar Reddy, Y. Mawatari, Y. Yajima, C. Seki, T. Hoshino, Y.-C. Chang, Poly-3-hydroxybutyrate (PHB) production from alkylphenols, mono and poly-aromatic hydrocarbons using *Bacillus* sp. CYR1: A new strategy for wealth from waste, Bioresour. Technol. 2015, 192, 711-717.

[28] S.A. Acharjee, P. Bharali, D. Ramachandran, V. Kanagasabai, M. Gogoi, S. Hazarika, P.J. Koch, N. Dutta, G.B. Maadurshni, J. Manivannan, S. Kumari, B. Walling, B. Gogoi, Alemtoshi, V. Sorhie, V. Vishawkarma, Polyhydroxybutyrate (PHB)-Based sustainable bioplastic derived from *Bacillus* sp. KE4 isolated from kitchen waste effluent, Sustainable Chem. Pharm. 2024, 39, 101507.

[29] Y.T. Horng, C.C. Chien, Y.H. Wei, S.Y. Chen, J.C.W. Lan, Y.M. Sun, P.C. Soo, Functional cis‐expression of *phaCAB* genes for poly(3‐hydroxybutyrate) production by *Escherichia coli*, Lett. Appl. Microbiol. 2011, 52,475-483.

[30] D.R. T.V.N. Ramachander, A. Belhekar, S.K. Rawal, Synthesis of PHB by recombinant *E. coli* harboring an approximately 5 kb genomic DNA fragment from Streptomyces aureofaciens NRRL 2209, Int. J. Biol. Macromol. 2002, 31, 63-69.

[31] L. Feng, J. Yan, Z. Jiang, X. Chen, Z. Li, J. Liu, X. Qian, Z. Liu, G. Liu, C. Liu, Y. Wang, G. Hu, W. Dong, Z. Cui, Characterization of polyhydroxybutyrate (PHB) synthesized by newly isolated rare actinomycetes *Aquabacterium* sp. A7-Y, Int. J. Biol. Macromol. 2023, 232, 123366.

[32] J.C. Quagliano, F. Amarilla, E.G. Fernandes, D. Mata, S.S. Miyazaki, Effect of simple and complex carbon sources, low temperature culture and complex carbon feeding policies on poly-3-hydroxybutyric acid (PHB) content and molecular weight (Mw) from *Azotobacter chroococcum* 6B, World J. Microb. Biot. 2001, 17, 9-14.

[33] P.A.L. Wong, M.K. Cheung, W.-H. Lo, H. Chua, P.H.F. Yu, Investigation of the effects of the types of food waste utilized as carbon source on the molecular weight distributions and thermal properties of polyhydroxybutyrate produced by two strains of microorganisms, e-Polymers, 2004, 31, 1-11.

[34] B. Zheng, J. Lu, Y. Tong, H. Li, Q. Chen, Isolation and Characterization of Poly(3-hydroxybutyrate)-Producing Bacteria from Aerobic Sludge, Appl. Biochem. Biotechnol. 2014, 175, 421-427.

[35] L.-J. Hsiao, M.-C. Lee, P.-J. Chuang, Y.-Y. Kuo, J.-H. Lin, T.-M. Wu, S.-Y. Li, The production of poly(3-hydroxybutyrate) by thermophilic *Caldimonas manganoxidans* from glycerol, J. Polym.Res. 2018, 25, 85.

[36] F. Li, Z. Guo, N. Wang, H. Xia, D. Liu, S. Chen, Biodegradation of poly(3-hydroxybutyrate)-derived polymers with different 4-hydroxybutyrate fractions by a novel depolymerase from *Paecilomyces* sp. 1407, Polym. Degrad. Stab. 2019, 159, 107-115.

[37] R.D. Ashby, D.K.Y. Solaiman, G.D. Strahan, C. Zhu, R.C. Tappel, C.T. Nomura, Glycerine and levulinic acid: Renewable co-substrates for the fermentative synthesis of short-chain poly(hydroxyalkanoate) biopolymers, Bioresour. Technol. 2012, 118, 272-280.

[38] N. Altaee, A. Fahdil, E. Yousif, K. Sudesh, Recovery and subsequent characterization of polyhydroxybutyrate from *Rhodococcus equi* cells grown on crude palm kernel oil, J. Taibah Univ. Sci. 2018, 10, 543-550.

[39] R.R. de Sousa Junior, C.A.S. dos Santos, N.M. Ito, A.N. Suqueira, M. Lackner, D.J. dos Santos, PHB Processability and Property Improvement with Linear-Chain Polyester Oligomers Used as Plasticizers, Polymers, 2022, 14, 4197.

[40] A.A. Koutinas, B. Yepez, N. Kopsahelis, D.M.G. Freire, A.M. de Castro, S. Papanikolaou, I.K. Kookos, Techno-economic evaluation of a complete bioprocess for 2,3-butanediol production from renewable resources, Bioresour. Technol. 2016, 204, 55-64.

[41] H. Zhou, Y. Ren, Z. Li, M. Xu, Y. Wang, R. Ge, X. Kong, L. Zheng, H. Duan, Electrocatalytic upcycling of polyethylene terephthalate to commodity chemicals and H_2_ fuel, Nat. Commun. 2021, 12, 4679.

[42] R. Wang, K. Yang, C. Wong, H. Aguirre-Villegas, R. Larson, F. Brushett, M. Qin, S. Jin, Electrochemical ammonia recovery and co-production of chemicals from manure wastewater, Nat. Sustain. 2024, 7, 179-190.

[43] C. Amabile, T. Abate, R. Muñoz, S. Chianese, D. Musmarra, Techno-economic assessment of biopolymer production from methane and volatile fatty acids: effect of the reactor size and biomass concentration on the poly(3-hydroxybutyrate-co-3-hydroxyvalerate) selling price, Sci. Total Environ. 2024, 929, 172599.

[44] N. Rajendran, T. Runge, R.D. Bergman, P. Nepal, C. Houtman, Techno-economic analysis and life cycle assessment of cellulose nanocrystals production from wood pulp, Bioresour. Technol. 2023, 377, 128955.

[45] C. Fernández-Dacosta, J.A. Posada, R. Kleerebezem, M.C. Cuellar, A. Ramirez, Microbial community-based polyhydroxyalkanoates (PHAs) production from wastewater: Techno-economic analysis and ex-ante environmental assessment, Bioresour. Technol. 2015, 185, 368-377.

[46] N. Rajendran, J. Han, Techno-economic analysis of food waste valorization for integrated production of polyhydroxyalkanoates and biofuels, Bioresour. Technol. 2022, 348, 126796.

[47] M. Du, R. Xue, W. Yuan, Y. Cheng, Z. Cui, W. Dong, B. Qiu, Tandem Integration of Biological and Electrochemical Catalysis for Efficient Polyester Upcycling under Ambient Conditions, Nano Lett. 2024, 24, 9768-9775.

[48] M.R. Kosseva, E. Rusbandi, Trends in the biomanufacture of polyhydroxyalkanoates with focus on downstream processing, Int. J. Biol. Macromol. 2018, 107, 762-778.

[49] N.A. Manikandan, K. Pakshirajan, G. Pugazhenthi, Techno-economic assessment of a sustainable and cost-effective bioprocess for large scale production of polyhydroxybutyrate, Chemosphere, 2021, 284, 131371.

[50] T. Abate, C. Amabile, R. Muñoz, S. Chianese, D. Musmarra, Polyhydroxyalkanoate recovery overview: properties, characterizations, and extraction strategies, Chemosphere, 2024, 356, 141950.

[51] Y. Gu, Y. Li, X. Li, P. Luo, H. Wang, X. Wang, J. Wu, F. Li, Energy Self-sufficient Wastewater Treatment Plants: Feasibilities and Challenges, Energy Procedia, 2017, 105, 3741-3751.

[52] A.H. Bhatt, L. Tao, Economic Perspectives of Biogas Production via Anaerobic Digestion, in: Bioengineering, 2020, 7.

[53] M.A. Latif, C.M. Mehta, D.J. Batstone, Enhancing soluble phosphate concentration in sludge liquor by pressurised anaerobic digestion, Water Res. 2018, 145, 660-666.

[54] Z. Yuan, S. Pratt, D.J. Batstone, Phosphorus recovery from wastewater through microbial processes, Curr. Opin. Biotechnol. 2012, 23, 878-883.

[55] L. Egle, H. Rechberger, M. Zessner, Overview and description of technologies for recovering phosphorus from municipal wastewater, Resour. Conserv. Recy. 2015, 105, 325-346.

[56] Y. Liu, J. Zhou, Z. Zhang, X. Li, D. Yang, Y. Chang, H. Xu, W. Yan, Electrochemical phosphorus recovery from wastewater: a critical analysis of mechanisms, process optimization, and future pathways toward industrial application, Chem. Eng. J. 2025, 512, 162468.

[57] J. Xue, W. Verstraete, B.-J. Ni, J.P. Giesy, G. Kaur, D. Jiang, E. McBean, Z. Li, H.-M. Shin, F. Xiao, Y. Liu, J. Liu, L. Chibwe, K.T. Wai Ng, Y. Uchida, Rethink biosolids: Risks and opportunities in the circular economy, Chem. Eng. J. 2025, 510, 161749.

[58] C.J. Ruiken, G. Breuer, E. Klaversma, T. Santiago, M.C.M. van Loosdrecht, Sieving wastewater – Cellulose recovery, economic and energy evaluation, Water Res. 2013, 47, 43-48.

[59] M. Zheng, Z. Hu, T. Liu, M. Sperandio, E.I.P. Volcke, Z. Wang, X. Hao, H. Duan, S.E. Vlaeminck, K. Xu, Z. Zuo, J. Guo, X. Huang, G.T. Daigger, W. Verstraete, M.C.M. van Loosdrecht, Z. Yuan, Pathways to advanced resource recovery from sewage, Nat. Sustain. 2024, 7, 1395-1404.
